# Development of benzo[1,4]oxazines as biofilm inhibitors and dispersal agents against *Vibrio cholerae* †
†Electronic supplementary information (ESI) available: Experimental procedures and analytical data along with protocols for all biological experiments. See DOI: 10.1039/c4cc07003h



**DOI:** 10.1039/c4cc07003h

**Published:** 2014-12-05

**Authors:** Christopher J. A. Warner, Andrew T. Cheng, Fitnat H. Yildiz, Roger G. Linington

**Affiliations:** a Department of Chemistry and Biochemistry , University of California Santa Cruz , California , 95064 , USA . Email: rliningt@ucsc.edu; b Department of Microbiology and Environmental Toxicology , University of California Santa Cruz , California , 95064 , USA

## Abstract

Bacterial biofilms are estimated to be associated with over 65 percent of all nosocomial infections. However, no therapeutics have been approved by the FDA which directly mediate biofilm formation or persistence. Herein we report oxazine **25** as a highly potent inhibitor, disperser and in the presence of the appropriate antibiotic eradicator of *V. cholerae* biofilms.

## Abstract 2

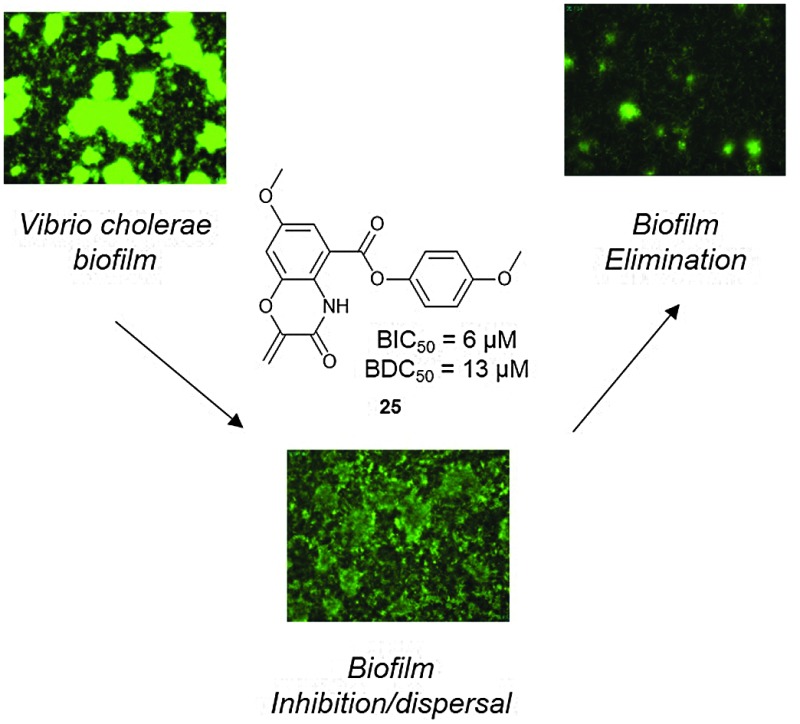
Synthesis of a library of natural product-inspired biofilm inhibitors has revealed a compound with selective and potent anti-biofilm activity against *V. cholerae*.

Bacterial biofilms are surface associated bacterial communities of sessile cells encased in a matrix of polysaccharides, extracellular DNA and proteins.^1^ Such biofilms are of significant concern in nosocomial infections, where it is attributed to over $1 billion in increased hospital costs per annum in the US alone.^2^ Unlike cells in the planktonic state, bacterial biofilms do not exert their antimicrobial resistance through mutation or acquisition of resistance functions by horizontal gene transfer.^3^ Instead, resistance is largely driven by the formation of latent cells within the biofilm matrix that reduce cellular turnover and therefore remove the susceptibility of targets associated with traditional antimicrobials.^4^

*V. cholerae* is a diarrheal pathogen that naturally inhabits both fresh and saltwater environments.^5^ In spite of its prevalence, no clinical therapeutics have been approved for use in the US or elsewhere that directly target biofilm formation and persistence. A limited number of small molecule inhibitors of *V. cholerae* biofilms have been reported in the literature, both from natural product screening campaigns and medicinal chemistry development efforts.^6,7^ However in the majority of cases these compounds have been shown to impact quorum sensing (QS) rather than directly targeting processes involved with biofilm matrix production or regulation.

We recently reported the development of two high throughout image-based screens capable of identifying biofilm inhibitors against the Gram-negative pathogens *V. cholerae* and *Pseudomonas aeruginosa*.^8–10^ Screening of our natural product library, compromising of over 6000 prefractions, identified the aureomycin chromophore **1** as a moderate inhibitor of *V. cholerae* biofilms (biofilm inhibitory concentration (BIC_50_) = 63 μM). Given the structural novelty of this scaffold compared with other biofilm inhibitors, and the unusual biofilm inhibitory phenotype observed in the primary screening images, we elected to develop the benzo[1,4]oxazine scaffold through medicinal chemistry optimization in order to identify key elements of the required pharmacophore, and generate analogues with improved potency and pharmacological properties.^11^ Key to this approach was the formation of the α-keto-amide **7** and its subsequent application in a debenzylation–cyclization strategy to form hemi-acetal **8**. Gratifyingly, treatment of the α-ketoamide **7** (formed in 5 steps from the commercially available ester 2) with 2% Pd(OH)_2_ on charcoal and four equivalents of 1,4-cyclohexadiene in ethanol at 50 °C enabled formation of the cyclic hemi-acetal **8** in excellent yield on a multi-gram scale with reaction times of less than 5 minutes. Dehydration of the acetal afforded the target molecule in 7 steps on a multigram scale (Scheme 1).

**Scheme 1:**
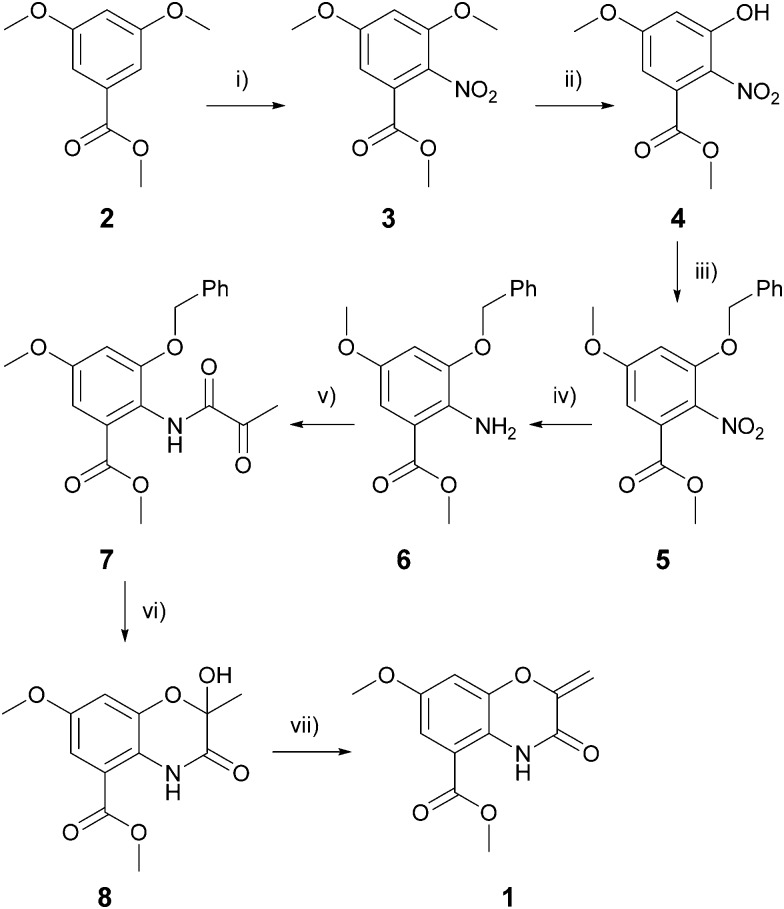
The total synthesis of the benzo[1,4]oxazine biofilm inhibitor **1**. *Reagents and conditions*: (i) HNO_3_, Ac_2_O, 0 °C to rt, 1 hour, 94% yield; (ii) 4 eq. AlCl_3_, DCM, 0 °C to reflux, 3 hours, 89% yield; (iii) 4 eq. BnBr, 4 eq. K_2_CO_3_, 1 : 1 DCM/MeOH, reflux, 3 hours, 97% yield; (iv) 4 eq. SnCl_2_, 3 : 1 EtOH/6N HCl, rt to reflux, 45 minutes, 81% yield; (v) 1.1 eq. pyruvoyl chloride, 1.5 eq. pyridine, DCM, 0 °C to rt, 90 minutes, 65% yield; (vi) 2% Pd(OH)_2_/C, 4 eq. 1,4-cyclohexadiene, EtOH, 50 °C, 2 minutes, 81% yield; (vii) 1.2 eq. MsCl, 1.5 eq. NEt_3_, DCM, 0 °C to rt, 90 minutes, 82% yield.

To identify key elements of the pharmacophore for this compound class, a library of 41 derivatives were prepared using a divergent strategy to diversify both the exocyclic alkene and the methyl ester moiety of the original scaffold (Fig. 1, see ESI† for a comprehensive list of synthesized compounds). Initial examination of the lead compound **1** highlighted the α,β unsaturated carbonyl (C9–C11) as a Michael acceptor with potential involvement in the mechanism of action. In line with the behavior of other Michael acceptors in the literature, modification of the exocyclic methylene unit (compounds **8–12**) eliminated activity in all cases.^12^ Introduction of any substituent onto the double bond (compounds **13–17**) also resulted in a pronounced decrease in biofilm inhibition, indicating a tight steric limitation for modifications at this position.

**Fig. 1:**
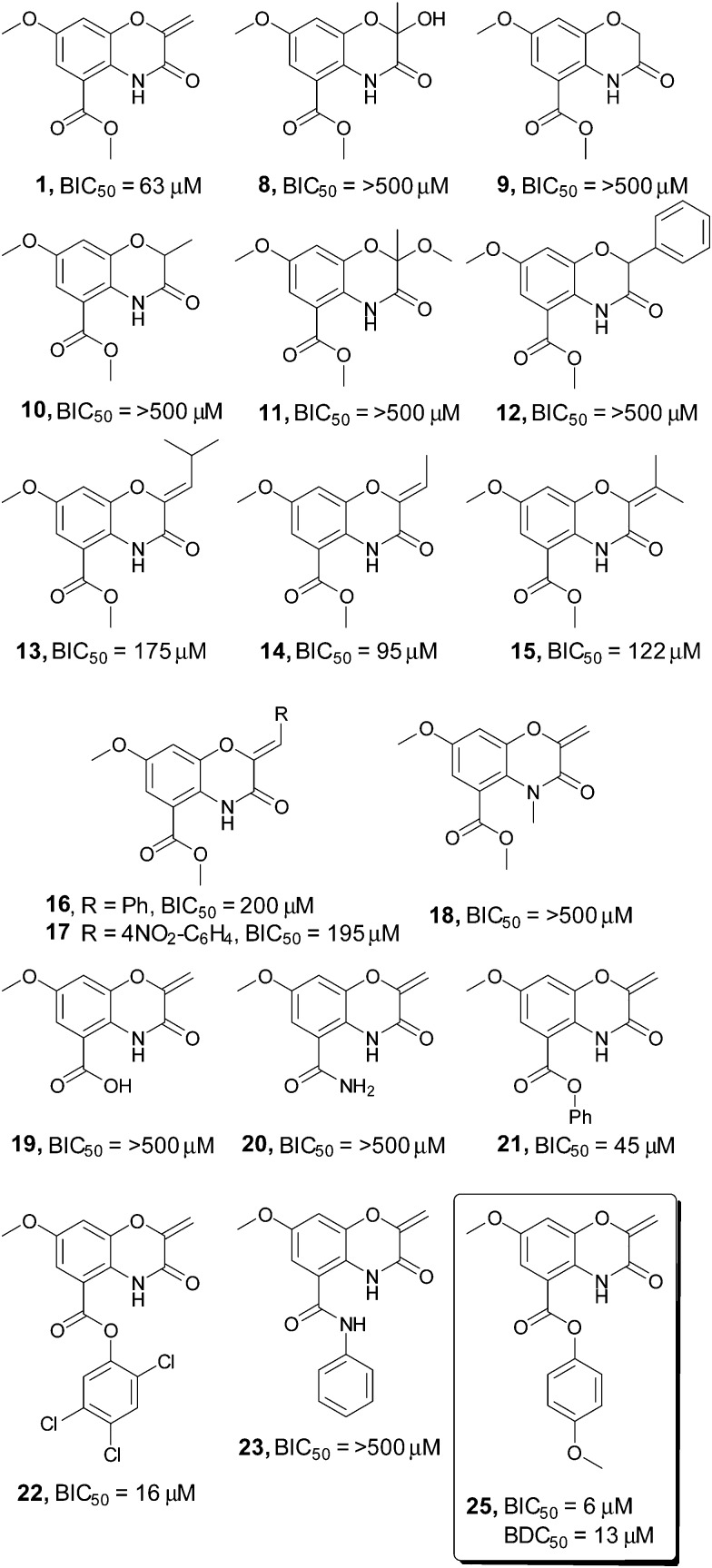
Screening of a selection of the oxazine inhibitors against *V. cholerae* biofilms. A selection of the oxazine derivatives screened as inhibitors of *V. cholerae* biofilms. BIC_50_ and BDC_50_ determined with 3 biological replicates each consisting of two technical replicates, see ESI† for full BIC_50_ dose response curves and complete list of all compounds screened in the assay.

To probe whether the increase in steric size of the Michael acceptor directly correlated with the ability of the compound to undergo Michael addition, both the original oxazine **1** and phenyl substituted compound **16** were added to either *N*-acetyl cysteine methyl ester or a simple polypeptide (Ac-Ala-Cys-Ala-Gly-OH) and the formation of Michael addition adducts monitored by LC-MS. Complete formation of the expected Michael adduct was observed with compound **1** for both substrates. By contrast very little (∼5%) of the expected Michael adducts were observed in either case with compound **16**, suggesting that the tight steric limitation around the alkene may be due to the inability of sterically encumbered compounds to act as Michael acceptors.

A similar loss in activity was observed upon conversion of the endocyclic amide to the corresponding *N*-methyl derivative (compound **18**). This suggests that a hydrogen bond donor may be required to correctly orientate the compound in the active site, or alternatively that this portion of the molecule is present in a congested region of the active site that is not tolerant of increased steric bulk. In contrast to modifications on the oxazine ring, alteration of the ester substituent at C2 resulted in a pronounced increase in biofilm inhibition. Although conversion of the methyl ester to the corresponding free acid **19** eliminated activity, introduction of the *para*-methoxy aromatic residue **25** resulted in a 10-fold increase in compound efficacy, with a BIC_50_ value of 6 μM. Colony forming unit analysis demonstrated that compound **25** possessed no bactericidal activity up to 200 μM (see ESI†). This was confirmed by BioMAP screening, with compound **25** displaying no activity against a panel of 15 clinically relevant bacterial pathogens up to 200 μM.^12^ Screening in our in-house image-based cytological profiling assay also revealed that compound **25** had no measurable cytotoxicity to HeLa cells, up to 200 μM (see ESI†).^13^ Interestingly, the parent phenyl compound **21** showed only a marginal increase in activity (45 μM *vs.* 62 μM), while the corresponding substituted phenyl ester **22** exhibited a 4-fold increase in activity over the parent methyl ester **1**, demonstrating the importance of substituent effects on the aromatic ring.

The observation that the carboxylic acid **19** was inactive as a biofilm inhibitor led us to probe whether hydrolysis of the phenolic ester could be masking the true potency of this compound class. Incubation of oxazine **25** in either LB media or PBS buffer at 37 °C for 24 hours failed to result in any measurable hydrolysis and suggested that this was not a limiting factor for compound activity. Interestingly, formation of either the phenyl amide **23** or the analogous *para*-methoxy amide, two compounds that would be expected to be far more resistant to hydrolysis resulted in a complete loss in compound activity.

One potential application for biofilm inhibitors is as dispersal agents to eliminate biofilms in established infections, which otherwise persist during antibiotic treatment and can lead to recrudescence of infection. We have recently disclosed a novel biofilm dispersal model (BDM) in *P. aeruginosa* that can be used to examine the capabilities of small molecules to induce dispersion of pre-existing biofilms.^10^ To investigate whether compound **25** was capable of the dispersal of pre-formed *V. cholerae* biofilms, a similar procedure was employed in the *V. cholerae* system. In brief, cultures of *V. cholerae* were allowed to pre-form biofilms in screening plates for 2 hours prior to compound addition. Following standard incubation conditions (4 hours at 30 °C) macrocolonies were imaged and quantified as previously described to determine the percentage of remaining biofilm coverage. Compound **25** displayed strong biofilm dispersal activity and no bactericidal activity, with a biofilm dispersal concentration (BDC_50_) value of 13 μM and optical density readings indicating good bacterial growth. To our knowledge this represents the first example of a small molecule capable of both inhibiting and inducing dispersal of *V. cholerae* biofilms, and places it among just a handful of compounds capable of inducing the dispersal of mature surface-associated biofilms.^14^

A major challenge surrounding the treatment of biofilm-mediated infections is that bacterial cells within the biofilm have the potential to enter a latent state that renders them much less susceptible to traditional antibiotics.^4^ One potential application for biofilm dispersal agents is as combination therapies with existing antibiotics to both clear and eliminate otherwise persistent infections. To examine whether our biofilm dispersal model could recapitulate this antibiotic resistance for *V. cholerae* we screened five FDA-approved antibiotics (ciprofloxacin, erythromycin, azithromycin, doxycycline and furazilidinone) in the dispersal assay. Interestingly, without the presence of compound **25**, erythromycin, ciprofloxacin and furazilidinone (all therapeutics prescribed as first stage treatments for *V. cholerae* infection) failed to induce biofilm dispersal, with confocal microscopy indicating the presence of large biofilm macrocolonies and very few background planktonic cells. Optical density readings confirmed this observation and suggested that these antibiotics have the capacity to eradicate cells in the planktonic state, but not significantly impact biofilm coverage. By contrast, addition of 20 μM of compound **25** in addition to 50 μM of either erythromycin or ciprofloxacin resulted in near quantitative elimination of biofilm coverage, and a lowering in the cellular viability, as determined by OD_600_ analysis, indicating that these drug combinations possess the ability to both clear and kill established *V. cholerae* infections (Fig. 2). One limitation of image-based high content screening is that samples must be incubated under static culture conditions. The disadvantage of this method is that static culture allows the accumulation of signaling factors and quorum sensing molecules including *V. cholerae* autoinducer-1 (CAI-1), autoinducer-2 (AI-2) and indole which all impact the rate and degree of biofilm formation.^15,16^

**Fig. 2:**
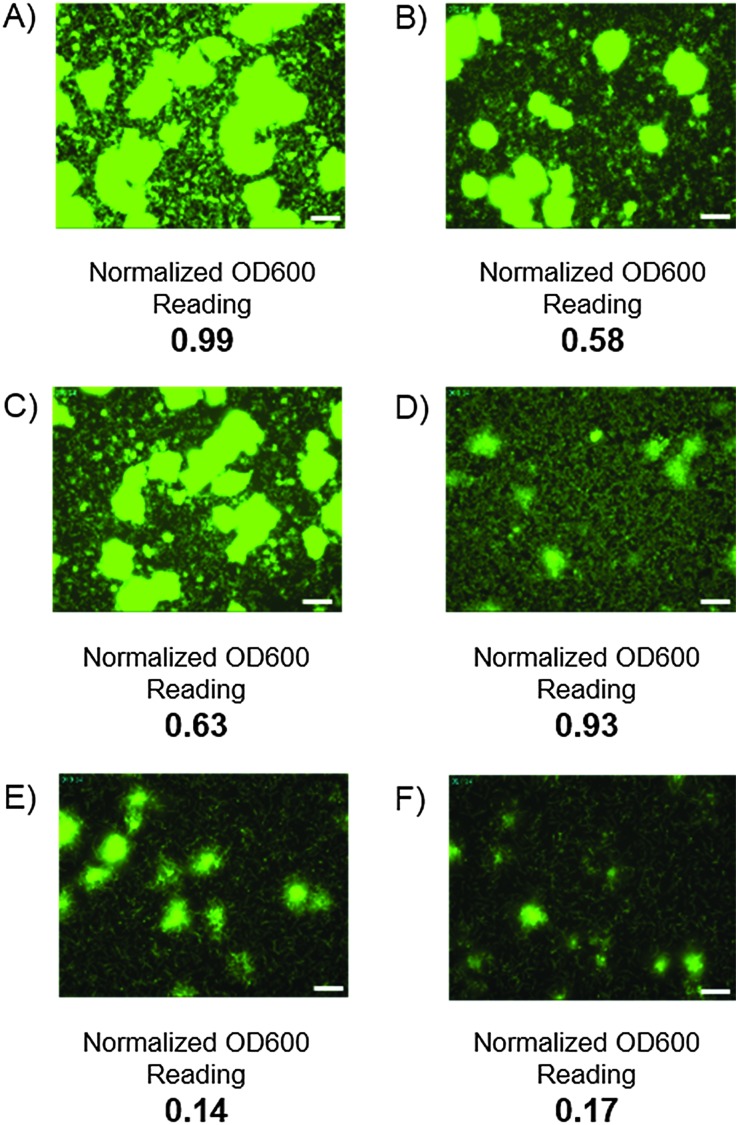
Static well images of *V. cholerae* biofilms. Static well GFP images of *V. cholerae* biofilms and normalized OD_600_ readings. In all instances the antibiotic, dispersal agent or a combination of the two were introduced after two hours of incubation and incubated for a further 4 hours before being washed and analyzed. (A) DMSO control; (B) 50 μM ciprofloxacin; (C) 50 μM erythromycin; (D) 20 μM compound **25**; (E) 20 μM compound **25** and 50 μM ciprofloxacin; (F) 20 μM compound **25** and 50 μM erythromycin. White bars represent 50 μm.

To examine whether oxazine **25** was capable of disrupting biofilm formation and persistence under more physiologically relevant conditions, we examined its anti-biofilm properties under flow cell conditions. At all concentrations tested (see ESI†), strong biofilm inhibition was observed, with a marked decrease in the size, thickness and morphology of the biofilm. COMSTAT analysis^17^ indicated a 7-fold reduction in biomass upon treatment of the *V. cholerae* biofilms with compound **25** under flow conditions (Fig. 3B). These data indicate that compound **25** is capable of disrupting biofilms under both static and flow cell conditions, and suggest that this compound has potential value as a biofilm inhibitor for the clearance of established biofilm-mediated infections *in vivo*.

**Fig. 3:**
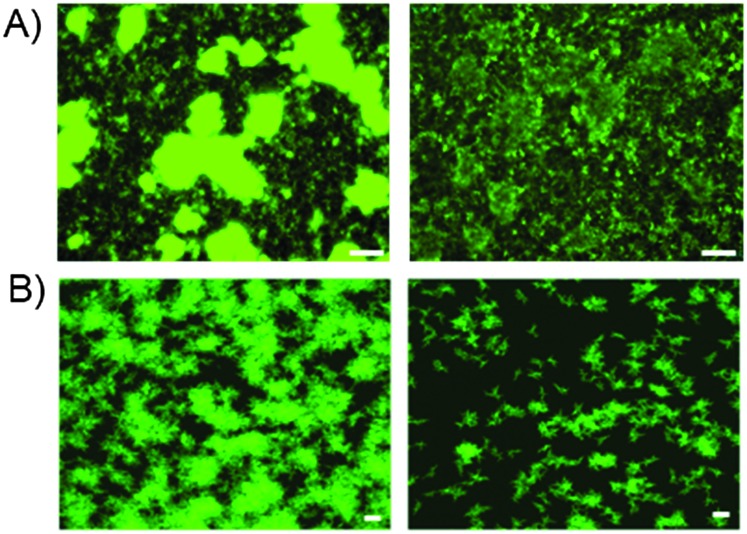
Static and confocal flow cell images of compound **25** against *V. cholerae biofilms*. (A) static culture conditions, from left to right, DMSO control, compound **25** at 15 μM. In both instances OD_600_ data suggested no bactericidal activity; (B) confocal flow cell images of *V. cholerae*, see ESI† for further details. From left to right, DMSO control and lead compound **25** at 250 μM. COMSTAT analysis of mean biomass (mm^3^/mm^2^) indicated a 7-fold reduction in the presence of lead compared **25** compared to DMSO control. In both cases white bars indicate 50 μm.

In conclusion, a medicinal chemistry approach is reported for the development of the *V. cholerae* biofilm inhibitor **25** that involves a 7 step, multi-gram, purification-free route to primary lead **1**. An SAR study on the oxazine scaffold identified the importance of both the exocyclic alkene and amidic proton for compound activity. Modifications at either position led to complete loss of activity for all tested compounds. However, expansion around the methyl ester identified phenyl ester derivative **25** as an advanced lead compound with one of the highest potencies reported to date for biofilm inhibition against *V. cholerae* (BIC_50_ = 6 μM) and the capacity to induce the dispersal of preformed biofilms (BDC_50_ = 13 μM). Co-dosing of compound **25** with 50 μM erythromycin or azithromycin demonstrated the potential of the synergistic action of a dispersal reagent and antibiotic in inducing detachment and subsequent clearance of preformed biofilms. Such a strategy may offer a means of overcoming both the tolerance of biofilms toward traditional antibiotics, and reduce the potential for the development of bacterial resistance often observed with antibiotic mono-therapies.
