# Supplementary material for: Development of benzo[1,4]oxazines as biofilm inhibitors and dispersal agents against Vibrio cholerae
Source: Chem Commun (Camb). 2014 Dec 5;51(7):1305–8. doi: 10.1039/c4cc07003h (PMC4288701; doi:10.1039/c4cc07003h)
Supplement: Supplementary file 1 [file CC-051-C4CC07003H-s001.pdf]

Electronic Supplementary Material (ESI) for ChemComm.
This journal is © The Royal Society of Chemistry 2014

Development of benzo[1,4]oxazines as potent biofilm inhibitors and dispersal agents against *Vibrio cholerae*.

Christopher J. A. Warner,[a] *Andrew T. Cheng*[b], *Fitnat H. Yildiz*[b], *Roger G. Linington*[a]*

[a]Department of Chemistry and Biochemistry; [b]Department of Microbiology and Environmental Toxicology,
University of California Santa Cruz, 1156 High Street, Santa Cruz, CA, 95064
E-mail: rliningt@ucsc.edu

Contents

General remarks	S2
Complete list of compounds tested	S3 - S5
BIC_{50} curves for active compounds	S6 – S7
EC_{50} curves of the antibiotics used in the co-dosing experiments	S8 - S10
Flow cell experiments of oxazine 25	S11
BioMAP antibacterial profiling of oxazine 25	S12
CFU analysis of oxazine 25	S13
HeLa cell line toxicity study of oxazine 25	S14
Stability study of oxazine 25 in culture media	S15
Experimental procedures	S16 - S32
^{1}H and ^{13}C NMR spectra	S33 – S73
References	S74

General remarks

All reactions were performed in an open flask using acetone washed, oven dried glassware with magnetic stirring and if required heated through the use of Dry Syn™ blocks. All reagents used were acquired from chemical supply companies or, as indicated in the individual experimental details, prepared within the laboratory. Reactions that were performed at 0 °C were done so using water/ice baths. All solvents used in the course of the project were obtained from the departmental Grubbs solvent system.

Analytical thin layer chromatography (TLC) was carried out utilizing aluminium backed Merck TLC plates (silica gel 60 F254) and visualized with UV light (254 nm) or basic $KMnO_4$ solution. Flash column chromatography was performed using Alfa Aesar, silica gel 60, 0.032 – 0.063 mm (230 – 450 mesh) as the stationary phase. Columns were typically packed as a slurry, with the eluent used for a particular purification noted within the individual experimental details for each reaction.

All ^{1}H and ^{13}C NMR spectra were obtained on either a Varian Unity 500+ or a Varian Inova 600 MHz spectrometer equipped with a 5 mm HCN triple resonance cryoprobe. Chemical shifts are expressed in parts per million (ppm) downfield from tetramethylsilane (TMS). All coupling constants given are in Hz. High resolution mass spectrometry was performed on an Agilent 6230 electrospray ionization (ESI) accurate-mass time-of-flight (TOF) liquid chromatograph/ mass spectrometer.

All procedures for determination of the biofilm inhibitory concentration (BIC_{50}) occurred as previously described.[1] See individual experimental details for antibiotic and co-dosing procedure. All BIC_{50} and BDC_{50} reported are the result of three biological replicates each consisting of two technical replicates.

Table 1. A complete list of the compounds screened as biofilm inhibitors against *Vibrio cholerae*

compound[a]	structure	BIC_{50} / μM[b]	BDC_{50} / μM[c]	compound[a]	structure	BIC_{50} / μM[b]	BDC_{50} / μM[c]
**1**	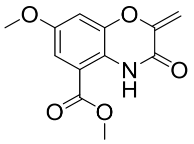	63	-	**15**	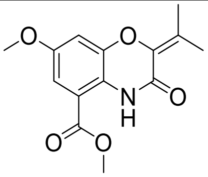	122	-
**8**	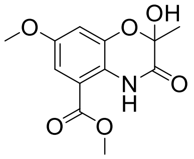	>500	-	**16[c]**	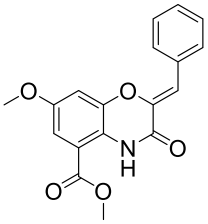	200	-
**9**	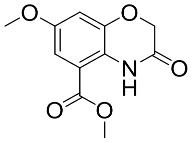	>500	-	**17[c]**	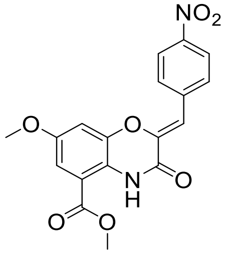	195	-
**10**	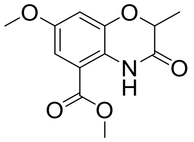	>500	-	**18**	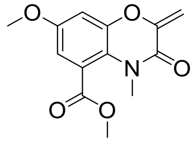	>500	-
**11**	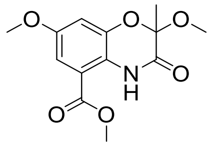	>500	-	**19**	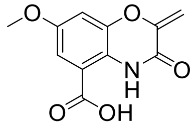	>500	-
**12**	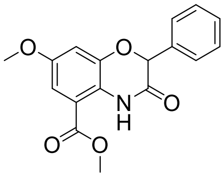	>500	-	**20**	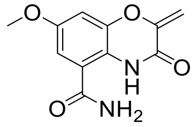	>500	-
**13[b]**	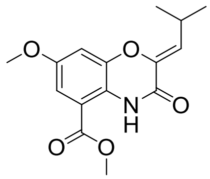	175	-	**21**	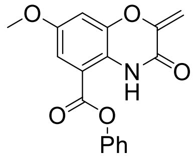	45	-
**14[c]**	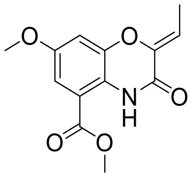	95	-	**22**	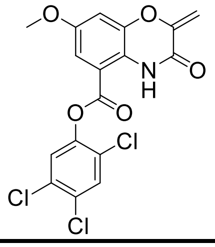	13	-

Table 1 continued. A complete list of the compounds screened as biofilm inhibitors against *Vibrio cholerae*

compound	structure	BIC_{50} / μM	BDC_{50} / μM	compound	structure	BIC_{50} / μM	BDC_{50} / μM
**24**	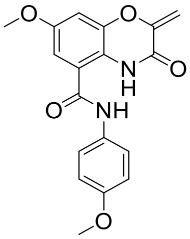	>500	-	**30**	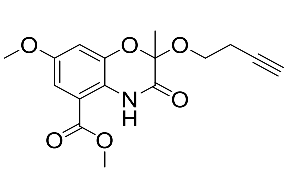	>500	-
**23**	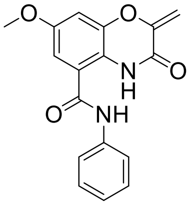	>500	-	**31**	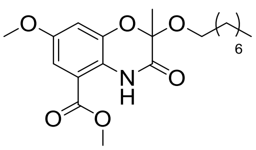	>500	-
**25**	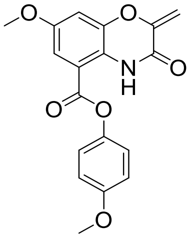	6	13	**32**	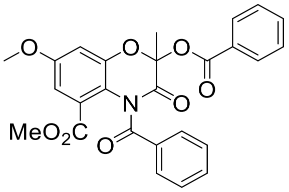	>500	-
**26**	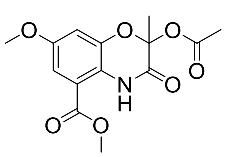	>500	-	**33**	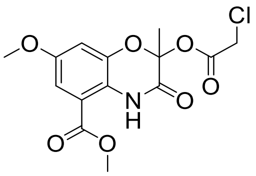	>500	-
**27**	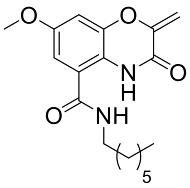	>500	-	**34**	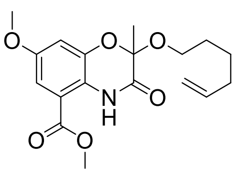	>500	-
**28**	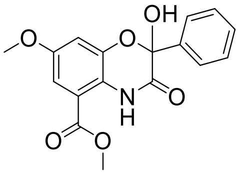	>500	-	**35**	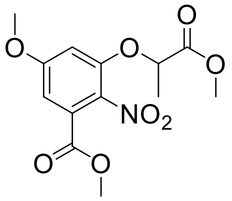	>500	-
**29**	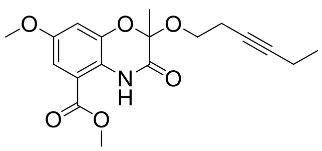	>500		**36**	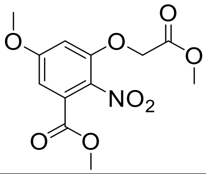	>500	-

Table 1 continued. A complete list of the compounds screened as biofilm inhibitors against *Vibrio cholerae*

compound	structure	BIC_{50} / µM	BDC_{50} / µM	compound	structure	BIC_{50} / µM	BDC_{50} / µM
**37**	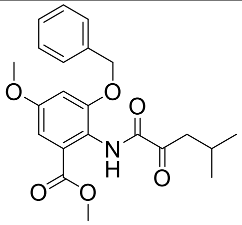	>500	-	**3**	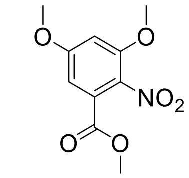	>500	-
**38**	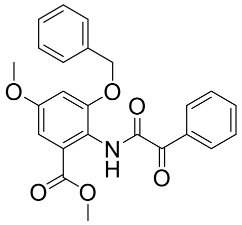	>500	-	**4**	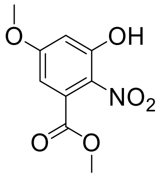	>500	-
**39**	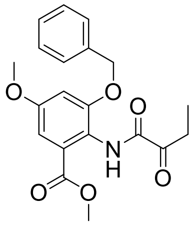	>500	-	**5**	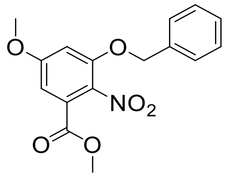	>500	-
**40**	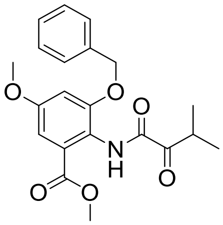	>500	-	**6**	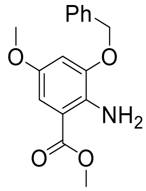	>500	-
**41**	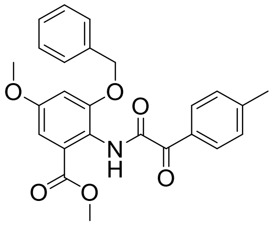	>500	-	**7**	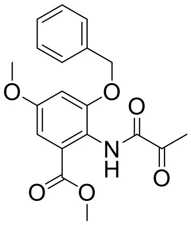	>500	-

[a] BIC_{50} and BDC_{50} determined with 3 biological replicates each consisting of two technical replicates. For the biofilm dispersal assay, the appropriate compound, antibiotic or DMSO control was pinned into the well following two hours of incubation and then subsequently incubated for a further 4 hours. [b] Major isomer shown. Stereochemistry of the major isomer determined by long range nOe interaction as shown in subsequent section of SI. [c] Major isomer assumed based upon nOe interaction observed in oxazine **13**.

BIC$_{50}$ and BDC$_{50}$ curves for active biofilm inhibitors and dispersal agents

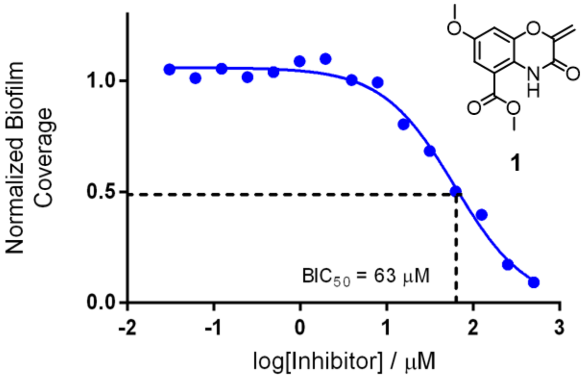

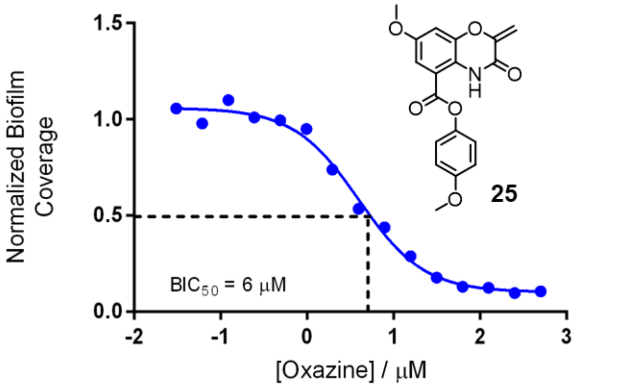

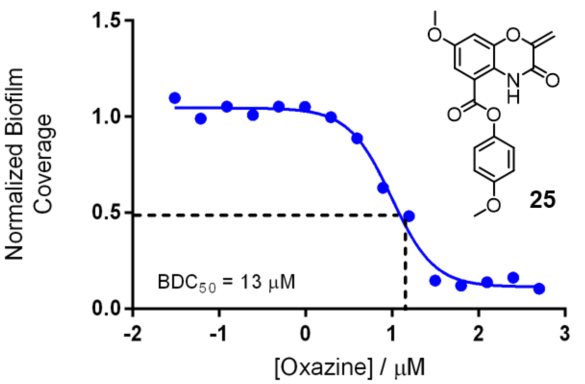

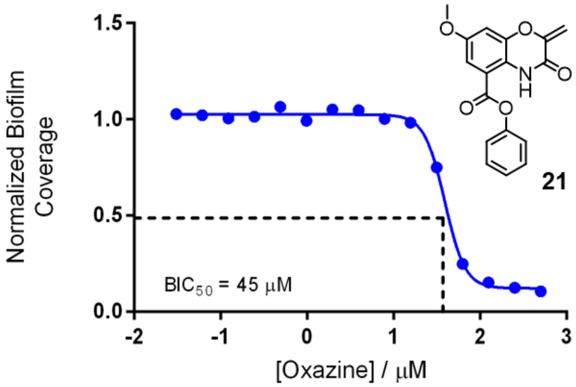

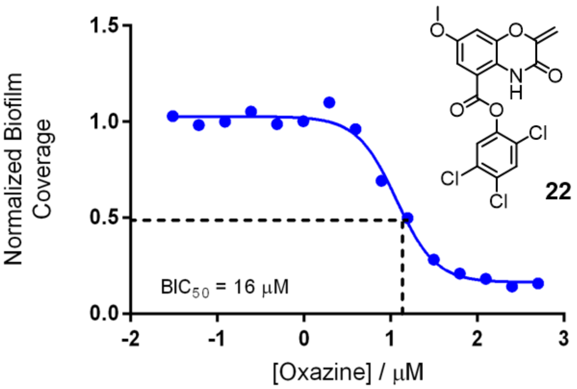

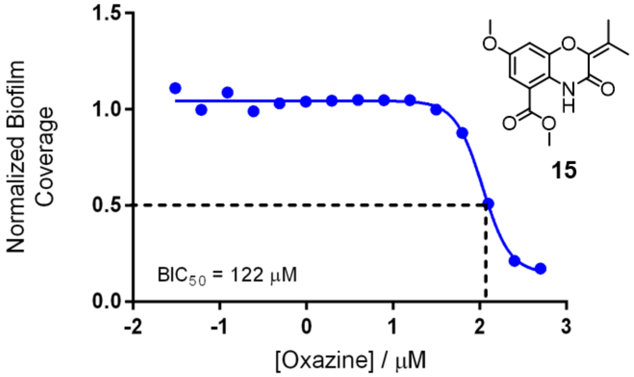

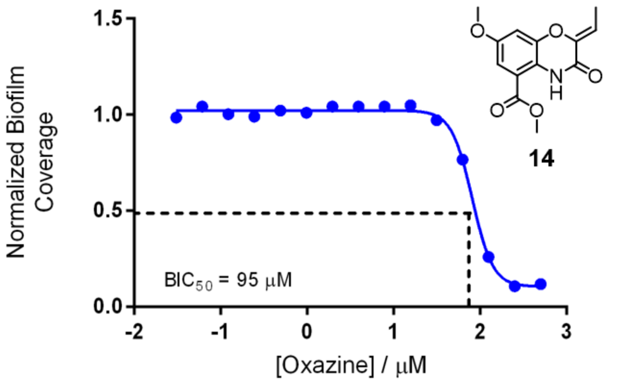
1.5
1.0
0.5
0.0
Normalized Biofilm Coverage
BIC$_{50}$ = 95 µM
-2
-1
0
1
2
3
[Oxazine] / µM

14

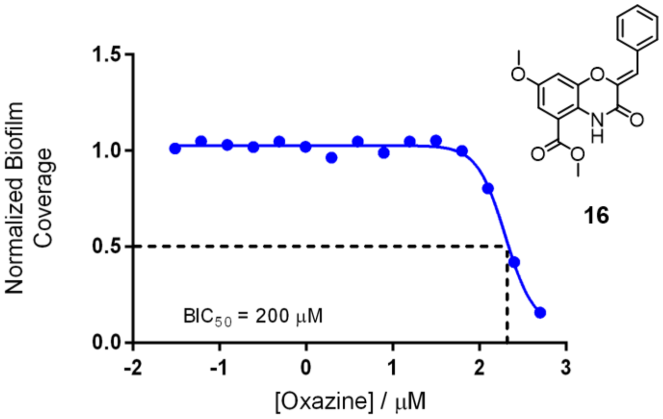
1.5
1.0
0.5
0.0
Normalized Biofilm Coverage
BIC$_{50}$ = 200 µM
-2
-1
0
1
2
3
[Oxazine] / µM

16

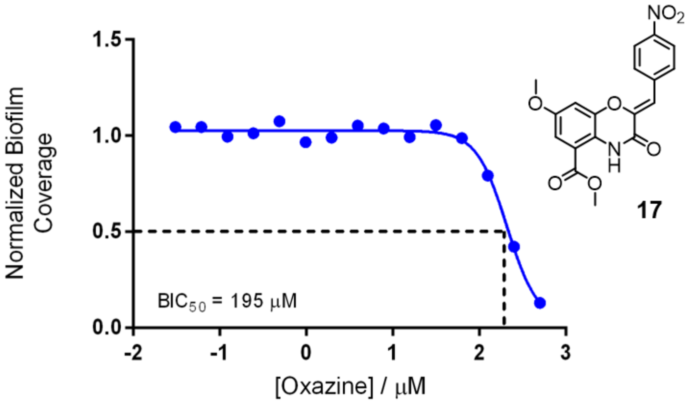
NO$_2$
1.5
1.0
0.5
0.0
Normalized Biofilm Coverage
BIC$_{50}$ = 195 µM
-2
-1
0
1
2
3
[Oxazine] / µM

17

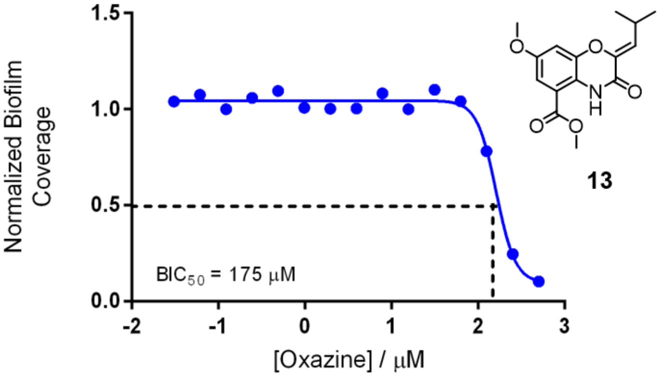
1.5
1.0
0.5
0.0
Normalized Biofilm Coverage
BIC$_{50}$ = 175 µM
-2
-1
0
1
2
3
[Oxazine] / µM

13

EC$_{50}$ curves of the antibiotics used in the co-dosing experiments

Preformed Biofilm Screening Overview

Experimental procedure

The preformed biofilm screen followed the general experimental procedure developed in P. aeruginosa.[1] For the V. cholerae biofilm dispersal assay (BDC$_{50}$), compound, antibiotic or DMSO control were pinned into the screening plate following two hours of incubation and incubated for a further 4 hours at 32°C. An identical washing and analytical procedure to that reported in the literature was performed.[1] For the antibiotic co-dosing experiments, both oxazine **25** and the relevant antibiotic were added after 2 hours of incubation and OD$_{600}$ readings immediately taken to determine initial OD$_{600}$ values. After incubation, OD$_{600}$ readings were acquired, and the change in OD$_{600}$ values used as a measure of cell growth for each well. Immediately following OD$_{600}$ readings, the plates were washed, PBS buffer added, and each well imaged using our standard protocol to determine biofilm coverage.

Data interpretation

In both the biofilm inhibition and dispersal assays, four outcomes are possible for any assay well. In the case of strong antibiotic activity, both planktonic and attached cells are eliminated, and the resulting screening images are blank, with low OD$_{600}$ readings (Image A). For compounds capable of eradicating only the planktonic cells without impacting attached cells a lower OD$_{600}$ reading would be expected, but with retention of large biofilm colonies in the image (Image B). If the compound has no effect on planktonic or biofilm-associated cells, then both the OD600 and biofilm coverage are high (Image C). Finally if the compound is capable of only inducing detachment of the bacteria with no bactericidal effects then an OD$_{600}$ reading of close to 1.0 would be expected and cellular imaging would show only planktonic cells, without the presence of large mature biofilm colonies (Image D).

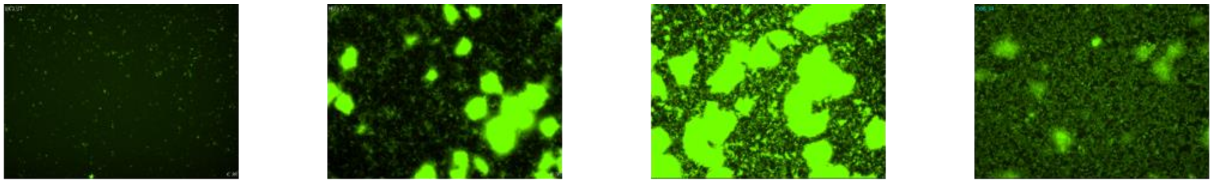

Image **A**
Dark well, no attached cells or planktonic bacteria present

Expected normalized OD$_{600}$ reading close to 0

Image **B**
Dark background, no planktonic bacteria present. Large colonies of attached cells still present

Expected normalized OD$_{600}$ reading close to 0.6

Image **C**
Both planktonic and attached cells present. No bactericidal effects observed.

Expected normalized OD$_{600}$ reading close to 1.0

Image **D**
Only detached cells present, no large colonies of attached cells. No bactericidal effects observed

Expected normalized OD$_{600}$ reading close to 1.0

Addition of antibiotic at t = 0

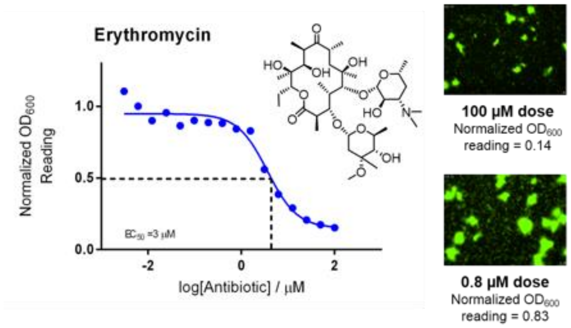

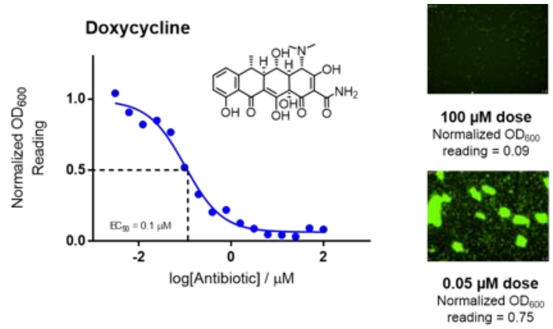

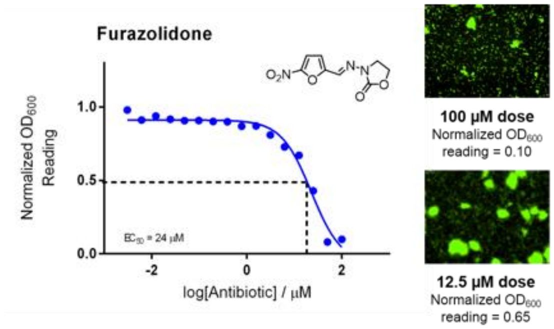

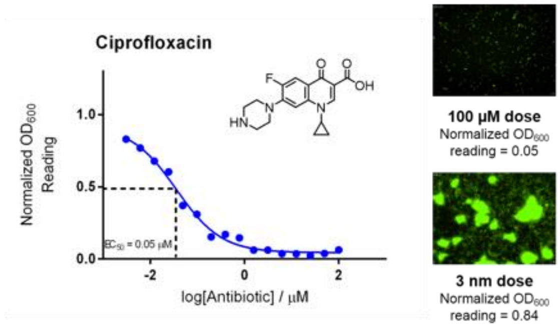

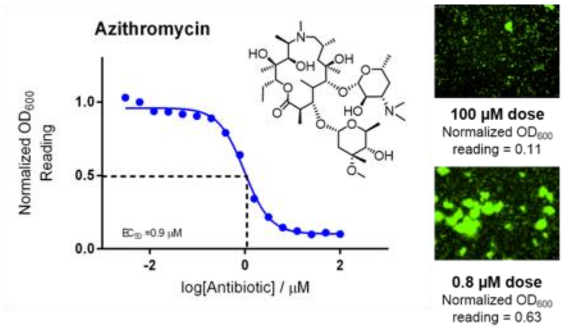

Addition of antibiotic at t = 2

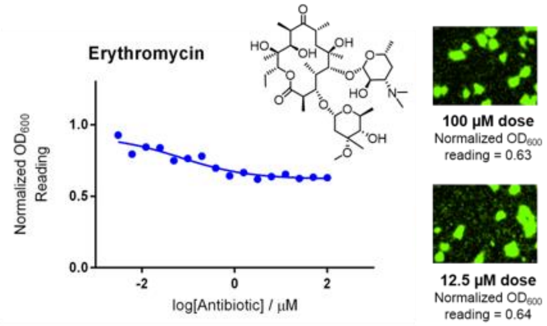

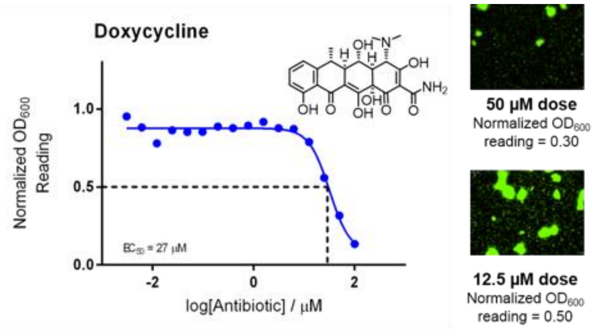

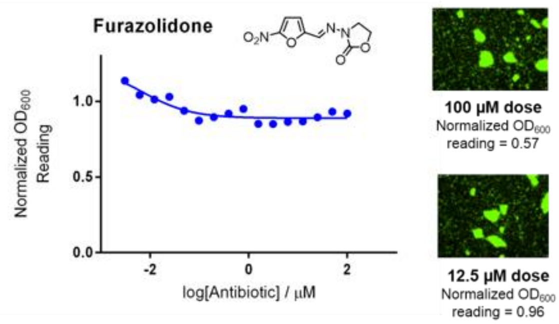

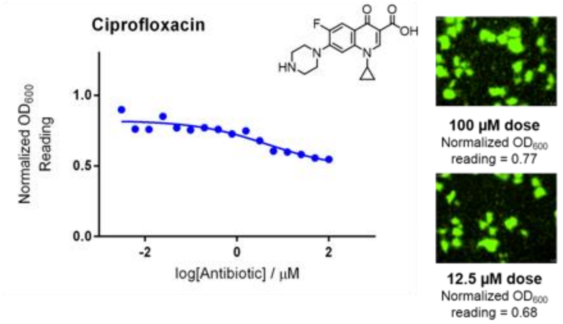

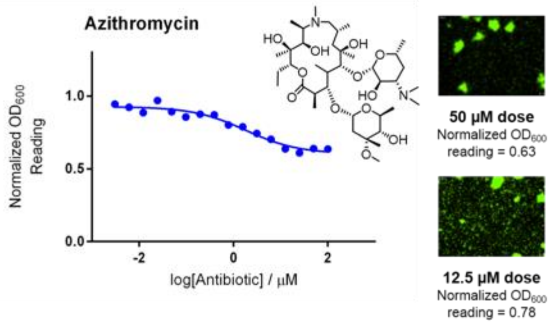

<u>Flow cell experiments of oxazine **25**</u>

Overnight culture of rugose wild type *V. cholerae* (A1552 harboring a Tn7GFP insertion) was diluted 200-fold into 2% LB medium containing either the indicated concentration of test compound or an equal volume of DMSO as a vehicle control and inoculated into an Ibidi $\mu^{0.4}$ 6-well flow cell. After 1 hour of static incubation at room temperature, flow of 2% LB containing test compound or DMSO was initiated at 7.5 mL/minute at room temperature for 6 hours. Flow cells were imaged on a Zeiss LSM5 confocal microscope. Z-projections of Z-stacks were created with the FIJI build of Image J. Quantitative analysis of images was performed with COMSTAT.[2]

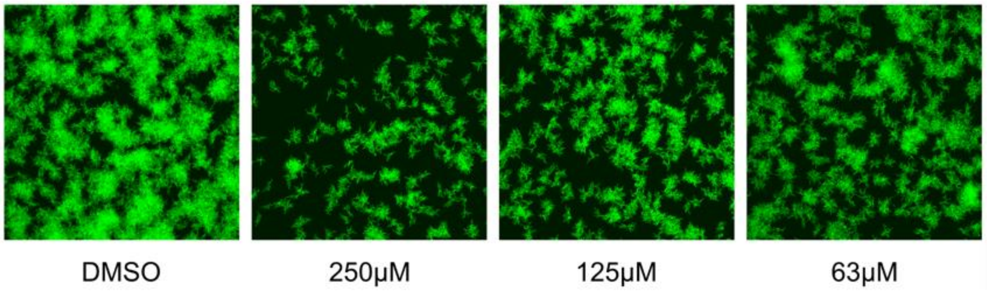

Compound 25 / μM	0	63	100	125	200	250
Mean Biomass ($\mu m^3/\mu m^2$)	2.939	1.866	1.411	1.480	0.463	0.880
Std. Deviation	0.294	0.206	0.234	0.208	0.281	0.173
Fold reduction		1.58	2.08	2.09	6.35	3.34

BioMAP antibacterial profiling of oxazine **25**

The antibacterial profiling of oxazine **25** followed that previously reported in the literature.[3] In brief, the screening panel consisted of six Gram-positive strains (BSL1: *Bacillus subtilis* 168, *Staphylococcus epidermis* [ATCC 14990], *Enterococcus faecium* [ATCC 6569], *Listeria ivanovii* [BAA-139]; BSL2: *S. aureus* [ATCC 29213], methicillin-resistant *S. aureus* (MRSA) [BAA-44] and nine Gram-negative strains (BSL1: *Escherichia coli* K12 [BW 25113], *Acinetobacter baumanii* [NCIMB 12457], *Enterobacter aerogenes* [ATCC 35029], *Ochrobactrum anthropi* [ATCC 49687], *Providencia alcalifaciens* [ATCC 9886]; BSL2: *Yersinia pseudotuberculosis* [IP2666 pIBI], *Pseudomonas aeruginosa* [ATCC 27835], *Salmonella typhimurium* LT2, *Vibrio cholerae* O1 [biotype El Tor A1552, smooth variant (Fy_Vc_1)].

All staphylococcal strains, *L. ivanovii* and *E. faecium* cultures were grown in 10 mL of tryptic soy broth (17 g tryptone, 3 g soytone, 2.5 g dextrose, 5 g NaCl and 2. 5 g dipotassium phosphate in 1 L distilled water; pH 7.5). *P. alcalifaciens*, *O. anthropi*, *E. aerogenes* and *A. baumanii* were grown in nutrient broth (Difco, USA) while *B. subtilis*, *E. coli*, *V. cholerae*, *S. typhimurium*, *P. aeruginosa* and *Y. pseudotuberculosis* cultures were grown in Luria Broth (10 g tryptone, 5 g yeast extract and 10 g NaCl in 1 L distilled water; pH 7.5). All three media were autoclaved at 121°C for 30 min. Inoculated cultures were grown overnight in a shaker (200 rpm; 30°C).

Overnight saturated cell cultures of pathogenic strains were diluted 1:1000 with fresh media and 30 µL of culture was dispensed into each well of sterile clear 384-well plates. 200 nL of DMSO prefraction stock solutions were pinned into screening plates using a Perkin Elmer Janus MDT robot. After inoculation, screening plates were stacked in a plate reader/shaker (Perkin Elmer EnVision) and OD_{600} readings taken once per hour for 24 h. Computer generated growth curves for serially diluted pure compounds were used to determine MIC values by correlating the OD_{600} reading at the pre-exponential phase of the bacteria to the concentrations in individual wells.

Pathogen	MIC of oxazine **25** / µM[a]
Bacillus subtilis	>200
Staphylococcus epidermis	>200
Enterococcus faecium	>200
Listeria ivanovii	>200
S. aureus	>200
MRSA	>200
Escherichia coli	>200
Acinetobacter baumanii	>200
Enterobacter aerogenes	>200
Ochrobactrum anthropi	>200
Providencia alcalifaciens	>200
Yersinia pseudotuberculosis	>200
Pseudomonas aeruginosa	>200
Salmonella typhimurium	>200
Vibrio cholerae	>200

<u>CFU analysis of oxazine **25**</u>

Overnight grown cultures of *V. cholerae* O1, El Tor A1552, rugose variant (Fy_Vc_2) were diluted 1:1000 in the presence of 200 µM, 50 µM and 6 µM of oxazine **25** in LB medium. Cultures were incubated at 30 °C with shaking at 200 rpm. Samples were harvested at specific time points and plated to enumerate CFU/ml. A negative control of doxycycline at 10 µM was also utilized. In all instances growth of *V. cholerae* in the presence of the oxazine **25** was comparable to that of the DMSO control vehicle. It should be noted that at the 24 hour time point a depreciation in CFU is observed. This is typical for such experiments.

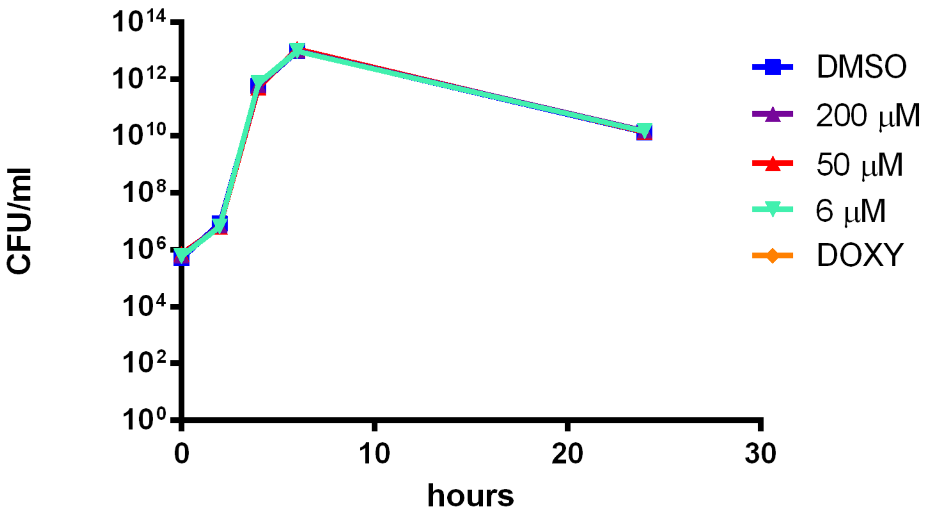

HeLa cell line toxicity study of oxazine **25**

Cytological profiling was performed as previously described.[4] Plates were imaged using an ImageXpress Micro epifluorescent microscope (Molecular Devices, LLC) with a 10× Nikon objective lens. Images were analysed using MetaXpress (Molecular Devices, LLC). In all instances up to 200 µM, oxazine **25** exerted no toxicity toward HeLa cells, with comparable cellular counts compared to the DMSO control vehicle (see below). White bar indicates a distance of 100 µm.

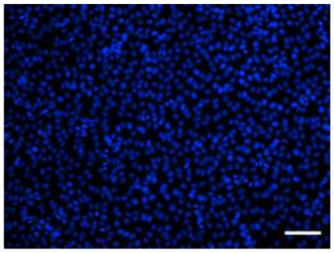

DMSO control vehicle

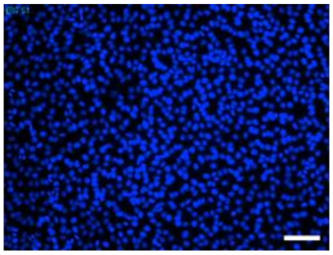

Oxazine **25** at 200 µM

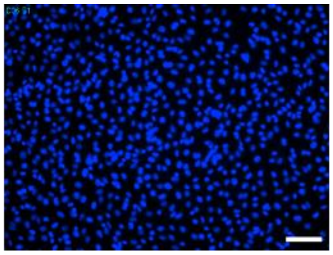

Oxazine **25** at 100 µM

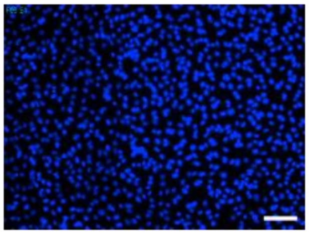

Oxazine **25** at 42 µM

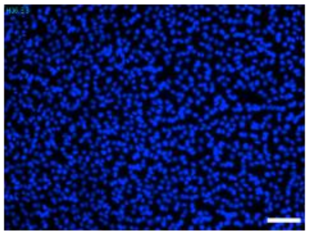

Oxazine **25** at 5 µM

Stability study of oxazine **25** in culture media

Oxazine **25** (5 mg, 0.02 mmol) was dissolved in DMSO (100 µl) and added in a single portion to the appropriate culture media and, if appropriate, heated to 37 °C. In all instances agitation of the mixture was obtained by mechanical stirring. Following overnight incubation, the solution was diluted with methanol (5 mL) and subjected to reverse-phase HPLC using a Phenomenex synergi-A 10µ fusion C_{18} column. An isocratic gradient of 6:4 Methanol/H_2O (acidified with 0.02% of formic acid) was used as the solvent system. A wavelength of λ = 254 nm was used in all instances. The oxazine **25** was identified to have a retention time of 7.0 minutes.

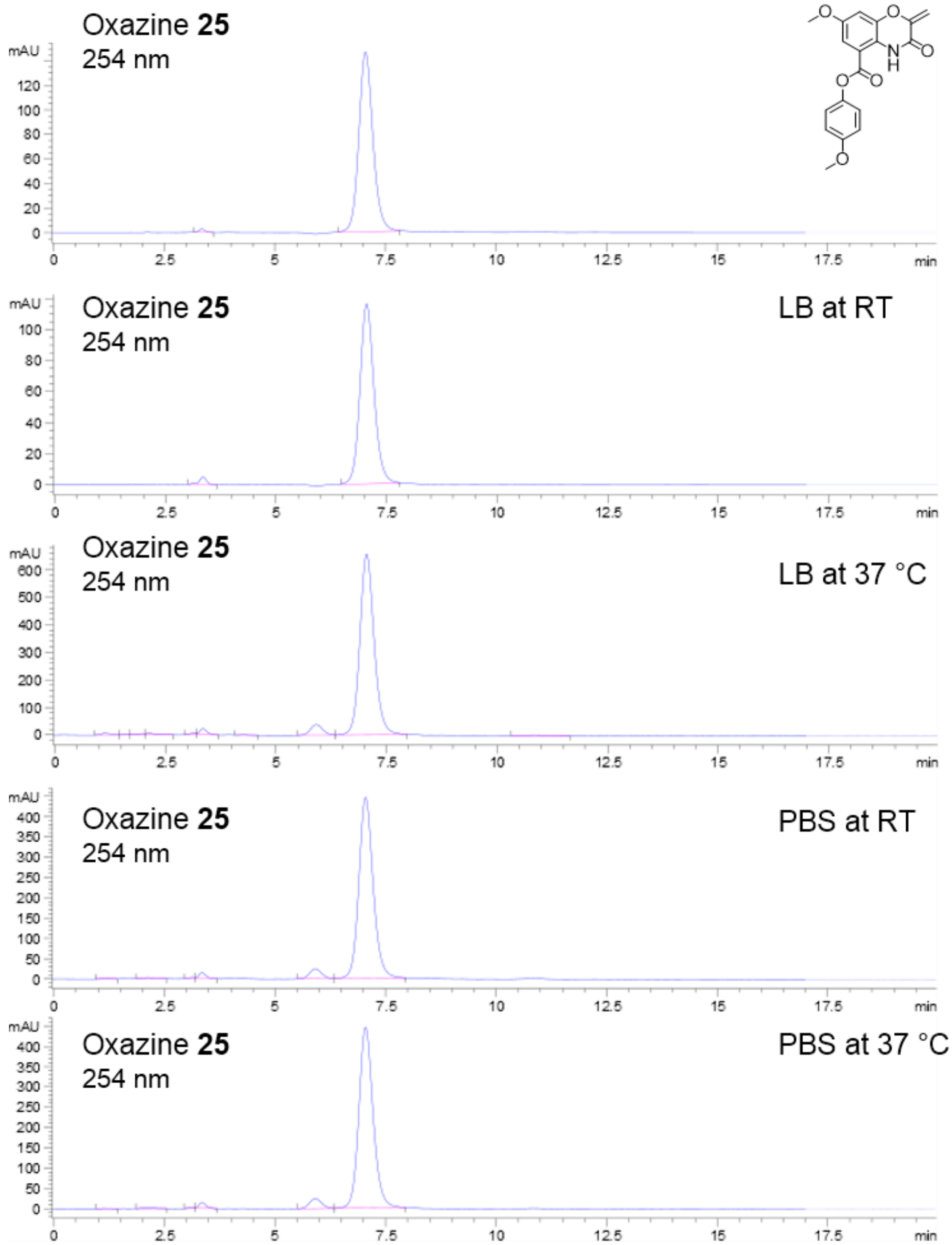

Experimental Details

Methyl-3,5-dimethoxy-2-nitrobenzoate **3**[5]

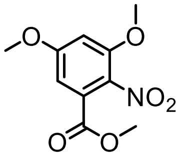

Methyl-3, 5-dimethoxybenzoate **2** (3.0 g, 12.4 mmol) was dissolved in acetic anhydride (20 cm³) and the resulting solution cooled to 0 °C. 70% Nitric acid (1.2 cm³, 18.8 mmol) was introduced dropwise and the subsequent mixture warmed to room temperature and stirred for 15 minutes. The precipitate was filtered, washed with water (3 × 10 cm³) and dried overnight. Recrystallization of the crude material from methanol afforded the title compound as a pale yellow crystalline solid (2.8 g, 94%). δ_H(500 MHz, $CDCl_3$) 3.80 (3H, s, OC*H₃*), 3.87 (3H, s, OC*H₃*), 3.90 (3H, s, OC*H₃*), 6.96 (1H, d, *J* 5.0, ArC*H*), 7.06 (1H, d, *J* 5.0, ArC*H*). All data is in accordance with that of the literature.

Methyl-3-hydroxy-5-methoxy-2-nitrobenzoate **4**

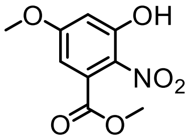

Aluminium chloride (2.2 g, 16.8 mmol) was added portionwise to a solution of the ester **2** (1.0 g, 4.2 mmol) in DCM (20 cm³) at 0 °C over a period of 90 minutes. The resulting blood red solution warmed to room temperature and stirred for a further 60 minutes. The reaction mixture was poured into a slurry of 1*N* HCl (50 cm³) and ice (100 g) and the aqueous phase extracted with ethyl acetate (3 × 50 cm³). The organic layers were combined, washed with brine (50 cm³) and dried over magnesium sulfate. Removal of the solvent *in vacuo* afforded the title compound as a pale yellow solid (860 mg, 89%).δ_H (500 MHz, $CDCl_3$) 3.86 (3H, s, OCH_3), 3.89 (3H, s, OCH_3), 6.52 (1H, s, ArC*H*), 6.55 (1H, s, ArC*H*), 10.89 (1H, s, O*H*); δ_C(125 MHz, CDCl3) 53.6, 56.6, 102.9, 110.2, 125.4, 133.5, 158.2, 165.9, 166.9; *m/z* (ESI-TOF) 228.0603 (100%, MH^+, $C_9H_{10}NO_6$ requires 228.0508).

Methyl 3-(benzyloxy)-5-methoxy-2-nitrobenzoate **5**

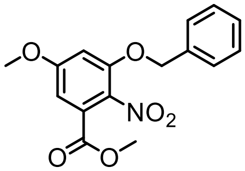

Nitrophenol **4** (500 mg, 2.2 mmol) was added to a suspension of potassium carbonate (1.2 g, 8.8 mmol) and benzyl bromide (1.0 cm³, 8.8 mmol) in a 1:1 mixture of methanol (8 cm³) and dichloromethane (8 cm³). The mixture was heated at reflux for 3 hours before being cooled to room temperature and poured into a 1*N* HCl solution (10 cm³). The aqueous phase was extracted with ethyl acetate (3 × 10 cm³) and the organic layers combined, washed with brine (10 cm³) and dried over magnesium sulfate. Removal of the solvent *in vacuo* yielded a dark orange oil. The oil was triturated with hexane (10 cm³)

and the resultant solid filtered, washed with hexane (10 cm³) and dried to afford the title compound as a pale yellow solid (616 mg, 97%). δ_H(500 MHz, $CDCl_3$) 3.81 (3H, s, OC*H₃*), 3.86 (3H, s, OC*H₃*), 5.14 (2H, s, C*H₂*), 6.70 (1H, d, *J* 2.3, ArC*H*), 6.98 (1H, d, *J* 2.3, ArC*H*), 7.28 – 7.36 (5H, m, ArC*H*); δ_C(125 MHz, $CDCl_3$) 53.4, 56.2, 71.6, 105.3, 106.3, 125.9, 127.3, 128.1, 128.7 (2 × Ar*C*H), 129.0 (2 × Ar*C*H), 135.3, 151.7, 161.1, 164.1; *m/z* (ESI-TOF) 318.0979 (100%, MH^+, $C_{16}H_{16}NO_6$ requires 318.0978).

Methyl-2-amino-3-(benzyloxy)-5-methoxybenzoate **6**

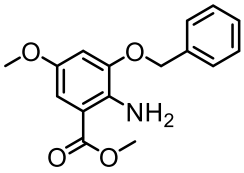

The benzyl protected nitro aromatic (500 mg, 1.6 mmol) **5** was added to a suspension of $SnCl_2.2H_2O$ (1.4 g, 6.4 mmol) in a 3: 1 mixture of ethanol (12 cm³) and 6*N* HCl (4 cm³) and the mixture heated at reflux for 4 hours. Upon cooling to room temperature, the solid hydrochloride salt was filtered, re-dissolved in ethyl acetate and washed with a saturated aqueous solution of Na_2CO_3 (15 cm³). The organic layer was separated and the aqueous phase extracted with extracted with ethyl acetate (3 × 15 cm³). The organic layers were combined, washed with brine (10 cm³) and dried over magnesium sulfate. Removal of the solvent *in vacuo* afforded the title compound as a brown solid that required no further purification (360 mg, 81%). δ_H(500 MHz, $CDCl_3$) 3.74 (3H, s, OC*H₃*), 3.87 (3H, s, OC*H₃*), 5.05 (2H, s, C*H*₂), 5.73 (2H, s, N*H₂*), 6.63 (1H, d, *J* 2.7, ArC*H*), 6.94 (1H, d, *J* 2.7, ArC*H*), 7.32 – 7.44 (5H, m, ArC*H*); δ_C(125 MHz, $CDCl_3$) 51.8, 56.0, 71.0, 103.3, 106.1, 110.0, 127.9 (2 × Ar*C*H), 128.5, 128.9 (2 × Ar*C*H), 136.6, 137.3, 147.5, 149.8, 168.7; *m/z* (ESI-TOF) 288.1235 (100%, MH+, $C_{16}H_{18}NO_4$ requires 288.1236).

Methyl 3-(benzyloxy)-5-methoxy-2-(2-oxopropanamido)benzoate **7**

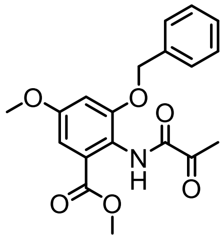

Aniline **6** (500 mg, 1.6 mmol) was dissolved in dichloromethane (5 cm³) and added dropwise to a solution of pyruvoyl chloride[6], pyridine (0.4 cm³, 5 mmol) and dichloromethane (10 cm³) at 0 °C. The mixture was warmed to room temperature and stirred for 15 minutes before being poured into an aqueous solution of 0.1M HCl solution (15 cm³). The organic layer was separated, washed with brine (10 cm³) and dried over magnesium sulfate. Removal of the solvent *in vacuo* afforded a crude orange oil. The oil was dissolved in ethanol (5 cm³) and stored at -20 °C for 30 minutes. The resultant solid was filtered, washed with cold ethanol (5 cm³) and air dried to afford the title compound as a pale yellow solid (234 mg, 65%). δ_H (500 MHz, $CDCl_3$) 2.53 (3H, s, C*H3*), 3.83 (3H, s, OC*H₃*), 3.90 (3H, s, OC*H₃*), 5.14 (2H, s, C*H₂*), 6.75 (1H, d, *J* 2.4, ArC*H*), 7.01 (1H, d, *J* 2.4, ArC*H*), 7.32 – 7.45 (5H, m, ArC*H*), 9.27 (1H, s, N*H*); δ_C (125 MHz, $CDCl_3$) 24.7, 52.8, 56.0,

71.4, 105.0, 106.0, 118.5, 127.2, 127.5 (2 × Ar*C*H), 128.4, 128.9 (2 × Ar*C*H), 136.3, 153.7, 158.2, 158.4, 167.1, 196.8; *m/z* (ESI-TOF) 358.1295 (100%, MH^+, $C_{19}H_{20}NO_6$ requires 358.1291).

Methyl-2-hydroxy-7-methoxy-2-methyl-3-oxo-3,4-dihydro-2*H*-benzo[*b*][1,4]oxazine-5-carboxylate **8**

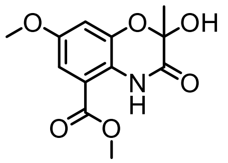

1,4-Cyclohexadiene (400 mg, 0.5 cm³, 5 mmol) was added to a suspension of the α-ketoamide **7** (357 mg, 1 mmol) and Pearlman's catalyst (7 mg, 2 mol% wt) in ethanol (5 cm³). The resulting mixture was heated at 50 ºC for 5 minutes and then cooled to room temperature. Filtration of the suspension through a cotton wool plug afforded the hemi-acetal **8** as a white solid that required no further purification (220 mg, 81%). δ_H (500 MHz, $CDCl_3$) 1.80 (3H, s, C*H*$_3$), 3.55 (1H, s, OH), 3.83 (3H, s, OC*H*$_3$), 3.96 (3H, s, OC*H*$_3$), 6.87 (1H, d, *J* 2.8, ArC*H*), 7.24 (1H, d, *J* ArC*H*), 10.24 (1H, s, N*H*); δ_C (125 MHz, $CDCl_3$) 23.5, 52.8, 56.1, 96.0, 108.9, 110.2, 114.6, 123.2, 143.1, 155.3, 163.7, 167.2; *m/z* (ESI-TOF) 268.0825 (100%, MH+, $C_{12}H_{14}NO_6$ requires 268.0821).

Methyl-7-methoxy-2-methylene-3-oxo-3,4-dihydro-2*H*-benzo[*b*][1,4]oxazine-5-carboxylate **1**[7]

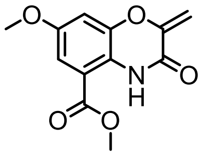

Methanesulfonyl chloride (171 mg, 0.12 cm³, 1.5 mmol) was added dropwise to a solution of hemi-acetal **8** (265 mg, 1 mmol) and *N,N'*-di-*iso*-propylethylamine (258 mg, 0.35 cm³, 2 mmol) in dichloromethane (5 cm³) at 0 °C. The solution was stirred for 90 minutes before being warmed to room temperature and poured into water (5 cm³). The aqueous phase was extracted with ethyl acetate (3 × 5 cm³) and the organic layers combined, washed with brine (10 cm³) and dried over magnesium sulfate. Removal of the solvent *in vacuo* yielded an off white solid that was determined to be >95% pure by LC-MS analysis. To obtain a sample for analytical purposes, the oxazine **1** was purified by flash column chromatography on silica gel using an eluent of 20% ethyl acetate: petroleum ether 40 – 60 ºC (240 mg, 82%). δ_H (500 MHz, $CDCl_3$) 3.78 (3H, s, OC*H*$_3$), 3.92 (3H, s, OC*H*$_3$), 5.07 (1H, dd, *J* 1.5 1.0, 1 × C=C*H*$_2$), 5.62 (1H, d, *J* 1.5, 1 × C=C*H*$_2$), 6.77 (1H, dd, *J* 2.2 1.0, ArC*H*), 7.17 (1H, d, *J* 2.2, ArC*H*), 10.4 (1H, s, N*H*). All data is in accordance with that of the literature.

Methyl-5-methoxy-3-(2-methoxy-2-oxoethoxy)-2-nitrobenzoate **36**

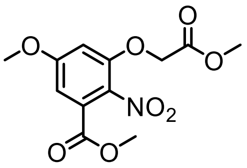

Methyl-2-bromoacetate (0.15 cm³, 0.5 mmol) was added to a suspension of nitrophenol **5** (110 mg, 0.5 mmol) and potassium carbonate (220 mg, 1.5 mmol) in acetone (10 cm³). The mixture was heated at reflux for 3 hours before being cooled to room temperature and filtered. Removal of the solvent *in vacuo* yielded the title compound as an off white solid that required no further purification (120 mg, 76%). δ_H(500 MHz, $CDCl_3$) 3.81 (3H, s, OC*H₃*), 3.87 (3H, s, OC*H₃*), 3.90 (3H, s, OC*H₃*), 4.72 (2H, s, C*H₂*), 6.61 (1H, d, *J* 2.6, ArC*H*), 7.06 (1H, d, *J* 2.6, ArC*H*); δ_C(125 MHz, $CDCl_3$) 52.9, 53.5, 56.4, 66.7, 105.2, 107.3, 126.4, 151.2, 161.2, 162.7, 164.1, 168.3; *m/z* (ESI-TOF) 300.0723 (100%, MH^+, $C_{12}H_{14}NO_8$ requires 300.0719).

Methyl-5-methoxy-3-(1-ethoxy-1-oxopropan-2-yloxy)-2-nitrobenzoate **35**

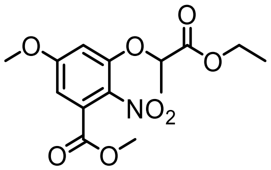

Ethyl-2-bromopropanoate (0.06 cm³, 0.5 mmol) was added to a suspension of nitrophenol **5** (110 mg, 0.5 mmol) and potassium carbonate (220 mg, 1.5 mmol) in acetone (10 cm³). The reaction mixture was heated at reflux for 3 hours before being cooled to room temperature and filtered. Removal of the solvent *in vacuo* yielded the title compound as an off white solid that required no further purification (135 mg, 92%). δ_H(500 MHz, $CDCl_3$) 1.25 (3H, t, *J* 6.8, OCH_2CH_3), 1.62 (3H, d, *J* 6.8, $CHCH_3$), 3.85 (3H, s, OCH_3), 3.89 (3H, s, OCH_3), 4.22 (2H, q, *J* 7.1, OCH_2CH_3), 4.76 (1H, q, *J* 6.8, $CHCH_3$), 6.60 (1H, d, *J* 2.3, ArC*H*), 7.04 (1H, d, *J* 2.3, ArC*H*); δ_C(125 MHz, $CDCl_3$) 14.3, 18.4, 53.4, 56.3, 62.0, 75.0, 105.7, 107.2, 126.1, 136.0, 151.1, 161.0, 164.0, 170.7; *m/z* (ESI-TOF) 314.0874 (100%, MH^+, $C_{13}H_{16}NO_8$ requires 314.0876).

General procedure A for the formation of the oxazine substrates 9 and 10 *via* a platinum(IV) oxide catalysed hydrogenation.

The nitrophenol **5** (0.2 mmol) was added in a single portion to a suspension of platinum(IV) oxide (10% wt) in ethanol (5 cm³). The system was evacuated and backfilled with hydrogen gas five times. Following completion of the final cycle, the mixture was stirred for 4 hours. The suspension was filtered and the solvent removed *in vacuo* to afford the title compound.

Methyl-7-methoxy-3-oxo-3,4-dihydro-2*H*-benzo[*b*][1,4]oxazine-5-carboxylate **9**

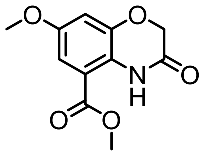

Prepared in accordance to general procedure **A** using nitro-ester **36** (60 mg, 0.2 mmol) and platinum(IV) oxide (6 mg). Removal of the solvent *in vacuo* afforded the title compound as an off white solid (29 mg, 50%). δ_H (500 MHz, $CDCl_3$) 3.82 (3H, s, OC*H₃*), 3.96 (3H, s, OC*H₃*), 4.64 (2H, s, C*H₂*), 6.80 (1H, d, *J* 2.8, ArC*H*), 7.19 (1H, d, *J* 2.8, ArC*H*), 10.26 (1H, s, N*H*); δ_C (125 MHz, $CDCl_3$) 52.8, 56.0, 67.3, 108.3, 109.3, 114.6, 123.5, 145.0, 155.1, 164.4, 167.2; *m/z* (ESI-TOF) 238.0717 (100%, MH^+, $C_{11}H_{12}NO_5$ requires 238.0715).

Methyl-7-methoxy-2-methyl-3-oxo-3,4-dihydro-2*H*-benzo[*b*][1,4]oxazine-5-carboxylate **10**

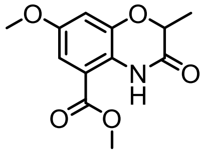

Prepared in accordance to general procedure **A** using nitro-ester **35** (50 mg, 0.2 mmol) and platinum(IV) oxide (5 mg). Removal of the solvent *in vacuo* afforded the title compound as an off white solid (35 mg, 75%). δ_H (500 MHz, $CDCl_3$) 1.60 (3H, d, *J* 6.9, C*H₃*), 3.82 (3H, s, OC*H3*), 3.96 (3H, s, OC*H₃*), 4.67 (1H, q, *J* 6.8, C*H*), 6.81 (1H, d, *J* 2.8, ArC*H*), 7.18 (1H, d, *J* 2.8, ArC*H*), 10.13 (1H, s, N*H*); δ_C (125 MHz, $CDCl_3$) 16.7, 52.8, 56.0, 73.6, 104.8, 108.2, 109.5, 114.3, 123.9, 144.7, 155.0, 166.9; *m/z* (ESI-TOF) 252.0875 (100%, MH^+, $C_{12}H_{14}NO_5$ requires 252.0872).

General Procedure B for the sulphuric acid catalysed addition of an alcohol to the oxazine 1

The alcohol (1 mmol) was added to a solution of oxazine **1** (62 mg, 0.3 mmol) and sulphuric acid (3 drops) in THF (1 cm³) at 0 °C. The resulting solution was stirred for 12 hours and the volatiles removed *in vacuo* to afford the crude product. Purification of the crude material occurred as described in the individual experimental details.

Methyl-2,7-dimethoxy-2-methyl-3-oxo-3,4-dihydro-2H-benzo[b][1,4]oxazine-5-carboxylate **11**

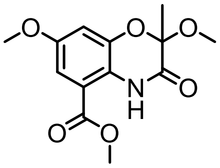

According to general procedure **B** using methanol (0.1 cm³, 3 mmol). Removal of the solvent *in vacuo* afforded the title compound as a white solid that required no further purification (20 mg, 90%). δ_H (500 MHz, $CDCl_3$) 1.76 (3H, s, C*H₃*), 3.34 (3H, s, OC*H₃*), 3.81 (3H, s, OC*H₃*), 3.93 (3H, s, OC*H₃*), 6.85 (1H, d, *J* 2.9, ArC*H*), 7.21 (1H, d, *J* 2.9, ArC*H*), 10.22 (1H, s, N*H*); δ_C (125 MHz, $CDCl_3$) 18.9, 50.2, 52.8, 56.0, 99.3, 108.5, 110.0, 114.3, 123.9, 143.0, 155.0, 162.9, 167.2; *m/z* (ESI-TOF) 281.0901 (100%, MH^+, $C_{13}H_{15}NO_6$ requires 281.0899).

Methyl-7-methoxy-2-methyl-2-(octyloxy)-3-oxo-3,4-dihydro-2H-benzo[b][1,4]oxazine-5-carboxylate **31**

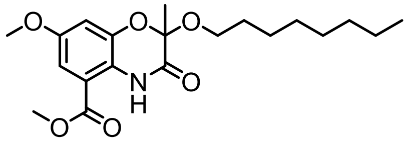

Prepared in accordance with general procedure **B** using 1-octanol (0.2 cm³, 1 mmol). Removal of the solvent *in vacuo* yielded a pale yellow oil. Purification of the crude material by flash column chromatography on silica gel using an eluent of 10% ethyl acetate: *n*-hexanes afforded the title compound as a white solid (32 mg, 35%). δ_H (500 MHz, $CDCl_3$) 0.85 (3H, t, *J* 7.1, CH_2CH_3), 1.06 – 1.18 (8H, m, 4 × CH_2), 1.20 – 1.27 (2H, m, CH_2), 1.37 – 1.44 (2H, m, CH_2), 1.76 (3H, s, CH_3), 3.46 – 3.55 (2H, m, OCH_2), 3.73 (3H, s, OCH_3), 3.86 (3H, s, OCH_3), 6.75 (1H, dd, *J* 2.8 0.6, ArC*H*), 7.12 (d, *J* 2.8, ArC*H*), 10.17 (1H, s, N*H*); δ_C (125 MHz, $CDCl_3$) 14.3, 19.4, 22.9, 26.2, 29.4, 29.7, 32.0, 52.7, 56.0, 62.8, 99.1, 105.4, 108.2, 110.1, 114.1, 124.1, 143.2, 154.9, 163.2, 167.3; *m/z* (ESI-TOF) 380.2076 (100%, MH^+, $C_{20}H_{30}NO_6$ requires 380.2073).

Methyl-2-(but-3-yn-1-yloxy)-7-methoxy-2-methyl-3-oxo-3,4-dihydro-2*H*-benzo[*b*][1,4]oxazine-5-carboxylate **30**

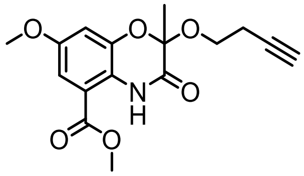

Prepared in accordance with general procedure **B** using but-3-yn-1-ol (0.1 cm³, 1 mmol). Removal of the solvent *in vacuo* afforded an orange oil. Purification of the crude material by flash column chromatography on silica gel afforded the title compound as a white solid (16 mg, 63%). δ_H (500 MHz, $CDCl_3$) 1.78 (3H, s, CH_3), 1.84 (1H, s, C≡C*H*), 2.16 – 2.43 (2H, m, CH_2), 3.70 – 3.76 (2H, m, OCH_2), 3.81 (3H, s, OCH_3), 3.94 (3H, s OC*H3*), 6.84 (1H, d, *J* 2.8, ArC*H*), 7.20 (1H, d, *J* 2.8, ArC*H*), 10.21 (1H, s, N*H*); δ_C (125 MHz, $CDCl_3$) 19.5, 20.0, 52.8, 56.0, 61.0, 69.8, 80.5, 99.1, 108.5, 110.1, 114.3, 123.9, 142.9, 155.0, 162.7, 167.2; *m/z* (ESI-TOF) 320.1137 (100%, MH^+, $C_{16}H_{18}NO_6$ requires 320.1134).

Methyl-2-(hex-3-yn-1-yloxy)-7-methoxy-2-methyl-3-oxo-3,4-dihydro-2*H*-benzo[*b*][1,4]oxazine-5-carboxylate **29**

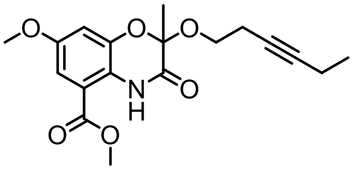

Prepared in accordance with general procedure **B** using hex-3-yn-1-ol (1 cm³, 1 mmol). Removal of the solvent *in vacuo* yielded a crude yellow oil. Purification of the crude material by flash column chromatography on silica gel using an eluent of 20% ethyl acetate: *n*-hexanes afforded the title compound as a white solid (18 mg, 63%). δ_H (500 MHz, $CDCl_3$) 1.04 (3H, t, *J* 7.5, CH_2CH_3), 1.78 (3H, s, CH_3), 2.03 – 2.08 (2H, m, CH_2), 2.21 – 2.35 (2H, m, CH_2), 3.63 – 3.69 (2H, m, OCH_2), 3.81 (3H, s, OCH_3), 3.93 (3H, s, OCH_3), 6.84 (1H, d, *J* 2.8, ArC*H*), 7.19 (1H, d, *J* 2.8, ArC*H*), 10.20 (1H, s, N*H*); δ_C (125

MHz, $CDCl_3$) 12.5, 14.3, 19.4, 20.3, 52.8, 56.0, 61.7, 75.3, 83.4, 99.1, 108.3, 110.0, 114.2, 123.9, 142.9, 154.9, 162.7, 167.2; *m/z* (ESI-TOF) 348.1448 (100%, MH^+, $C_{18}H_{22}NO_6$ requires 348.1147).

Methyl-2-(but-3-en-1-yloxy)-7-methoxy-2-methyl-3-oxo-3, 4-dihydro-2H-benzo[b][1,4]oxazine-5 carboxylate **34**

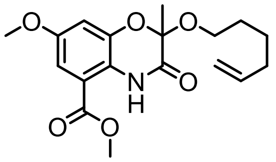

Prepared in accordance to general procedure **B** using 6-hexene-1-ol (0.1 cm³, 1 mmol). Removal of the solvent *in vacuo* yielded a crude yellow oil. Purification of the crude material by flash column chromatography on silica gel using an eluent of 10% ethyl acetate: petroleum ether 40 – 60 ºC afforded the title compound as a white solid (22 mg, 88%). δ_H(500 MHz, $CDCl_3$) 1.76 (7H, s, 2 × CH_2 and CH_3), 2.15 – 2.24 (2H, m, $CH_2CH{=}CH_2$), 3.59 – 3.69 (2H, m, OCH_2), 3.81 (3H, s, OCH_3), 3.93 (3H, s, OCH_3), 4.88 – 4.96 (2H, m, $CH{=}CH_2$), 5.49 – 5.62 (1H, m, $CH{=}CH_2$), 6.81 (1H, d, *J* 2.6, Ar*CH*), 7.19 (1H, d, *J* 2.6, Ar*CH*), 10.19 (1H, s, N*H*); δ_C(125 MHz, $CDCl_3$) 12.5, 14.3, 19.4, 20.3, 52.7, 56.0, 61.7, 75.3, 83.4, 99.0, 108.3, 110.0, 114.2, 123.9, 142.9, 154.9, 162.8, 167.2; *m/z* (ESI-TOF) 350.1607 (100%, MH^+, $C_{15}H_{28}NO_6$ requires 350.1604).

General procedure C for the formation of α-ketoamides 37 - 41 from the aniline 6

The α-ketoacid chloride (2 mmol) was added in a single portion to a solution of aniline (1 mmol) and pyridine (237 mg, 0.3 cm³, 3 mmol) in DCM (10 cm³) at 0 ºC. The resulting mixture was stirred for 1 hour before being quenched through addition of a saturated aqueous solution of $NaHCO_3$ (10 cm³). The organic layer was separated and the aqueous phase extracted with dichloromethane (3 × 10 cm³). The organic layers were combined, washed with brine (10 cm³) and dried over magnesium sulfate. Removal of the solvent *in vacuo* afforded the crude α-ketoamide. Purification of the crude material occurred as described in the individual experimental details.

Methyl-3-(benzyloxy)-5-methoxy-2-(2-oxobutanamido)benzoate **39**

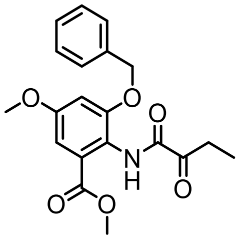

Prepared in accordance with general procedure **C** using 2-oxobutanoyl chloride (238 mg, 2 mmol).[8] Removal of the solvent *in vacuo* yielded a brown oil. Purification of the crude material by recrystallization from ethanol at -20 ºC afforded the title compound as an off white solid (267 mg, 72%). δ_H(500 MHz, $CDCl_3$) 1.16 (3H, t, *J* 7.2, CH_2CH_3), 2.97 (2H, q, *J* 7.2, CH_2CH_3), 3.80 (3H, s, OCH_3), 3.88 (3H, s, OCH_3), 6.72 (1H, d, *J* 2.2, Ar*CH*), 6.99 (1H, d, *J* 2.2, Ar*CH*), 7.32 – 7.42 (5H, m, Ar*CH*), 9.27 (1H, s, N*H*); δ_C(125 MHz, $CDCl_3$) 7.4, 30.6, 52.8, 56.0, 71.4, 105.0, 105.9, 118.6, 127.5, 128.4, 128.9, 153.7, 158.2, 158.3, 167.1, 199.4; *m/z* (ESI-TOF) 372.1449 (100%, MH^+, $C_{20}H_{22}NO_6$ requires 372.1447).

Methyl 3-(benzyloxy)-5-methoxy-2-(2-oxopropanamido)benzoate **40**

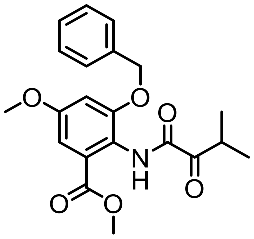

Prepared in accordance with general procedure **C** using 3-methyl-2-oxobutanoyl chloride[8] (268 mg, 2 mmol). Removal of the solvent *in vacuo* yielded an orange oil. Purification of the crude material by recrystallization from ethanol at -20 ºC afforded the title compound as an off white solid (250 mg, 65%). δ_H(500 MHz, $CDCl_3$) 1.62 (5.4H, d, *J* 6.9, 2 × C*H*3 major rotamer), 1.66 (0.6H, d, *J* 6.9, 2 × C*H*3 minor rotamer), 3.63 [0.9H, septet, *J* 6.9, C*H*(CH_3)2 major rotamer], 3.84 (2.7H, s, OC*H*3 major rotamer), 3.85 – 3.92 [0.1H, m, C*H*(CH_3)2 minor rotamer], 3.88 (0.3H, s, OC*H*3 minor rotamer), 3.91 (2.7H, s, OC*H*3 major rotamer), 3.94 (0.3H, s, OC*H*3 minor rotamer), 5.14 (1.8H, s, C*H*2 major rotamer), 5.16 (0.2H, s, C*H*2 minor rotamer), 6.74 (0.1H, d, *J* 2.7, ArC*H* minor rotamer), 6.77 (0.9H, s, d, *J* 2.7, ArC*H* major rotamer), 6.99 (0.1H, d, *J* 2.7, ArC*H* minor rotamer), 7.02 (0.9H, d, *J* 2.7, ArC*H* major rotamer), 7.33 – 7.45 (5H, m, ArC*H* major and minor rotamer); δ_C (125 Hz, $CDCl_3$) 17.9, 34.5, 52.8, 56.0, 71.4, 104.9, 105.9, 118.6, 127.2, 127.6 (2 × Ar*C*H), 128.4, 128.9 (2 × Ar*C*H), 136.2, 153.7, 158.0, 158.3, 167.1, 201.9; *m/z* (ESI-TOF) 384.1605 (100%, MH^+, $C_{21}H_{24}NO_6$ requires 384.1604). Only major rotamer ^{13}C values are reported.

Methyl 3-(benzyloxy)-5-methoxy-2-(4-methyl-2-oxopentanamido)benzoate **37**

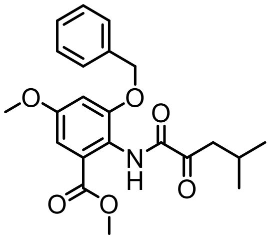

Prepared in accordance with general procedure **C** using 4-methyl-2-oxopentanoyl chloride[8] (296 mg, 2 mmol). Removal of the solvent *in vacuo* yielded brown solid. Purification of the crude material by recrystallization from ethanol at -20 ºC afforded the title compound as a pale yellow solid (252 mg, 60%). δ_H(500 MHz, $CDCl_3$) 0.93 [6H, d, *J* 5.8, 2 × CH(C*H*3)2], 2.15 [1H, nonet, *J* 5.8, CH(C*H*3)2], 2.79 [2H, d, *J* 5.8, C*H*2CH(CH_3)2], 3.79 (3H, s , OC*H*3), 3.86 (3H, s, OC*H*3), 5.09 (2H, s, C*H*2), 6.71 (1H, d, *J* 2.2, ArC*H*), 6.97 (1H, d, *J* 2.2, ArC*H*), 7.28 – 7.40 (5H, m, ArC*H*), 9.23 (1H, s, N*H*); δ_C (125 Hz, $CDCl_3$) 22.5, 24.5, 45.2, 52.5, 55.7, 71.0, 104.7, 105.6, 118.3, 127.0, 127.2 (2 × Ar*C*H), 128.1, 128.6 (2 × Ar*C*H), 136.0, 153.4, 158.1, 166.8, 198.4; *m/z* (ESI-TOF) 400.1764 (100%, MH^+, $C_{22}H_{26}NO_6$ requires 400.1760).

Methyl 3-(benzyloxy)-5-methoxy-2-(2-oxo-2-phenylacetamido)benzoate **38**

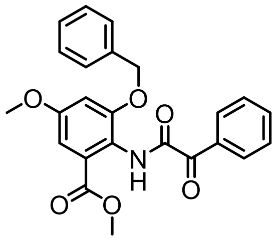

Prepared in accordance with general procedure **C** using 2-oxo-2-phenylacetyl chloride[8] (336 mg, 2 mmol). Removal of the solvent *in vacuo* yielded a brown oil. Purification of the crude material by recrystallization from ethanol at -20 ºC afforded the title compound as a yellow solid (274 mg, 60%). δ_H (500 MHz, $CDCl_3$) 3.83 (3H, s, OC*H₃*), 3.93 (3H, s, OC*H₃*), 5.15 (2H, s, C*H₂*), 6.79 (1H, d, *J* 2.4, ArC*H*), 7.05 (1H, d, *J* 2.4, ArC*H*), 7.33 – 7.43 (5H, m, ArC*H*), 7.47 – 7.48 (2H, m, ArC*H*), 7.59 – 7.62 (1H, m, ArC*H*), 8.23 – 8.24 (2H, m, ArC*H*), 9.49 (1H, s, N*H*); δ_C (125 MHz, $CDCl_3$) 52.9, 56.0, 71.4, 105.1, 106.0, 119.0, 126.9, 127.8 (2 × Ar*C*H), 128.5, 128.7 (2 × Ar*C*H), 128.9 (2 × Ar*C*H), 131.5 (2 × Ar*C*H), 133.5, 134.5, 136.3, 153.8, 158.4, 160.0, 167.2, 188.o; *m/z* (ESI-TOF) 420.1449 (100%, MH^+, $C_{24}H_{22}NO_6$ requires 420.1447).

Methyl-3-(benzyloxy)-5-methoxy-2-(2-oxo-2-(p-tolyl)acetamido)benzoate **41**

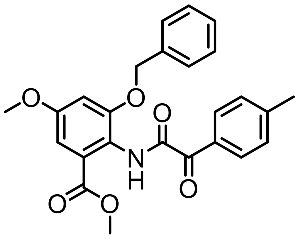

Prepared in accordance with general procedure **C** using 2-oxo-2-(*p*-tolyl)acetyl chloride[8] (364 mg, 2 mmol). Removal of the solvent *in vacuo* yielded a brown oil. Purification of the crude material by recrystallization from ethanol at -20 ºC afforded the title compound as a yellow solid (210 mg, 73%). δ_H (500 MHz, $CDCl_3$) 2.42 (3H, s, ArC*H₃*), 3.83 (3H, s, OC*H₃*), 3.92 (3H, s, OC*H₃*), 5.15 (2H, s, C*H₂*), 6.78 (1H, d, *J* 2.7, ArC*H*), 7.04 (1H, d, *J* 2.7, ArC*H*), 7.23 (2H, d, *J* 8.0, ArC*H*), 7.33 – 7.39 (3H, m, ArC*H*), 7.45 – 7.47 (2H, m, ArC*H*), 8.18 (2H, d, *J* 8.0, ArC*H*), 9.47 (1H, s, N*H*); δ_C (125 MHz, $CDCl_3$) 22.1, 52.9, 56.0, 71.4, 105.1, 106.0, 119.0, 126.9, 127.8 (2 × Ar*C*H), 128.4, 128.9 (2 × Ar*C*H), 129.5 (2 × Ar*C*H), 131.0, 131.6 (2 × Ar*C*H), 136.3, 145.7, 153.8, 158.3, 160.3, 167.2, 187.4; *m/z* (ESI-TOF) 434.1605 (100%, MH+, $C_{25}H_{24}NO_6$ requires 434.1604).

General procedure D for the formation of the oxazines 13 - 15 from the α-keto amides 37 , 39 and 40

The α-keto amide **38**, **40** or **41** (1 mmol) was added to a suspension of 20% $Pd(OH)_2/C$ (2 mol %) and 1,4-cyclohexadiene (400 mg, 0.5 cm^3, 5 mmol) in ethanol (5 cm^3). The reaction mixture was heated at reflux for 5 minutes before being cooled to room temperature. The suspension was filtered through a cotton wool plug and the solvent removed *in vacuo* to afford the hemi-acetal intermediate. The hemi-acetal intermediate was re-dissolved in THF (5 cm^3) and added to a suspension of *p*-toluenesulfonic acid (2 equivalents) in THF (5 cm^3). The mixture was heated at reflux for 90 minutes before being cooled

to room temperature. Removal of the volatiles *in vacuo* yielded the crude oxazine. Purification of the crude material occurred as described in the individual experimental details.

Methyl-(*Z*)-2-ethylidene-7-methoxy-3-oxo-3,4-dihydro-2*H*-benzo[*b*][1,4]oxazine-5-carboxylate **14**

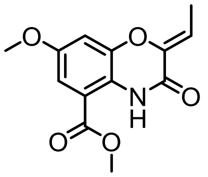

Prepared in accordance with general procedure **D** using α-keto amide **39** (371 mg, 1 mmol), 20% $Pd(OH)_2/C$ (7 mg, 2 mol %) and *p*-toluenesulfonic acid (260 mg, 1.2 mmol). Removal of the solvent *in vacuo* yielded a yellow oil as a 9:1 mixture of isomers. Purification and isolation of the major isomer by flash column chromatography on silica gel using an eluent of 5% ethyl acetate: *n*-hexanes afforded the title compound as a white solid (120 mg, 45%). δ_H (600 MHz, $CDCl_3$) 1.87 (3H, d, *J* 7.3, C*H3*), 3.84 (3H, s, OC*H3*), 3.96 (3H, s, OC*H3*), 6.11 (1H, q, *J* 7.3, C=C*H*), 6.85 (1H, d, *J* 2.8, ArC*H*), 7.18 (1H, d, *J* 2.8, ArC*H*), 10.28 (1H, s, N*H*); δ_C (150 MHz, d_6-Acetone) 9.2, 52.1, 55.3, 107.2, 108.3, 111.0, 113.7, 121.9, 142.2, 142.8, 154.7, 155.2, 167.0; *m/z* (ESI-TOF) 264.0875 (100%, MH^+, $C_{13}H_{14}NO_5$ requires 264.0872).

Methyl-7-methoxy-3-oxo-2-(propan-2-ylidene)-3,4-dihydro-2*H*-benzo[*b*][1,4]oxazine-5-carboxylate **15**

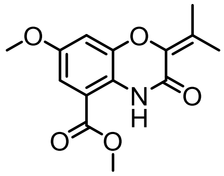

Prepared in accordance with general procedure **D** using α-keto amide **40** (385 mg, 1 mmol), 20% $Pd(OH)_2/C$ (8 mg, 2 mol %) and *p*-toluenesulfonic acid (190 mg, 1 mmol). Removal of the solvent *in vacuo* yielded a brown oil. Purification of the crude material by flash column chromatography on silica gel using an eluent of 10% ethyl acetate: *n*-hexanes afforded the title compound as a white solid (83 mg, 30%). δ_H (600 MHz, d_6-Acetone) 1.94 (3H, s, C*H3*), 2.23 (3H, s, C*H3*), 3.81 (3H, s, OC*H3*), 3.93 (3H, s, OC*H3*), 6.93 (1H, d, *J* 2.8, ArC*H*), 7.12 (1H, d, *J* 2.8, ArC*H*), 9.87 (1H, s, N*H*); δ_C (150 MHz, d_6-Acetone) 18.5, 19.1, 52.0, 55.3, 107.2, 107.7, 113.2, 123.0, 128.5, 135.7, 144.1, 154.6, 157.3, 166.9; *m/z* (ESI-TOF) 278.1030 (100%, MH^+, $C_{14}H_{16}NO_5$ requires 278.1028).

Methyl-(*Z*)-7-methoxy-2-(2-methylpropylidene)-3-oxo-3,4-dihydro-2*H*-benzo[b][1,4]oxazine-5-carboxylate **13**

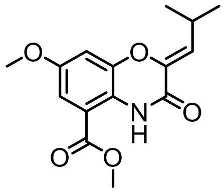

Prepared in accordance with general predure **D** using α-keto amide **37** (399 mg, 1 mmol), 20% $Pd(OH)_2/C$ (8 mg, 2 mol %) and *p*-toluenesulfonic acid (258 mg, 1.6 mmol). Removal of the solvent *in vacuo* yielded a crude orange oil. Purification of the crude material by flash column chromatography on silica gel using an eluent of 10% ethyl acetate: *n*-hexanes

afforded the title compound as a white solid (99 mg, 34%). δ_H (600 MHz, d_6-DMSO) 1.07 [6H, d, *J* 7.3, CH(C*H*$_3$)$_2$], 2.92 – 2.99 [1H, m, C*H*(CH$_3$)$_2$], 3.77 (3H, s, OC*H$_3$*), 3.89 (3H, s, OC*H$_3$*), 5.83 [1H, d, *J* 9.6, C=C*H*CH(CH$_3$)$_2$], 7.05 (1H, d, *J* 2.8, ArC*H*), 7.09 (1H, d, *J* 2.8, ArC*H*), 10.09 (1H, s, N*H*); δ_C (150 MHz, d_6-Acetone) 21.5, 24.2, 52.4, 55.3, 107.2, 108.4, 113.7, 121.9, 122.8, 140.0, 142.8, 154.7, 155.4, 167.0; *m/z* (ESI-TOF) 292.1188 (100%, MH$^+$, $C_{15}H_{18}NO_5$ requires 292.1185).

Methyl-2-hydroxy-7-methoxy-3-oxo-2-phenyl-3,4-dihydro-2*H*-benzo[*b*][1,4]oxazine-5-carboxylate **28** and methyl-7-methoxy-3-oxo-2-phenyl-3,4-dihydro-2*H*-benzo[*b*][1,4]oxazine-5-carboxylate **12**

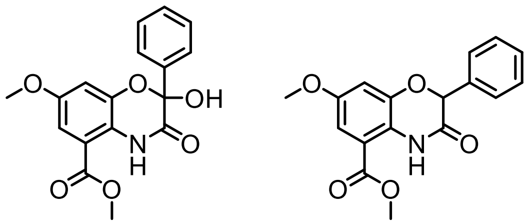

1,4-Cyclohexadiene (400 mg, 0.5 cm³, 5 mmol) was added to a suspension of the α-ketoamide **38** (419 mg, 1 mmol) and 20% Pd(OH)$_2$/C (8 mg, 2 mol %) and the resulting mixture heated at 50 ºC for 2 hours. Upon cooling to room temperature, TLC analysis revealed the presence of two compounds, the hemi-acetal **26** (R_f 0.15, 20% EtOAc: *n*-hexanes) and the fully hydrogenated phenyl oxazine **12** (R_f 0.3, 20% EtOAc: *n*-hexanes). Removal of the solvent *in vacuo* afforded a 1:1 mixture of the two compounds as determined by analysis of the crude ^{1}H NMR spectroscopy data. Purification and isolation of both compounds by flash column chromatography on silica gel using an eluent of 20% ethyl acetate: *n*-hexanes afforded the hemi-acetal **26** as an orange solid (118 mg, 36%) and the phenyl oxazine **12** (98 mg, 31%) as a yellow solid. Hemi-acetal **26**: δ_H (500 MHz, CDCl$_3$) 3.70 (3H, s, OCH3), 3.83 (3H, s, OCH3), 5.25 (1H, s, OH), 6.69 (1H, d, *J* 3.0, ArC*H*), 7.06 (1H, d, *J* 3.0, ArC*H*), 7.25 – 7.33 (3H, m, ArC*H*), 7.43 – 7.45 (2H, m, ArC*H*), 11.66 (1H, s, N*H*); δ_C (125 MHz, CDCl$_3$) 53.0, 55.9, 75.3, 109.4, 110.7, 120.8, 121.3, 127.1, 129.2, 129.3, 138.9, 151.5, 158.0, 168.4, 172.5; *m/z* (ESI-TOF) 330.0980 (100%, MH$^+$, $C_{17}H_{16}NO_6$ requires 330.0978). Phenyl Oxazine **12**: δ_H (500 MHz, CDCl$_3$) 3.95 (1.5H, s, OC*H$_3$*, rotamer A), 3.98 (1.5H, s, OC*H3*, rotamer B), 4.05 (1.5H, s, OC*H3*, rotamer A), 4.08 (1.5H, s, OC*H3*, rotamer B), 6.94 (0.5H, d, *J* 2.8, C*H* rotamer A), 7.32 (0.5H, d, *J* 2.8, C*H*, rotamer B), 7.39 (0.5H, d, *J* 2.5, ArC*H*, rotamer A), 7.49 – 7.54 (1.5H, m, ArC*H*, rotamer A and B), 7.58 – 7.61 (1H, m, ArC*H*, rotamer A and B), 7.69 – 7.72 (0.5H, m, ArC*H*, rotamer A), 7.78 (0.5H, d, *J* 2.3, ArC*H*, rotamer B), 8.29 – 8.41 (1H, m, rotamer A and B), 8.65 – 8.67 (1H, m, rotamer A and B); δ_C (125 MHz, CDCl$_3$) 52.9 (rotamer A), 53.1 (rotamer B), 56.5 (rotamer A), 56.6 (rotamer B), 100.2 (rotamer A), 103.2 (rotamer B), 114.3 (rotamer A), 117.5 (rotamer B), 124.4 (rotamer A), 124.7 (rotamer B), 128.6 (2 × rotamer A), 128.9 (2 × rotamer B), 129.7 (2 × rotamer A), 131.4 (2 × rotamer B), 131.5 (rotamer A), 132.5 (rotamer B), 133.8 (rotamer A), 134.4 (rotamer B), 134.6 (rotamer A), 135.2 (rotamer B) 147.6 (rotamer A), 147.6 (rotamer B), 148.5 (rotamer B), 151.8 (rotamer A), 152.4 (rotamer B), 160.2 (rotamer A), 161.4 (rotamer B), 165.1 (rotamer A), 166.4 (rotamer B), 180.0 (rotamer A), 180.1 (rotamer B); *m/z* (ESI-TOF) 314.1032 (100%, MH$^+$, $C_{17}H_{16}NO_5$ requires 314.1028).

General procedure E for the palladium catalysed Heck reaction between the oxazine 1 and an aryl bromide

N, *N*-diisopropylethylamine (5 µl, 3 mg, 30 µmol) was added to a suspension of Palladium acetate (1 mg, 4 µmol), triphenylphosphine (2 mg, 8 µmol), oxazine **1** (5 mg, 20 µmol) and the aryl bromide (20 µmol) in toluene (3 cm^3). The mixture was heated at reflux for 2 hours and then cooled to room temperature. The residue was purified as described in the individual experimental details.

Methyl 2-benzylidene-7-methoxy-3-oxo-3, 4-dihydro-2*H*-benzo[b][1,4]oxazine-5-carboxylate **16**

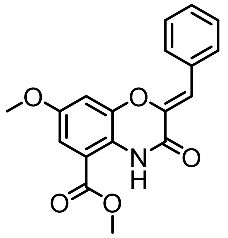

Prepared in accordance with general procedure **E** using bromobenzene (2 µl, 3 mg, 20 µmol). The residue was purified by flash column chromatography on silica gel using an eluent of 20% ethyl acetate: *n*-hexane to afford the title compound as a pale yellow solid (4 mg, 62%). δ_H (500 MHz, d_6-DMSO) 3.84 (3H, s, OC*H3*), 3.93 (3H, s, OC*H3*), 6.83 – 6.85 (1H, m, ArC*H*), 7.16 (1H, d, *J* 2.8, ArC*H*), 7.35 (1H, d, *J* 2.8, ArC*H*), 7.37 – 7.40 (1H, m, ArC*H*), 7.47 (2H, t, *J* 7.5, ArC*H*), 7.93 – 7.94 (1H, m, ArC*H*); δ_C (125 MHz, d_6-DMSO) 53.6, 56.7, 108.2, 109.8, 112.5, 114.8, 121.6, 129.3, 129.5 (2 × Ar*C*H), 130.8 (2 × Ar*C*H), 133.5, 141.4, 142.6, 155.1, 156.0, 167.1; *m/z* (ESI-TOF) 326.1029 (100%, MH^+, $C_{18}H_{16}NO_5$ requires 326.1028).

Methyl-(*Z*)-7-methoxy-2-(4-nitrobenzylidene)-3-oxo-3,4-dihydro-2*H*-benzo[*b*][1,4]oxazine-5-carboxylate **17**

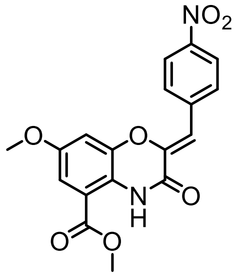

Prepared in accordance with general procedure **E** using 4-nitrobromobenzene (3 mg, 20 µmol). The residue was purified by flash column chromatography on silica gel using an eluent of 20% ethyl acetate: *n*-hexane to afford the title compound as a bright yellow solid (4 mg, 62%). δ_H (500 MHz, $CDCl_3$) 3.83 (3H, s, OCH_3), 3.94 (3H, s, OCH_3), 6.94 (1H, s, C=C*H*), 7.20 (1H, d, *J* 2.3, ArC*H*), 7.31 – 7.34 (1H, m, ArC*H*), 7.39 – 7.42 (2H, m, ArC*H*), 7.80 – 7.82 (2H, m, ArC*H*), 10.41 (1H, s, N*H*); δ_C (125 MHz, $CDCl_3$) 52.6, 55.9, 107.8, 108.9, 113.2, 113.8, 121.9, 128.5 (2 × Ar*C*H), 128.6, 130.1 (2 × Ar*C*H), 133.2, 140.6, 142.4, 154.7, 156.4, 166.8; *m/z* (ESI-TOF) 371.0881 (100%, MH^+, $C_{15}H_{18}N_2O_7$ requires 371.0879).

Methyl-7-methoxy-4-methyl-2-methylene-3-oxo-3,4-dihydro-2*H*-benzo[*b*][1,4]oxazine-5-carboxylate **18**

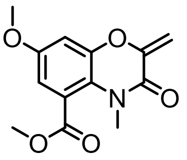

Iodomethane (6 µl, 0.1 mmol) was added in a single portion to a suspension of oxazine **1** (15 mg, 0.06 mmol) and potassium carbonate (14 mg, 0.1 mmol) in DMF (1 cm³). The mixture was stirred vigourously overnight before being poured into a solution of 1*N* HCl (10 cm³) and ethyl acetate (10 cm³). The organic layer was extracted, washed with brine (5 × 10 cm³) and dried over magnesium sulfate. Removal of the solvent yielded a yellow oil. Purification of the crude material by flash column chromatography on silica gel using an eluent of 10% ethyl acetate: *n*-hexane afforded the title compound as a colourless oil (10 mg, 66%). δ_H(500 MHz, $CDCl_3$) 3.28 (3H, s, N*CH₃*), 3.83 (3H, s, OC*H₃*), 3.95 (3H, s, OC*H₃*), 5.14 (1H, d, *J* 1.8, 1 × C=C*H₂*), 5.64 (1H, d, *J* 1.8, 1 × C=C*H₂*), 6.75 (1H, d, *J* 2.9, ArC*H*), 6.84 (1H, d, *J* 2.9, ArC*H*); δ_C(125 MHz, $CDCl_3$) 34.7, 53.1, 56.1, 100.7, 105.2, 109.6, 122.2, 145.5, 148.3, 155.8, 158.6, 168.0, 191.1; *m/z* (ESI-TOF) 264.0875 (100%, MH^+, $C_{13}H_{14}NO_5$ requires 264.0872).

7-methoxy-2-methylene-3-oxo-3,4-dihydro-2*H*-benzo[*b*][1,4]oxazine-5-carboxylic acid **19**

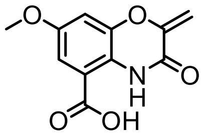

Lithium hydroxide monohydrate (43 mg, 1 mmol) was added in a single portion to a solution of oxazine **1** (63 mg, 0.5 mmol) in THF (2 cm³), MeOH (2 cm³) and water (2 cm³). The mixture was stirred for 90 minutes before being acidified to pH 1 through addition of an aqueous solution of 1*N* hydrochloric acid (3 cm³). The precipitated solid was filtered, washed with cold methanol and air dried to afford the title compound as a white solid (23 mg, 36%). δH (500 MHz, *d6*-DMSO) 3.77 (3H, s, OC*H3*), 5.14 (1H, d, *J* 2.4, 1 × C=C*H2*), 5.48 (1H, d, *J* 2.4, 1 × C=C*H2*), 7.01 (1H, d, *J* 2.4, ArC*H*), 7.14 (1H, d, *J* 2.4, ArC*H*), 10.55 (1H, s, CO2*H*)**;** δC (125 MHz, *d6*-DMSO) 56.5, 98.3, 99.3, 107.6, 109.6, 115.6, 121.8, 142.7, 155.0, 155.1, 169.0; *m/z* (ESI-TOF) 235.0562 (100%, MH^+, $C_{11}H_{10}NO_5$ requires 235.0559).

General Procedure F for the condensation of either an alcohol or an amine with the carboxylic acid 19

Oxalyl chloride (8 µl, 0.1 mmol) was added to a suspension of the acid **19** (10 mg, 0.04 mmol) and DMF (1 drop) in DCM (5 cm³). The mixture was heated at reflux for 4 hours before being cooled to room temperature. Removal of the volatiles *in vacuo* afforded the acid chloride as a yellow solid. The solid was dissolved in DCM (2 cm³) and added in a single portion to a solution of the nucleophile (0.1 mmol) and pyridine (16 mg, 20 µl, 0.2 mmol) in DCM (5 cm³) at 0 °C. The mixture was warmed to room temperature and stirred for 90 minutes before being poured into water (10 cm³). The aqueous phase was extracted with ethyl acetate (3 × 15 cm³) and the organic layers combined, washed with brine (10 cm³) and dried over magnesium sulphate. Removal of the solvent *in vacuo* afforded the crude ester or amide. Purification of the crude material occurred as described in the individual experimental procedure.

Phenyl-7-methoxy-2-methylene-3-oxo-3,4-dihydro-2*H*-benzo[*b*][1,4]oxazine-5-carboxylate **21**

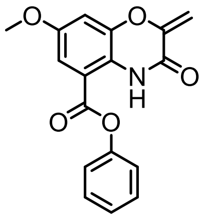

Prepared in accordance with general procedure **F** using phenol (9 mg, 0.1 mmol). Removal of the solvent *in vacuo* yielded a crude yellow oil. Purification of the crude material by flash column chromatography on silica gel using an eluent of 15% ethyl acetate: *n*-hexanes afforded the title compound as a white solid (9 mg, 75%). δ_H (600 MHz, d_6-Acetone) 3.88 (3H, s, OCH_3), 5.09 (1H, d, *J* 2.1, 1 × C=CH_2), 5.52 (1H, d, *J* 2.1, 1 × C=CH_2), 7.01 (1H, d, *J* 2.7, ArC*H*), 7.32 – 7.36 (2H, m, ArC*H*), 7.47 – 7.52 (4H, m, ArC*H*), 10.13 (1H, s, N*H*); δ_C (150 MHz, d_6-Acetone) 55.5, 98.2, 107.9, 109.1, 113.4, 121.8 (2 × Ar*C*H), 122.4, 126.3, 129.5 (2 × Ar*C*H), 142.6, 148.0, 150.5, 154.6, 154.9, 165.2; *m/z* (ESI-TOF) 312.0874 (100%, MH^+, $C_{17}H_{14}NO_5$ requires 312.0872).

2,4,5-Trichlorophenyl-7-methoxy-2-methylene-3-oxo-3,4-dihydro-2*H*-benzo[*b*][1,4]oxazine-5-carboxylate **22**

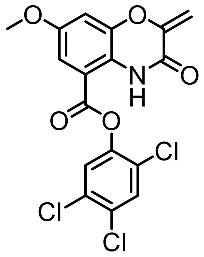

Prepared in accordance with general procedure **F** using 2, 4, 5-trichlorophenol (20 mg, 0.1 mmol). Removal of the solvent *in vacuo* yielded a crude yellow oil. Purification of the crude material by flash column chromatography on silica gel using an eluent of 10% ethyl acetate: *n*-hexanes afforded the title compound as a white solid (8 mg, 50%). δ_H (600 MHz, d_6-Acetone) 3.83 (3H, s, OCH_3), 5.11 (1H, d, *J* 1.7, 1 × C=CH_2), 5.53 (1H, d, *J* 1.7, 1 × C=CH_2), 7.06 (1H, d, *J* 2.7, ArC*H*), 7.48 (1H, d, *J* 2.7, ArC*H*), 7.85 (1H, s, ArC*H*), 7.92 (1H, s, ArC*H*), 9.89 (1H, s, N*H*); δ_C (150 MHz, d_6-Acetone) 55.6, 98.4, 108.5, 109.2, 112.1, 122.7, 125.2, 125.9, 126.3, 130.6, 131.2, 142.7, 145.7, 147.8, 154.6. 155.0, 163.8; *m/z* (ESI-TOF) 413.9705 (100%, MH+, $C_{17}H_{11}Cl_3NO_5$ requires 413.9703).

4-Methoxyphenyl-7-methoxy-2-methylene-3-oxo-3,4-dihydro-2*H*-benzo[*b*][1,4]oxazine-5-carboxylate **25**

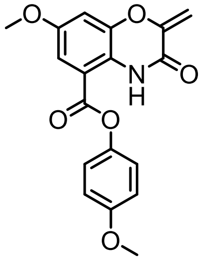

Prepared in accordance with general procedure **F** using 4-methoxyphenol (12 mg, 0.1 mmol). Removal of the solvent *in vacuo* yielded a yellow oil. Purification of the crude material by flash column chromatography on silica gel using an eluent

of 20% ethyl acetate: *n*-hexanes afforded the title compound as a white solid (6 mg, 52%). δ_H (600 MHz, d_6-Acetone) 3.82 (3H, s, OC*H₃*), 3.88 (3H s, OC*H₃*), 5.10 (1H, d, *J* 2.1, 1 × C=C*H₂*), 5.53 (1H, d, *J* 2.1, 1 × C=C*H₂*), 7.01 – 7.04 (3H, m, ArC*H*), 7.23 – 7.26 [2H, $(AX)_2$, ArC*H*], 7.45 (1H, d, *J* 2.8, ArC*H*), 10.15 (1H, s, N*H*); δ_C (150 MHz, $CDCl_3$) 55.6, 55.9, 99.6, 108.3, 108.8, 114.6 (2 × Ar*C*H), 114.8, 116.0, 122.3 (2 × Ar*C*H), 142.7, 143.4, 147.5, 149.4, 154.8, 157.7, 165.6; *m/z* (ESI-TOF) 342.0980 (100%, MH^+, $C_{18}H_{16}NO_6$ requires 342.0978).

7-Methoxy-2-methylene-3-oxo-3,4-dihydro-2*H*-benzo[b][1,4]oxazine-5-carboxamide **20**

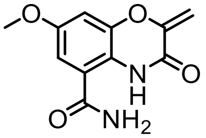

Prepared in accordance with general procedure **F** using ammonium hydroxide (4 µl, 0.1 mmol). Removal of the solvent *in vacuo* afforded an orange solid. The solid was triturated with cold acetone to afford the title compound as an off white solid (2 mg, 25%). δ_H (600 MHz, d_6-DMSO) 3.84 (3H, s, OC*H3*), 5.18 (1H, d, *J* 2.2, 1 × C=C*H₂*), 5.50 (1H, d, *J* 2.2, 1 × C=C*H₂*), 6.99 (1H, d, *J* 2.2, ArC*H*), 7.26 (1H, d, *J* 2.3, ArC*H*), 7.87 (1H, s, 1 × N*H₂*), 8.37 (1H, s, 1 × N*H₂*), 11.49 (1H, s, N*H*); δ_C(150 MHz, d_6-DMSO) 56.6, 98.8, 105.6, 108.2, 115.3, 121.0, 127.9, 142.7, 148.5, 154.9, 170.0; *m/z* (ESI-TOF) 235.0720 (100%, MH+, $C_{11}H_{11}N_2O_4$ requires 235.0719).

7-Methoxy-*N*-(4-methoxyphenyl)-2-methylene-3-oxo-3,4-dihydro-2*H*-benzo[*b*][1,4]oxazine-5-carboxamide **24**

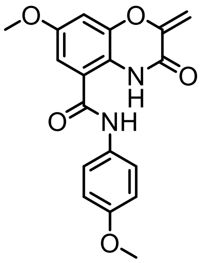

Prepared in accordance with general procedure **F** using 4-methoxyaniline (12 mg, 0.1 mmol). Removal of the solvent *in vacuo* yielded a crude orange oil. Purification of the crude material by flash column chromatography on silica gel using an eluent of 30% ethyl acetate: *n*-hexanes afforded the title compound as an off white solid (8 mg, 63%). δ_H (500 MHz, $CDCl_3$) 3.85 (3H, s, OC*H3*), 3.86 (3H, s, OC*H3*), 5.10 (1H, d, *J* 2.1, 1 × C=C*H₂*), 5.65 (1H, d, *J* 2.1, 1 × C=C*H₂*), 6.78 (1H, d, *J* 2.6, ArC*H*), 6.82 (1H, d, *J* 2.6, ArC*H*), 6.93 – 6.97 [2H, $(AX)_2$, ArC*H*], 7.47 – 7.52 [2H, $(AX)_2$, ArC*H*], 7.77 (1H, s, N*H*), 10.51 (1H, s, N*H*); δ_C (150 MHz, d_6-DMSO) 56.0, 56.6, 95.0, 101.9, 114.9 (2 × Ar*C*H), 117.5, 120.0, 129.6 (2 × Ar*C*H), 133.6, 141.4, 148.1, 155.5, 156.3, 159.1, 160.0, 163.3; *m/z* (ESI-TOF) 341.1140 (100%, MH+, $C_{18}H_{17}N_2O_5$ requires 341.1137).

7-Methoxy-*N*-phenyl-2-methylene-3-oxo-3,4-dihydro-2*H*-benzo[*b*][1,4]oxazine-5-carboxamide **23**

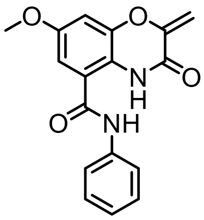

Prepared in accordance with general procedure **F** using aniline (10 µl, 0.1 mmol). Removal of the solvent *in vacuo* yielded a crude orange oil. Purification of the crude material by flash column chromatography on silica gel using an eluent of 30% ethyl acetate: *n*-hexanes afforded the title compound as a white solid (7 mg, 58%). δ_H (500 MHz, $CDCl_3$) 3.76 (3H, s, OC*H3*), 5.01 (1H, d, *J* 2.3, 1 × C=C*H*$_2$), 5.56 (1H, d, *J* 2.1, 1 × C=C*H*$_2$), 6.70 (1H, d, *J* 2.1, 1 × C=C*H*$_2$), 7.13 (1H, app. t, *J* 7.3, ArC*H*), 7.33 (2H, app. t, *J* 7.3, ArC*H*), 7.50 (2H, d, *J* 7.3, ArC*H*), 7.78 (1H, s, N*H*), 10.36 (1H, s, N*H*); δ_C (125 MHz, $CDCl_3$) 56.3, 99.5, 104.9, 105.2, 106.5, 121.0, 125.7, 129.5, 137.1, 143.6, 148.1, 149.5, 155.3, 155.6, 165.3; *m/z* (ESI-TOF) 311.1036 (100%, MH^+, $C_{17}H_{15}N_2O_4$ requires 311.1032).

N-Heptyl-7-methoxy-2-methylene-3-oxo-3, 4-dihydro-2H-benzo[b][1,4]oxazine-5-carboxamide **27**

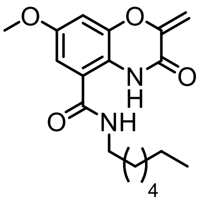

Prepared in accordance with general procedure **F** using 1-aminoheptane (8 mg, 100 µl, 0.8 mmol). Removal of the solvent *in vacuo* yielded a yellow oil. Purification of the crude material by flash column chromatography on silica gel using an eluent of 15% ethyl acetate: *n*-hexanes afforded the title compound as a white solid (8 mg, 75%). δ_H(500 MHz, $CDCl_3$) 0.89 (3H, t, *J* 6.8, C*H*$_3$), 1.26 – 1.36 (8H, m, 4 × C*H*$_2$), 1.58 – 1.63 (2H, m, C*H*$_2$), 3.41 (2H, q, *J* 7.1, C*H*$_2$N), 3.80 (3H, s, OC*H*$_3$), 5.05 (1H, d, *J* 1.6, 1 × C=C*H*$_2$), 5.61 (1H, d, *J* 1.6, 1 × C=C*H*$_2$), 6.20 (1H, s, *N*H), 6.64 (1H, d, *J* 2.6, ArC*H*), 6.70 (1H, d, *J* 2.6, ArC*H*), 10.65 (1H, s, N*H*); δ_C(500 MHz, $CDCl_3$) 14.3, 22.8, 27.2, 29.2, 29.7, 32.0, 40.3, 56.2, 95.0, 99.2, 104.6, 106.4, 118.7, 120.4, 143.4, 148.2, 155.2, 167.0; *m/z* (ESI-TOF) 291.1348 (100%, MH^+, $C_{15}H_{19}N_2O_4$ requires 291.1345).

Methyl-2-acetoxy-7-methoxy-2-methyl-3-oxo-3,4-dihydro-2H-benzo[b][1,4]oxazine-5-carboxylate **26**

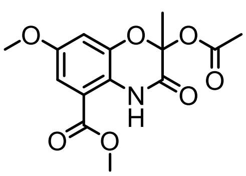

Triethylamine (12 µl, 80 µmol) was added dropwise to a solution of hemi-acetal **8** (10 mg, 400 µmol), acetyl chloride (6 µl, 800 µmol) and DMAP (1 mg, 8 µmol) in DCM (2 cm³) at -10 °C. The reaction mixture was stirred for 1 hour before the volatiles were removed *in vacuo*. The crude residue was purified by flash column chromatography on silica gel using

an eluent of 15% ethyl acetate: *n*-hexanes to afford the title compound as a white solid (7 mg, 50%). δ_H (500 MHz, $CDCl_3$) 1.88 (3H, s, C*H₃*), 2.08 (3H, s, C*H₃*), 3.79 (3H, s, OC*H₃*), 3.93 (3H, s, OC*H₃*), 6.73 (1H, d, *J* 3.0, ArC*H*), 7.18 (1H, d, *J* 3.0, ArC*H*), 10.39 (1H, s, N*H*); δ_C (500 MHz, $CDCl_3$) 21.2, 24.2, 52.8, 56.0, 98.0, 108.4, 108.5, 114.1, 122.5, 143.0, 155.0, 162.2, 167.3, 169.5; *m/z* (ESI-TOF) 310.0930 (100%, MH^+, $C_{14}H_{16}NO_7$ requires 310.0927).

Methyl-4-benzoyl-2-(benzoyloxy)-7-methoxy-2-methyl-3-oxo-3,4-dihydro-2H-benzo[b][1,4]oxazine-5-carboxylate **32**

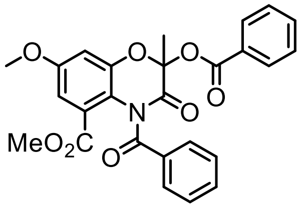

Benzoyl chloride (20 µl, 0.16 mmol) was added to a solution of oxazine **1** (10 mg, 0.08 mmol), DMAP (0.1 mg, 0.1 µmol) and triethylamine (0.01 cm³, 10 mg, 0.1 mmol) in DCM (2 cm³) at 0 ºC. The reaction mixture was stirred for 2 hours before being poured into an aqueous 1*N* HCl solution (10 cm³). The organic phase was extracted with EtOAc (3 × 5 cm³) and the organic layers combined, washed with brine (10 cm³) and dried over magnesium sulfate. Removal of the solvent *in vacuo* yielded a yellow oil. Purification of the crude material by flash column chromatography on silica gel using an eluent of 10% ethyl acetate: *n*-hexanes afforded the title compound as a colourless oil (11 mg, 25%). δ_H (500 MHz, $CDCl_3$) 2.10 (3H, s, C*H₃*), 3.40 (3H, s, OC*H₃*), 3.88 (3H, s, OC*H3*), 6.97 (1H, d, *J* 3.0, ArC*H*), 7.24 (1H, d, *J* 3.0, ArC*H*), 7.40 – 7.42 (2H, m, ArC*H*), 7.41 – 7.65 (4H, m, ArC*H*), 7.85 (2H, dd, *J* 8.3 1.3, ArC*H*), 8.00 (2H, dd, *J* 8.4 1.1, ArC*H*); δ_C (125 MHz, $CDCl_3$) 20.7, 52.3, 56.2, 100.0, 108.1, 111.9, 119.8, 124.2, 125.9, 128.5 (2 × Ar*C*H), 128.8 (2 × Ar*C*H), 130.0 (2 × Ar*C*H), 130.2 (2 × Ar*C*H), 133.6, 134.1, 135.0, 145.5, 157.5, 161.9, 164.3, 165.7, 173.6; *m/z* (ESI-TOF) 4765.1348 (100%, MH^+, $C_{26}H_{22}NO_8$ requires 476.1345).

Methyl 2-(2-chloroacetoxy)-7-methoxy-2-methyl-3-oxo-3,4-dihydro-2*H*-benzo[*b*][1,4]oxazine-5-carboxylate **33**

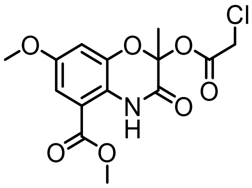

Triethylamine (12 µl, 0.08 mmol) was added dropwise to a solution of hemi-acetal **8** (10 mg, 0.04 mmol), chloroacetyl chloride (63 µl, 0.08 mmol) and DMAP (1 mg, 8 µmol) in DCM (5 cm³) at -10 °C. The mixture was stirred for 1 hour before the volatiles were removed *in vacuo* to yield a yellow oil. Purification of the crude material by flash column chromatography on silica gel using an eluent of 10% ethyl acetate: *n*-hexanes afforded the title compound as a white solid (3 mg, 25%). δ_H (600 MHz, $CDCl_3$) 1.95 (3H, s C*H₃*), 3.80 (3H, s, OC*H₃*), 3.95 (3H, s, OC*H₃*), 4.08 (2H, s, C*H₂*Cl), 6.75 (1H, d, *J* 2.8, ArC*H*), 7.21 (1H, d, *J* 2.8, ArC*H*), 10.48 (1H, s, N*H*); δ_C (150 MHz, $CDCl_3$) 23.6, 40.6, 62.6, 65.8, 99.1, 108.3, 114.0, 121.9, 142.2, 154.9, 160.7, 165.5, 166.9, 172.7; *m/z* (ESI-TOF) 344.0538 (100%, MH^+, $C_{14}H_{15}ClNO_7$ requires 344.0537).

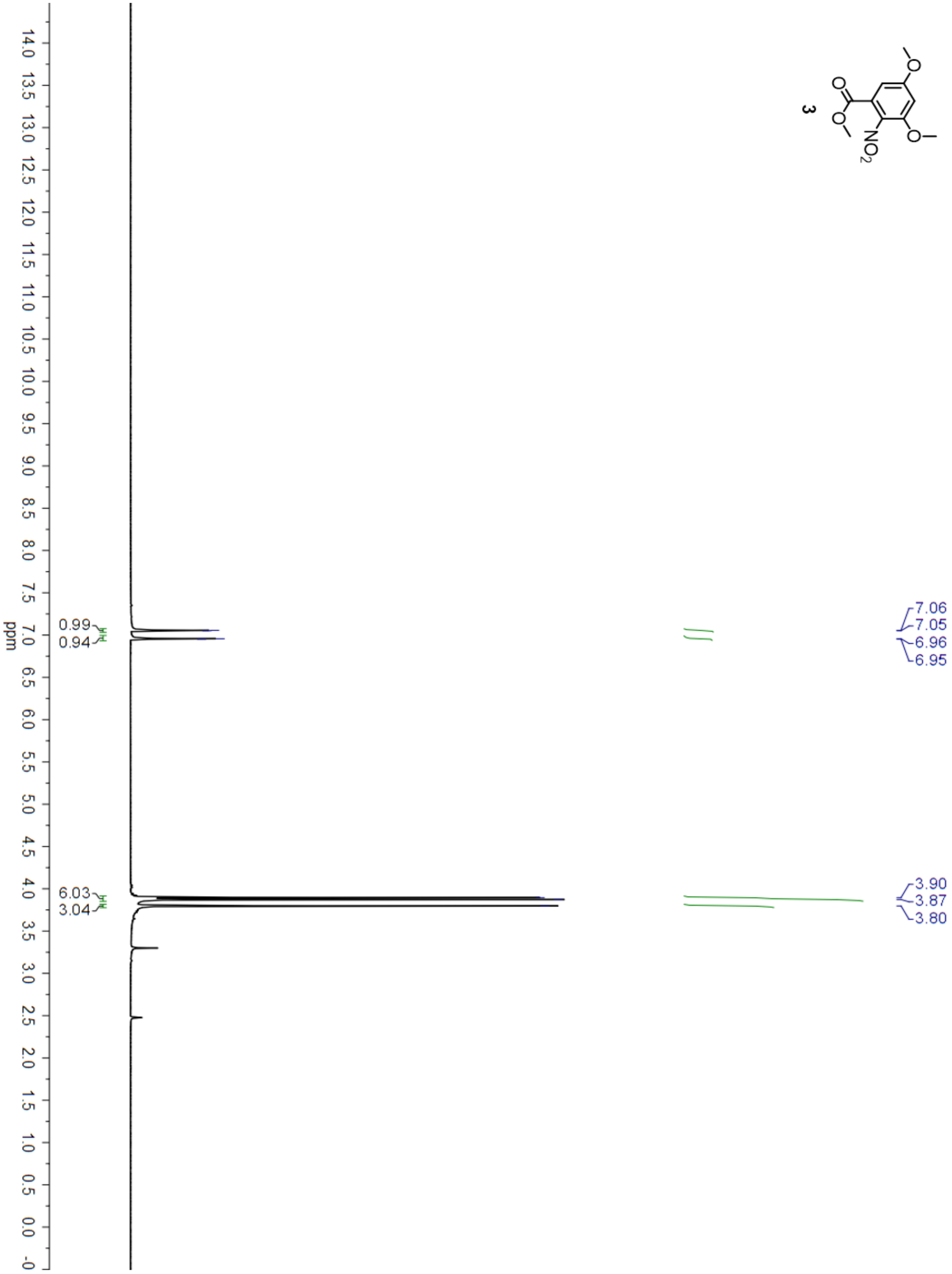
3
NO_2
7.06
7.05
6.96
6.95
3.90
3.87
3.80
0.99
0.94
6.03
3.04
ppm
14.0 13.5 13.0 12.5 12.0 11.5 11.0 10.5 10.0 9.5 9.0 8.5 8.0 7.5 7.0 6.5 6.0 5.5 5.0 4.5 4.0 3.5 3.0 2.5 2.0 1.5 1.0 0.5 0.0 -0

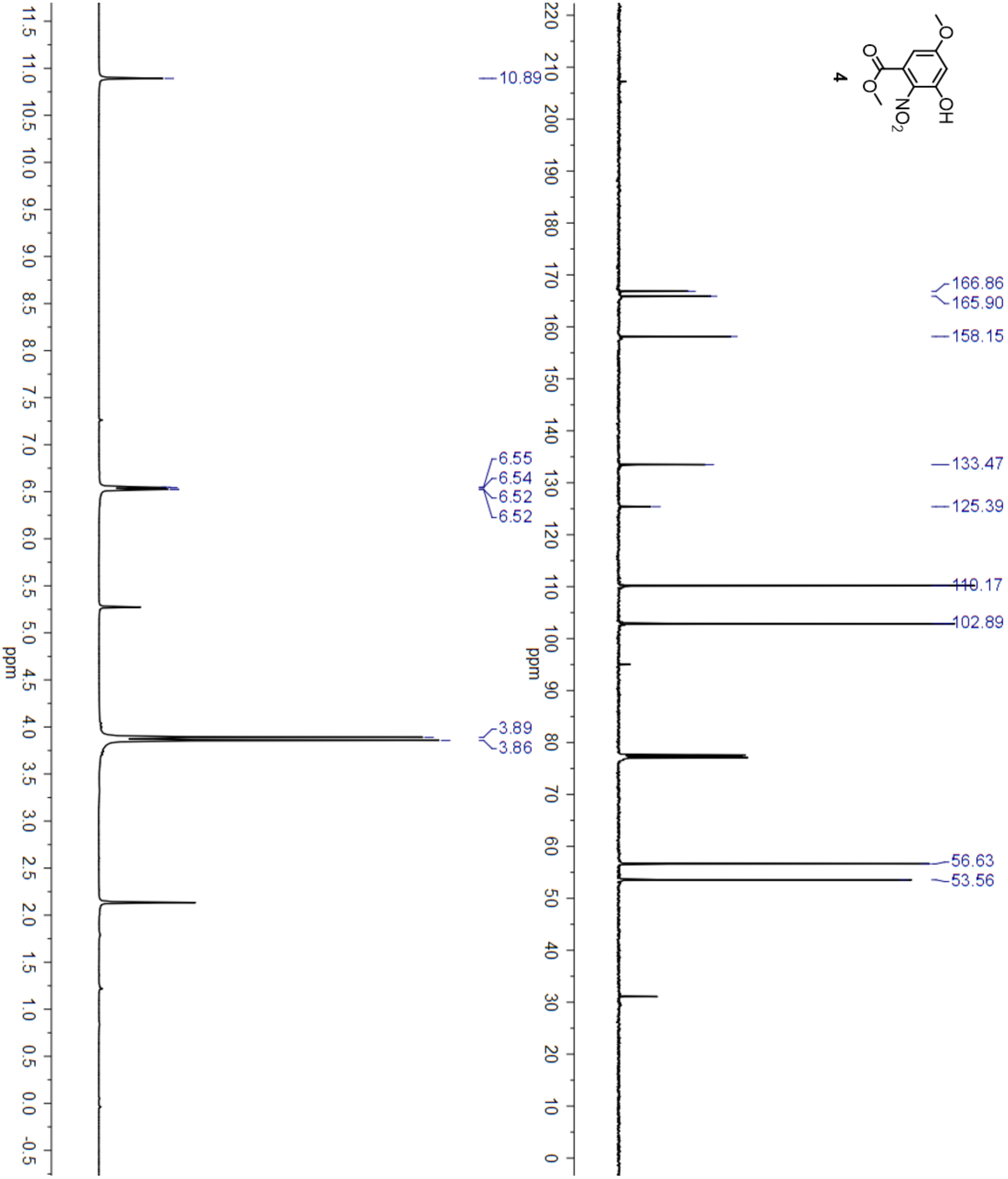
4
OH
NO_2
166.86
165.90
158.15
133.47
125.39
110.17
102.89
56.63
53.56
10.89
6.55
6.54
6.52
6.52
3.89
3.86
ppm
ppm

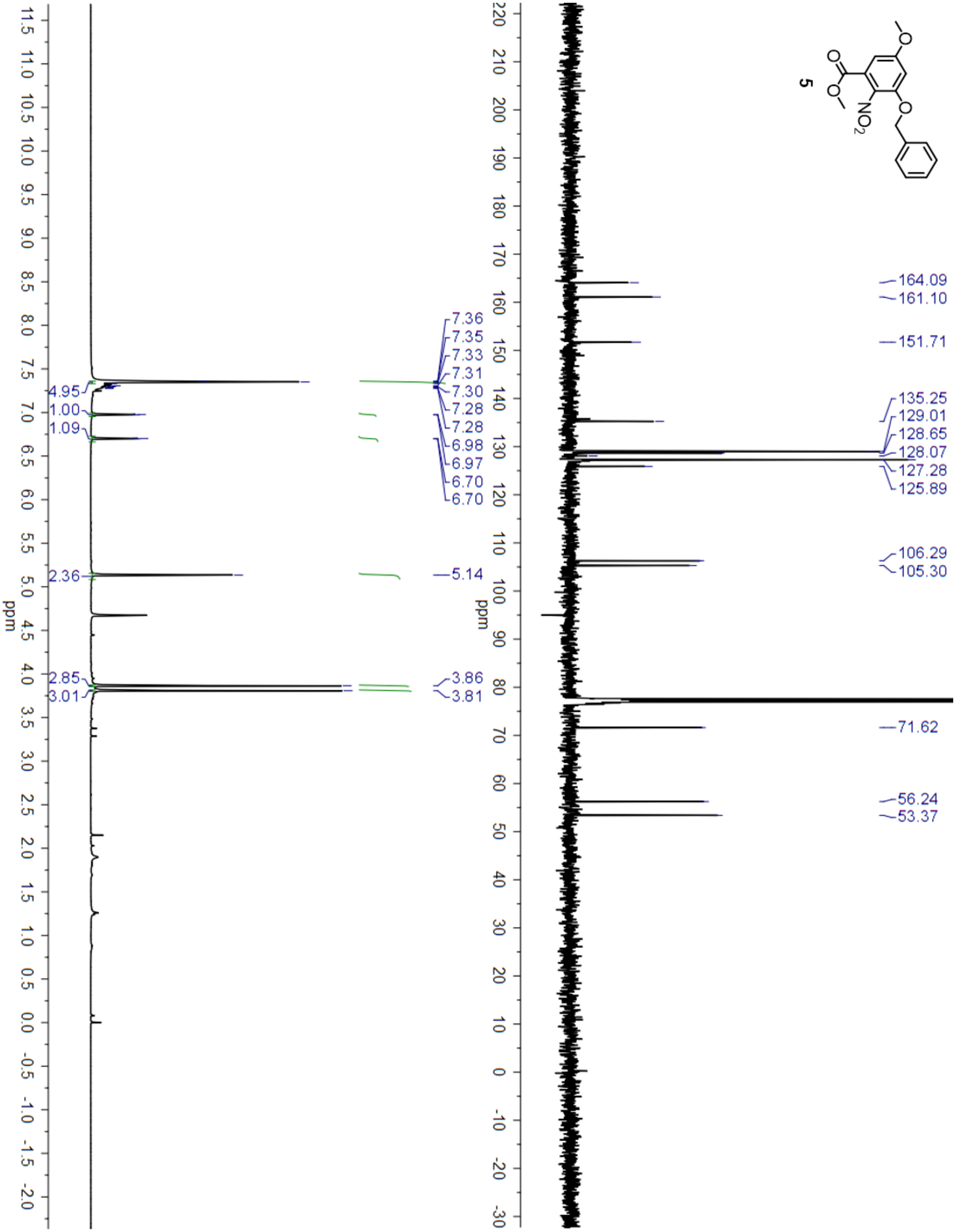

5
NO_2
164.09
161.10
151.71
135.25
129.01
128.65
128.07
127.28
125.89
106.29
105.30
71.62
56.24
53.37
7.36
7.35
7.33
7.31
7.30
7.28
7.28
6.98
6.97
6.70
6.70
5.14
3.86
3.81
4.95
1.00
1.09
2.36
2.85
3.01
ppm
ppm

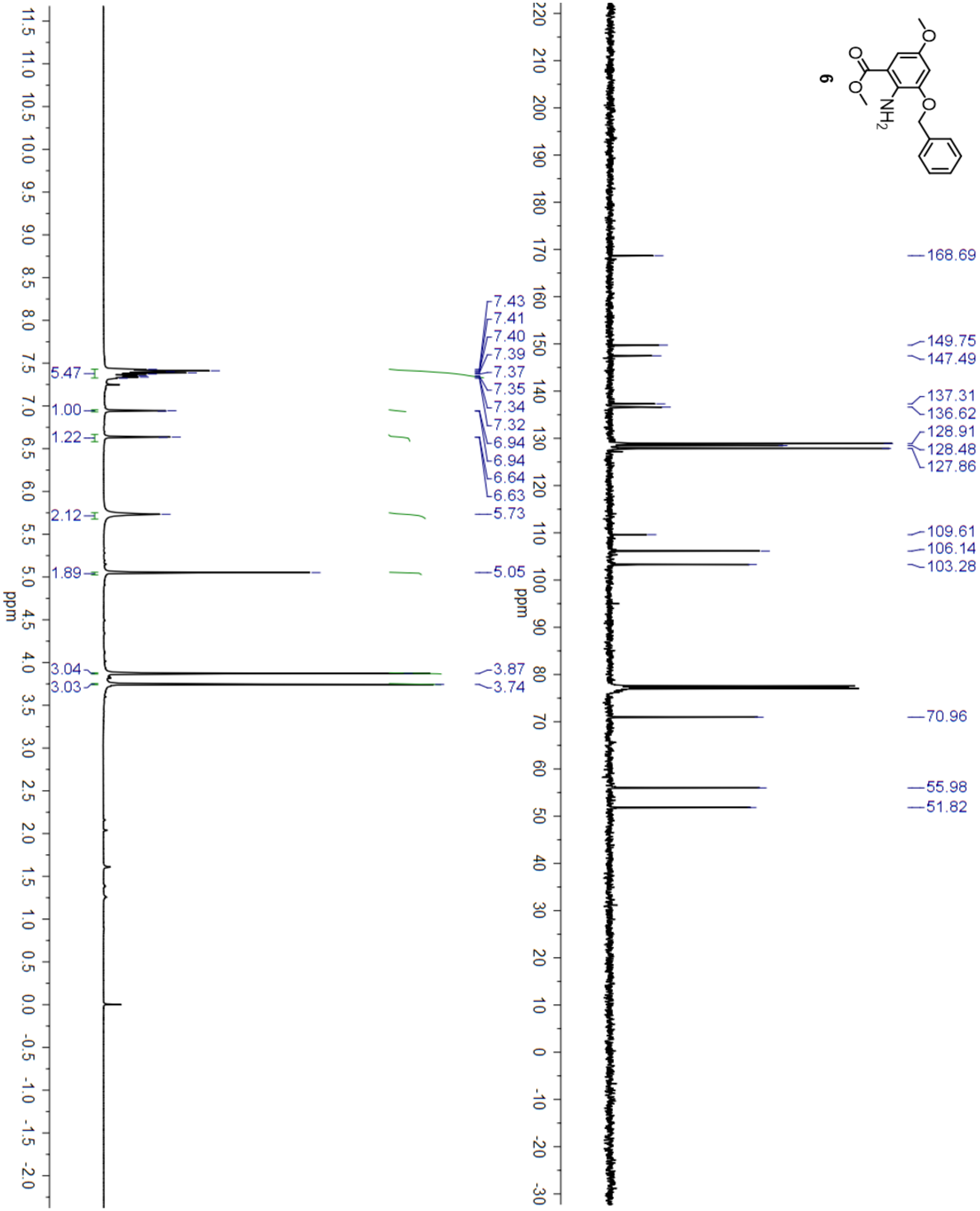
6
NH2
168.69
149.75
147.49
137.31
136.62
128.91
128.48
127.86
109.61
106.14
103.28
70.96
55.98
51.82
7.43
7.41
7.40
7.39
7.37
7.35
7.34
7.32
6.94
6.94
6.64
6.63
5.73
5.05
3.87
3.74
5.47
1.00
1.22
2.12
1.89
3.04
3.03
ppm
ppm

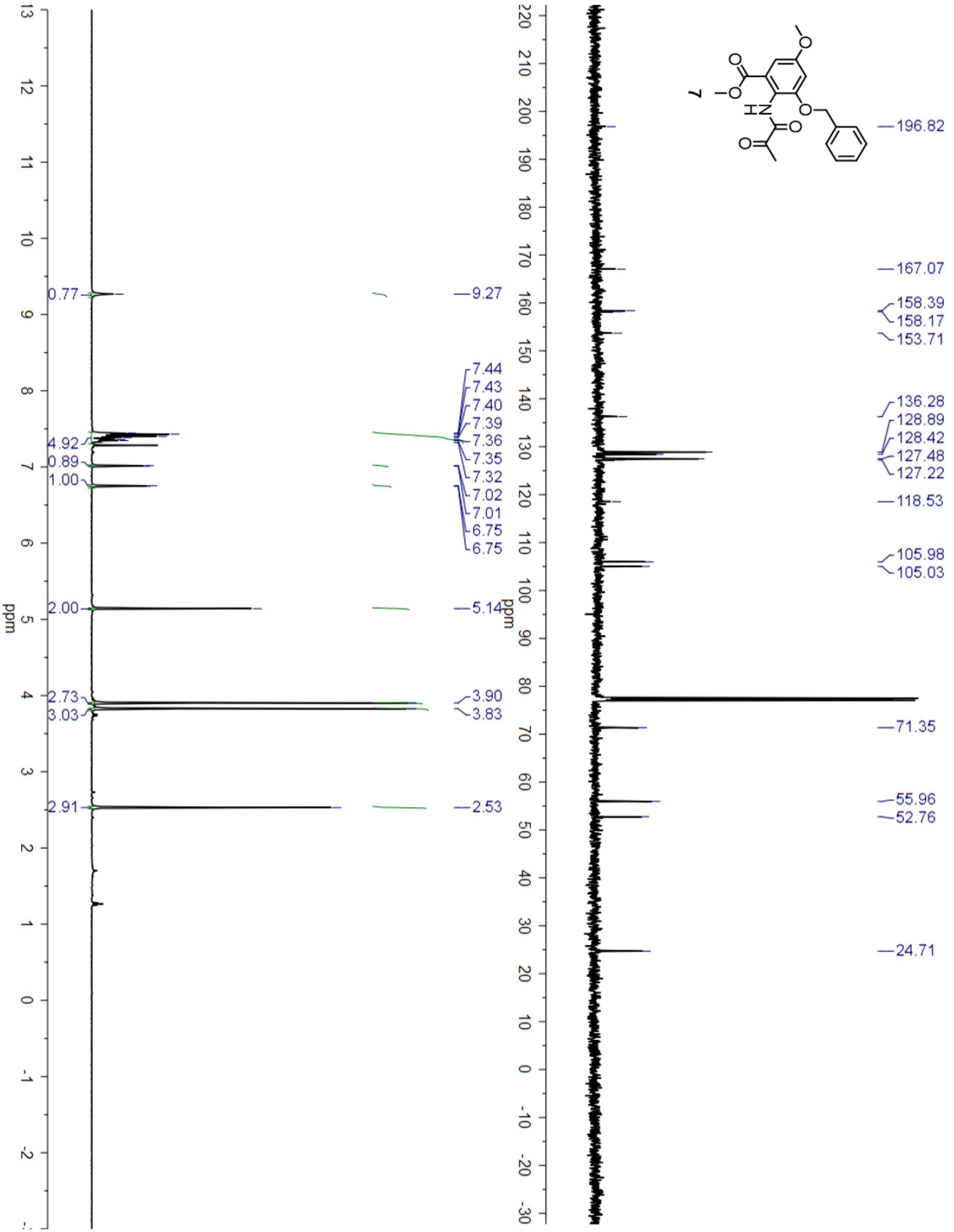
7
196.82
167.07
158.39
158.17
153.71
136.28
128.89
128.42
127.48
127.22
118.53
105.98
105.03
71.35
55.96
52.76
24.71
9.27
7.44
7.43
7.40
7.39
7.36
7.35
7.32
7.02
7.01
6.75
6.75
5.14
3.90
3.83
2.53
0.77
4.92
0.89
1.00
2.00
2.73
3.03
2.91
ppm
ppm

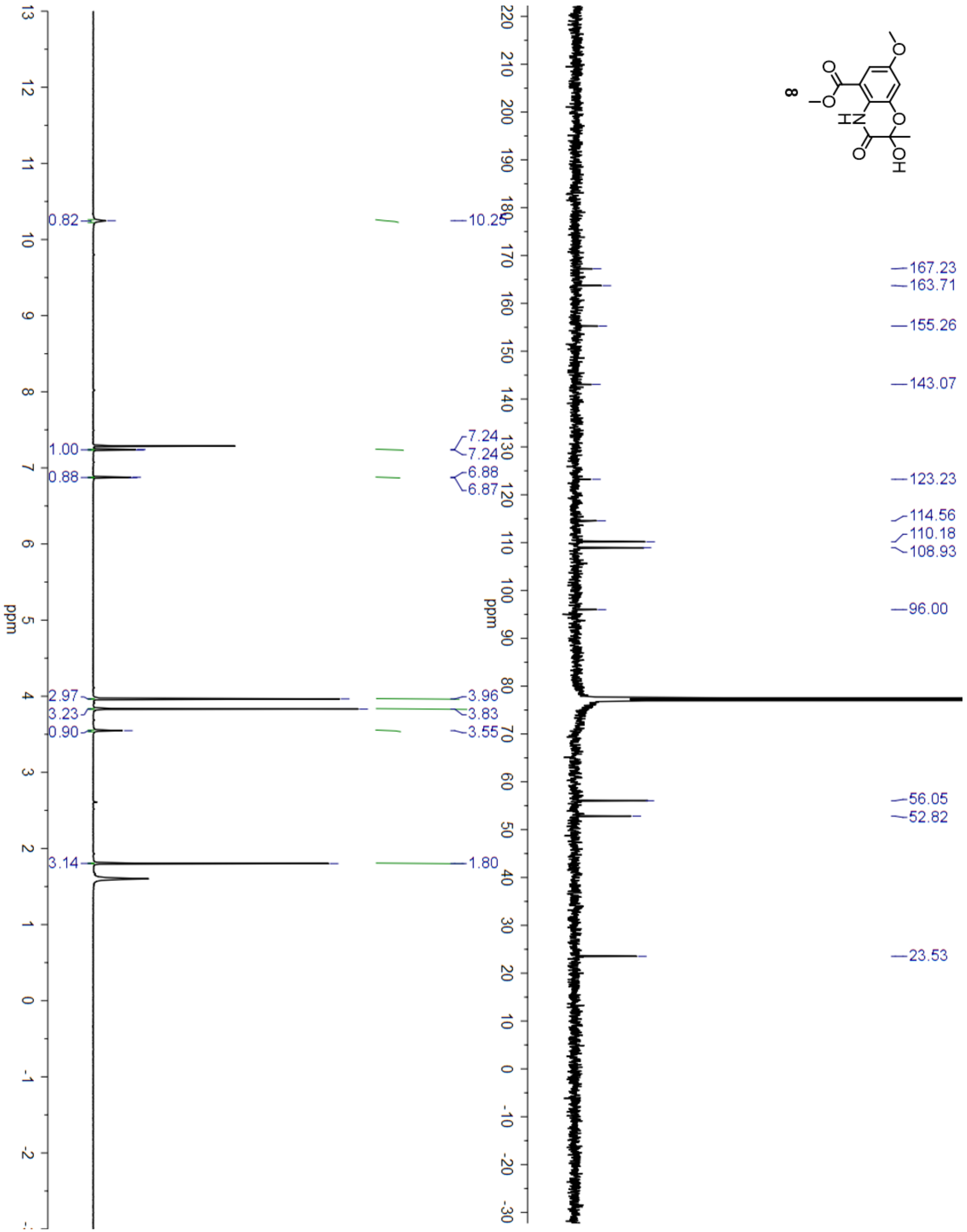
8
10.25
7.24
7.24
6.88
6.87
3.96
3.83
3.55
1.80
0.82
1.00
0.88
2.97
3.23
0.90
3.14
ppm
167.23
163.71
155.26
143.07
123.23
114.56
110.18
108.93
96.00
56.05
52.82
23.53
ppm

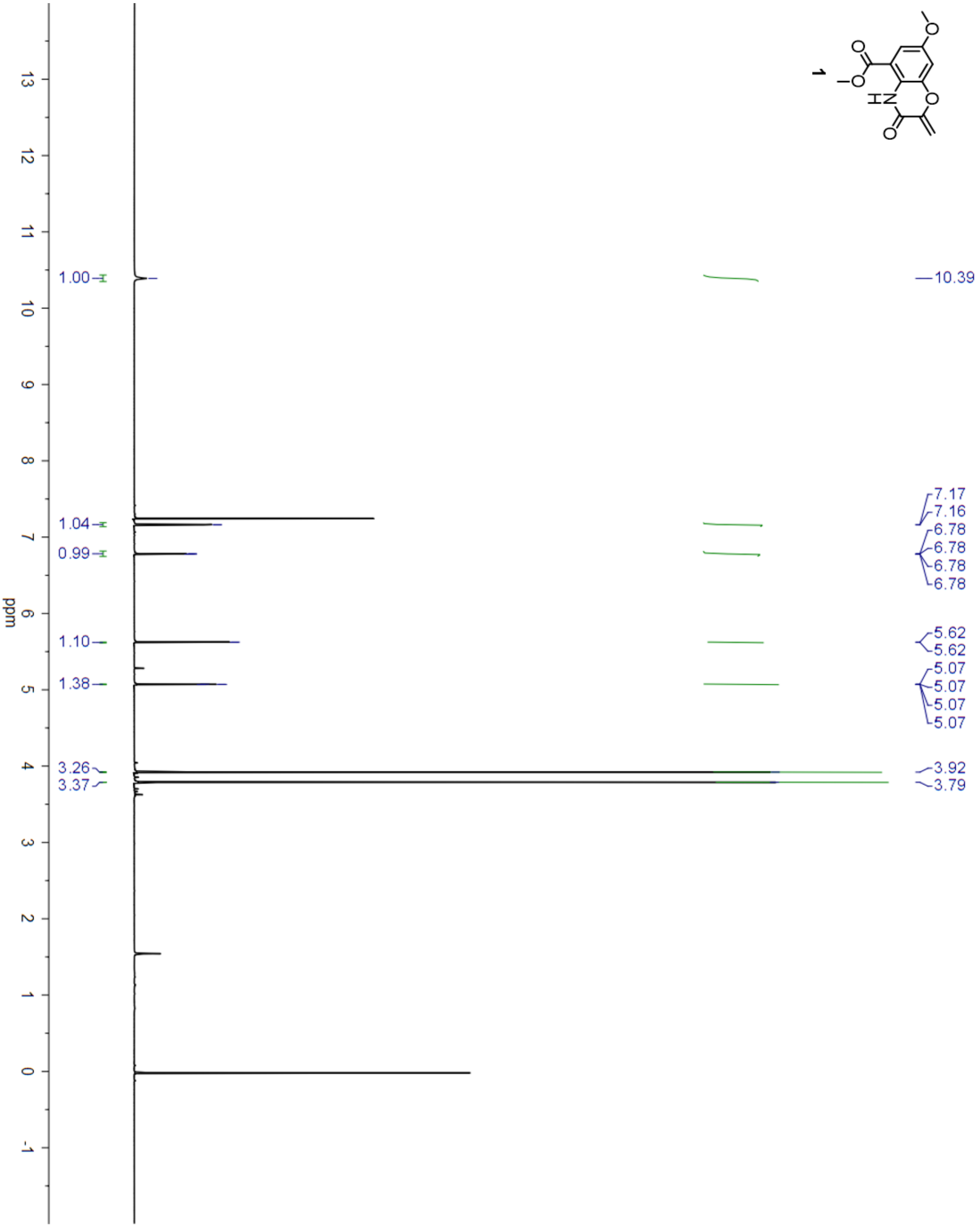
1
ppm
1.00
1.04
0.99
1.10
1.38
3.26
3.37
10.39
7.17
7.16
6.78
6.78
6.78
6.78
5.62
5.62
5.07
5.07
5.07
5.07
3.92
3.79

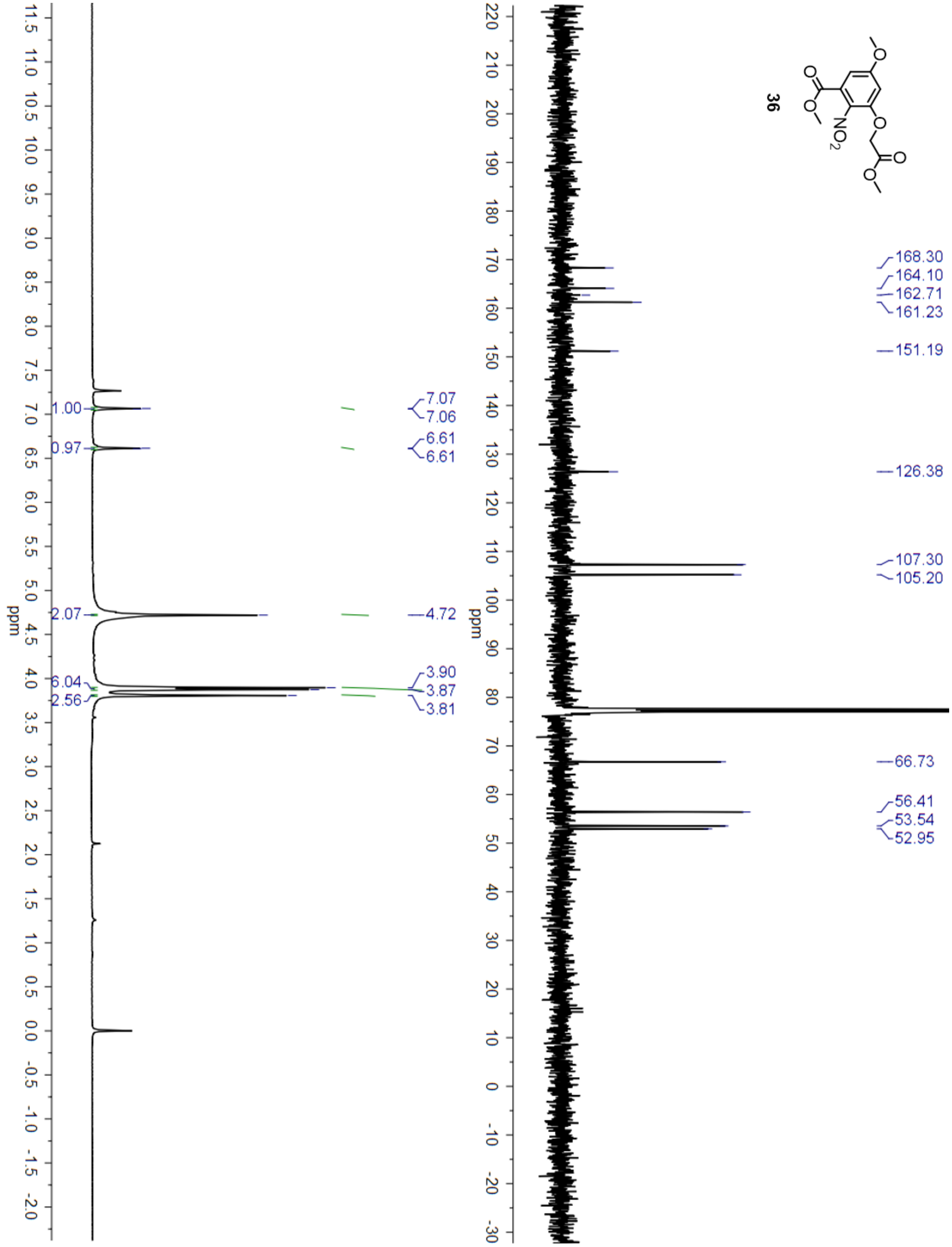
36
NO2
168.30
164.10
162.71
161.23
151.19
126.38
107.30
105.20
66.73
56.41
53.54
52.95
ppm
7.07
7.06
6.61
6.61
4.72
3.90
3.87
3.81
1.00
0.97
2.07
6.04
2.56
ppm

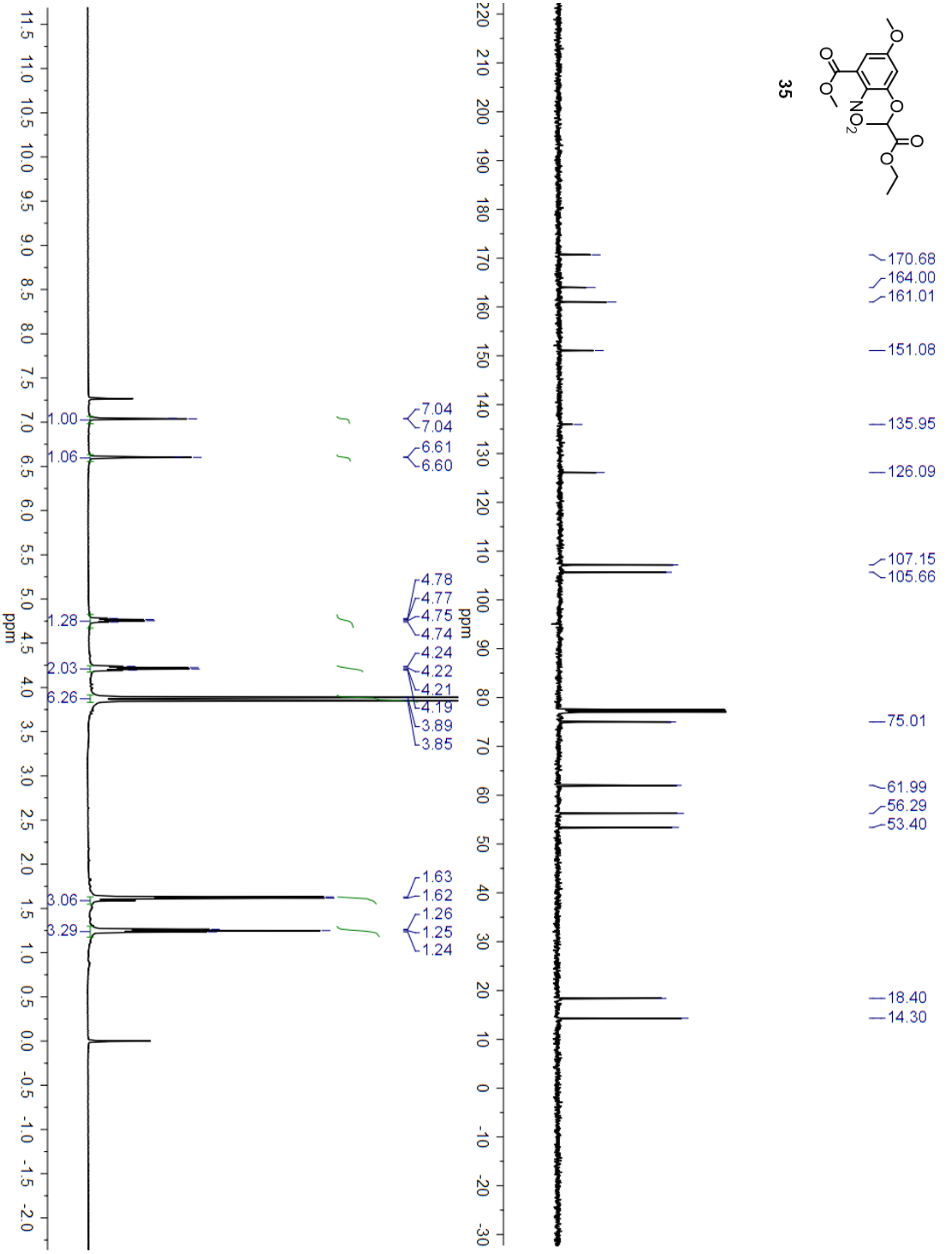
35
NO2
170.68
164.00
161.01
151.08
135.95
126.09
107.15
105.66
75.01
61.99
56.29
53.40
18.40
14.30
7.04
7.04
6.61
6.60
4.78
4.77
4.75
4.74
4.24
4.22
4.21
4.19
3.89
3.85
1.63
1.62
1.26
1.25
1.24
1.00
1.06
1.28
2.03
6.26
3.06
3.29
ppm
ppm

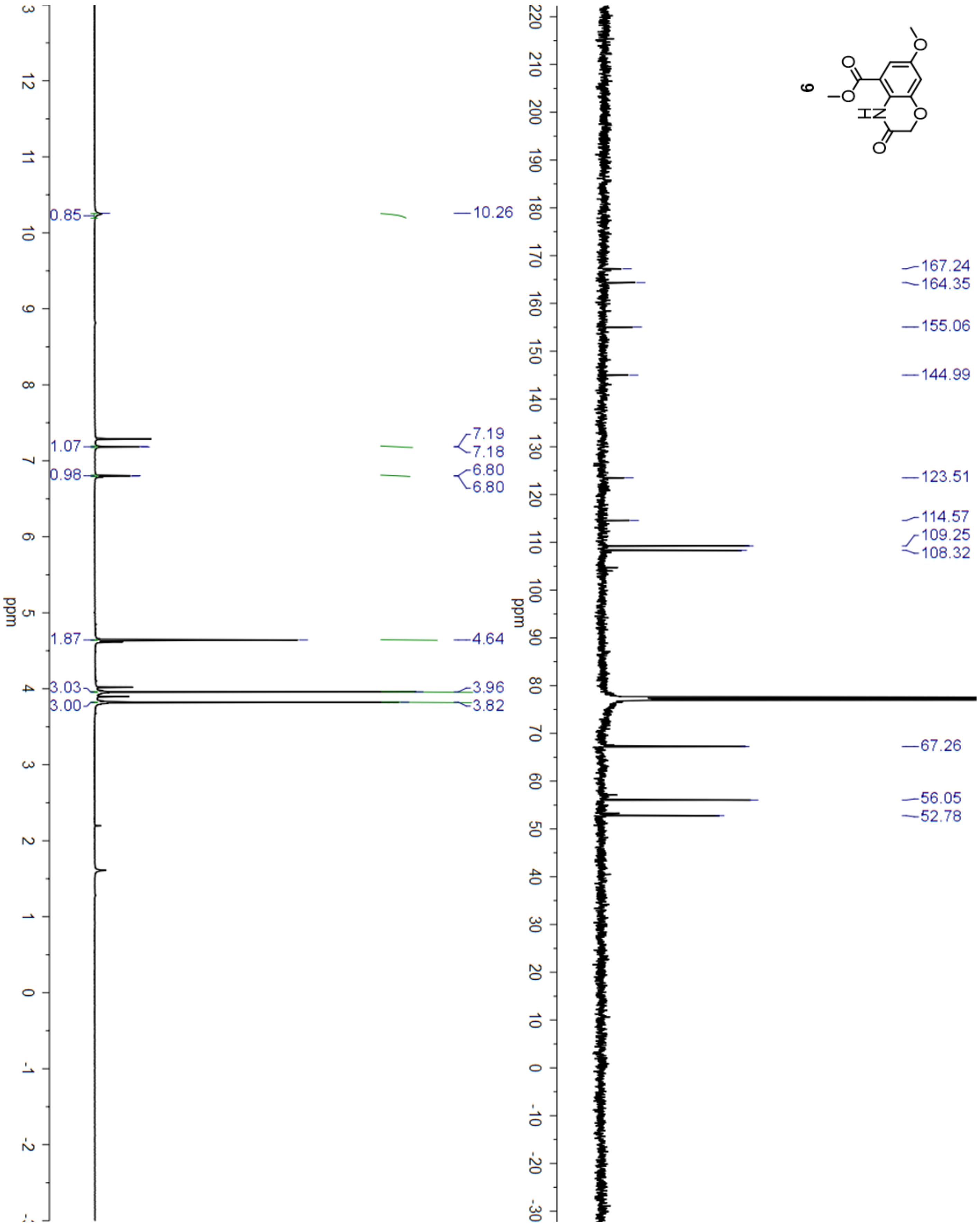
9
10.26
7.19
7.18
6.80
6.80
4.64
3.96
3.82
0.85
1.07
0.98
1.87
3.03
3.00
ppm
167.24
164.35
155.06
144.99
123.51
114.57
109.25
108.32
67.26
56.05
52.78
ppm

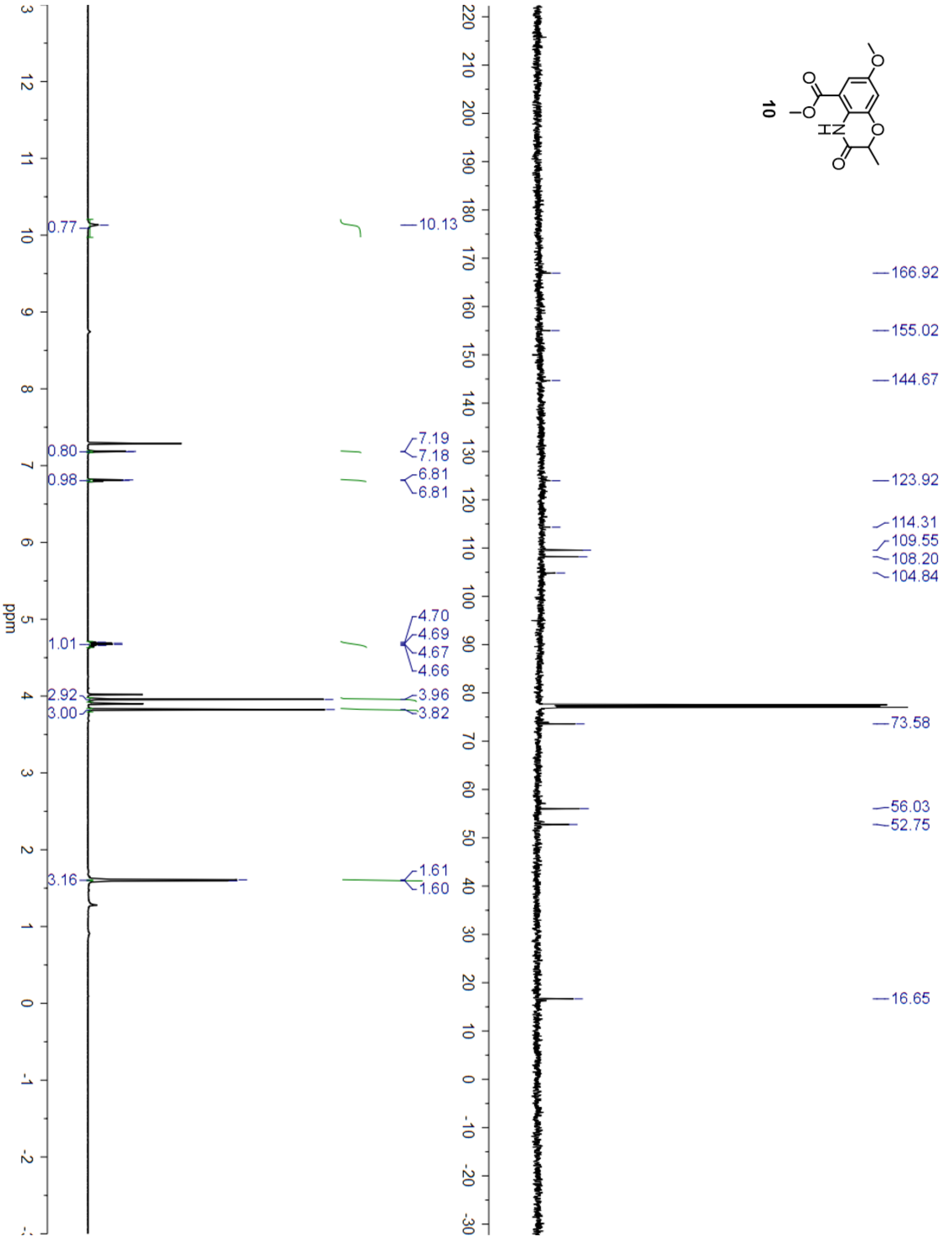
10
10.13
7.19
7.18
6.81
6.81
4.70
4.69
4.67
4.66
3.96
3.82
1.61
1.60
0.77
0.80
0.98
1.01
2.92
3.00
3.16
ppm
166.92
155.02
144.67
123.92
114.31
109.55
108.20
104.84
73.58
56.03
52.75
16.65

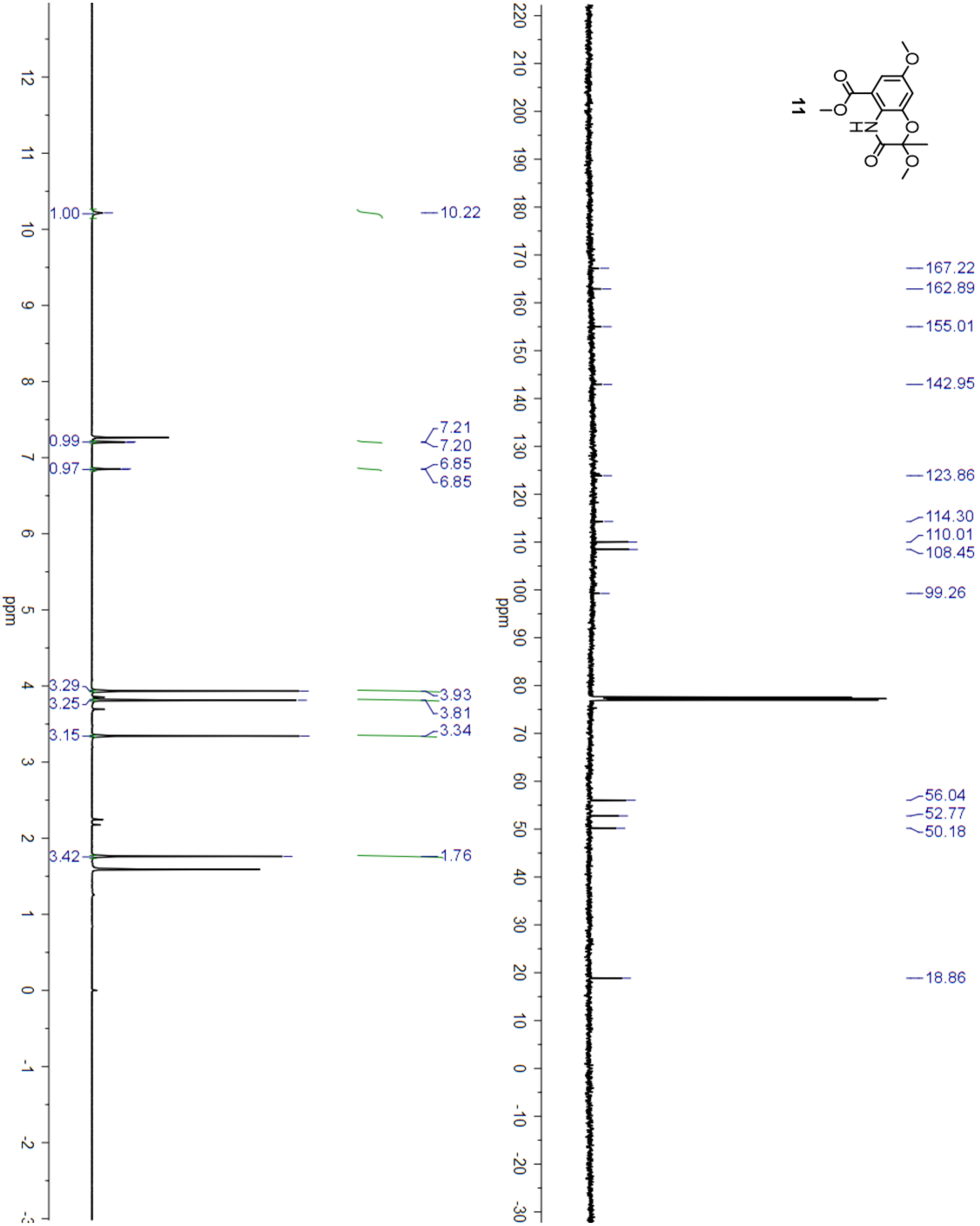
11
1.00
10.22
0.99
7.21
7.20
0.97
6.85
6.85
3.29
3.93
3.25
3.81
3.15
3.34
3.42
1.76
ppm
167.22
162.89
155.01
142.95
123.86
114.30
110.01
108.45
99.26
56.04
52.77
50.18
18.86
ppm

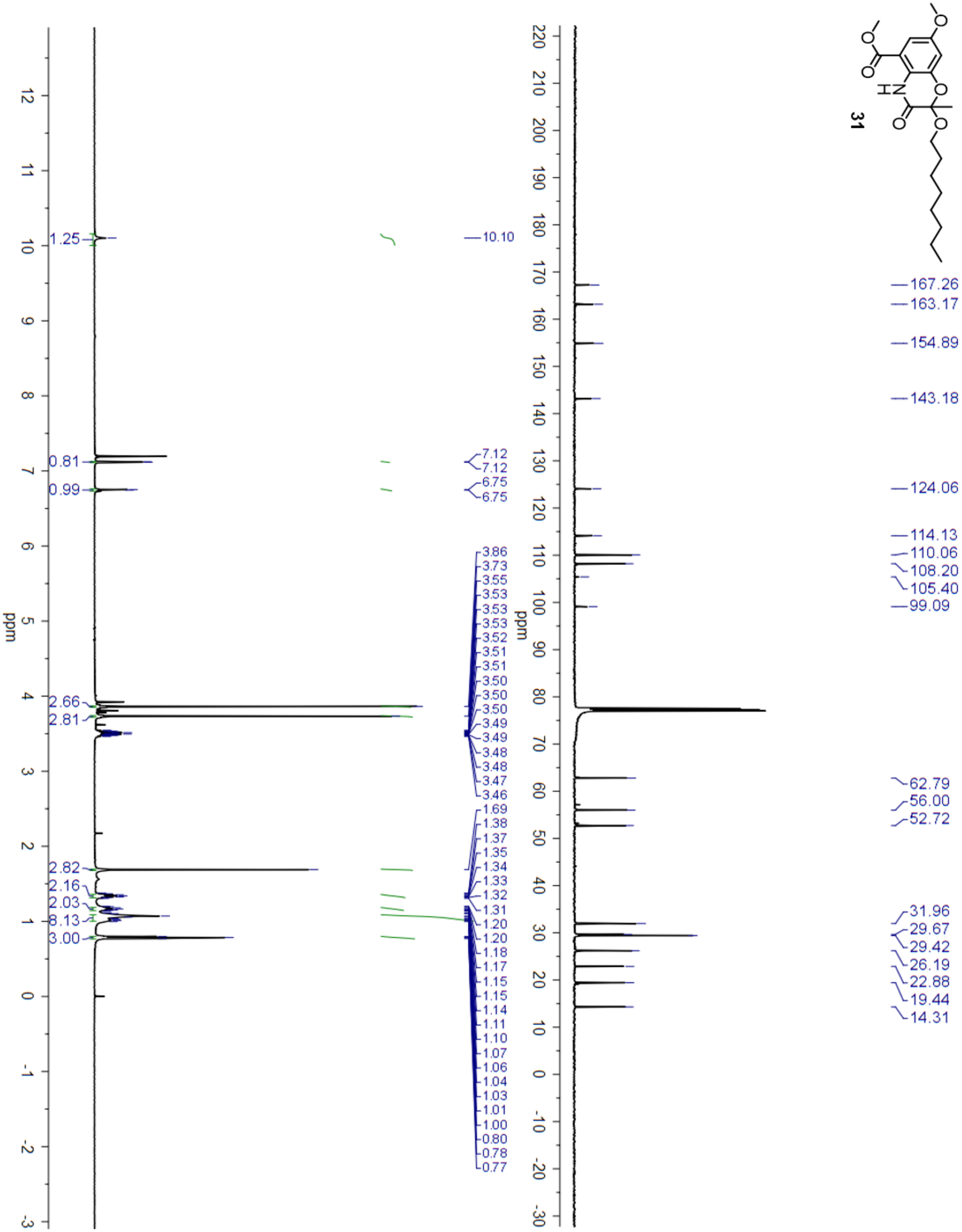
31

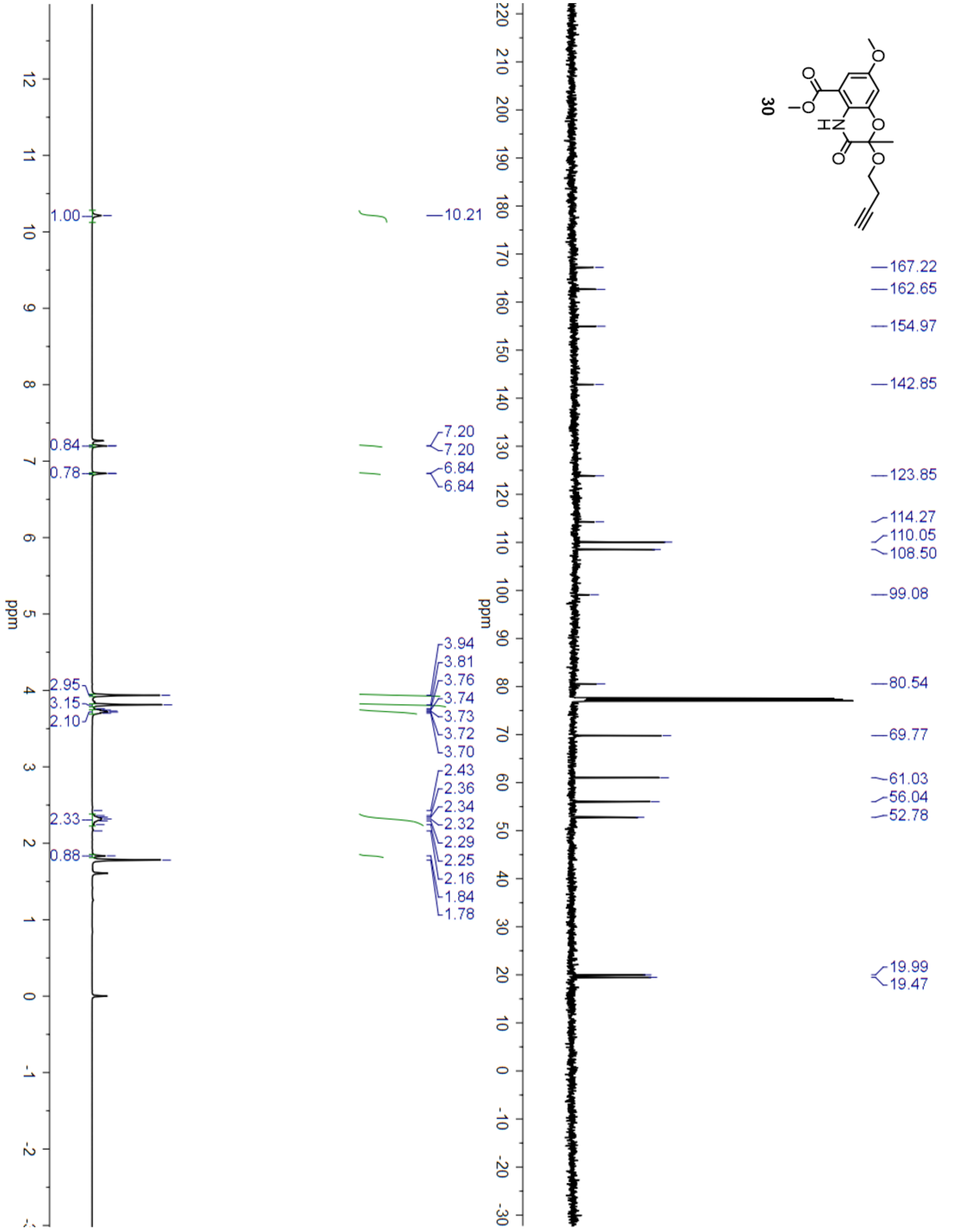
30
10.21
7.20
7.20
6.84
6.84
3.94
3.81
3.76
3.74
3.73
3.72
3.70
2.43
2.36
2.34
2.32
2.29
2.25
2.16
1.84
1.78
1.00
0.84
0.78
2.95
3.15
2.10
2.33
0.88
ppm
167.22
162.65
154.97
142.85
123.85
114.27
110.05
108.50
99.08
80.54
69.77
61.03
56.04
52.78
19.99
19.47
ppm

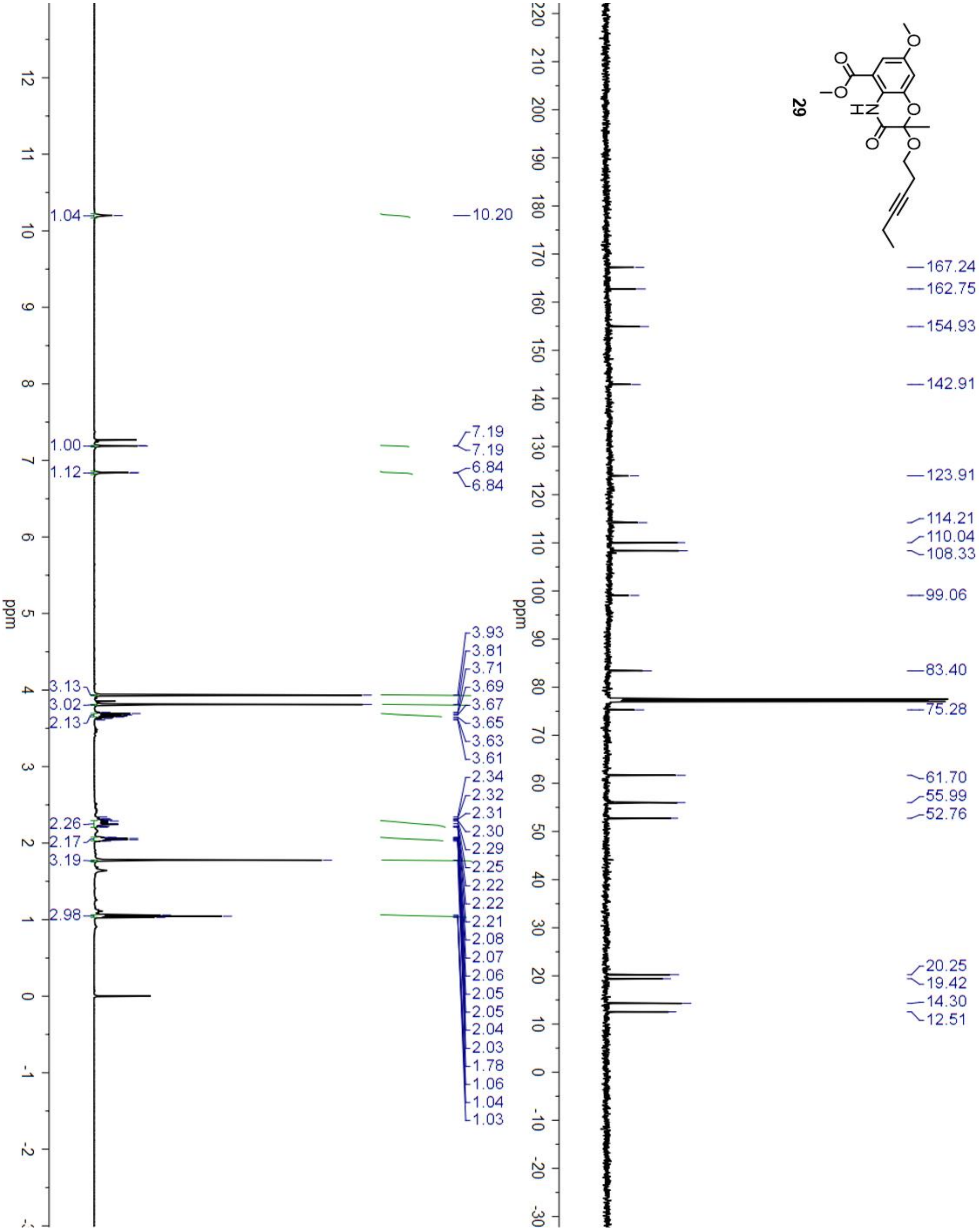
29
10.20
7.19
7.19
6.84
6.84
3.93
3.81
3.71
3.69
3.67
3.65
3.63
3.61
2.34
2.32
2.31
2.30
2.29
2.25
2.22
2.22
2.21
2.08
2.07
2.06
2.05
2.05
2.04
2.03
1.78
1.06
1.04
1.03
1.04
1.00
1.12
3.13
3.02
2.13
2.26
2.17
3.19
2.98
ppm
167.24
162.75
154.93
142.91
123.91
114.21
110.04
108.33
99.06
83.40
75.28
61.70
55.99
52.76
20.25
19.42
14.30
12.51
ppm

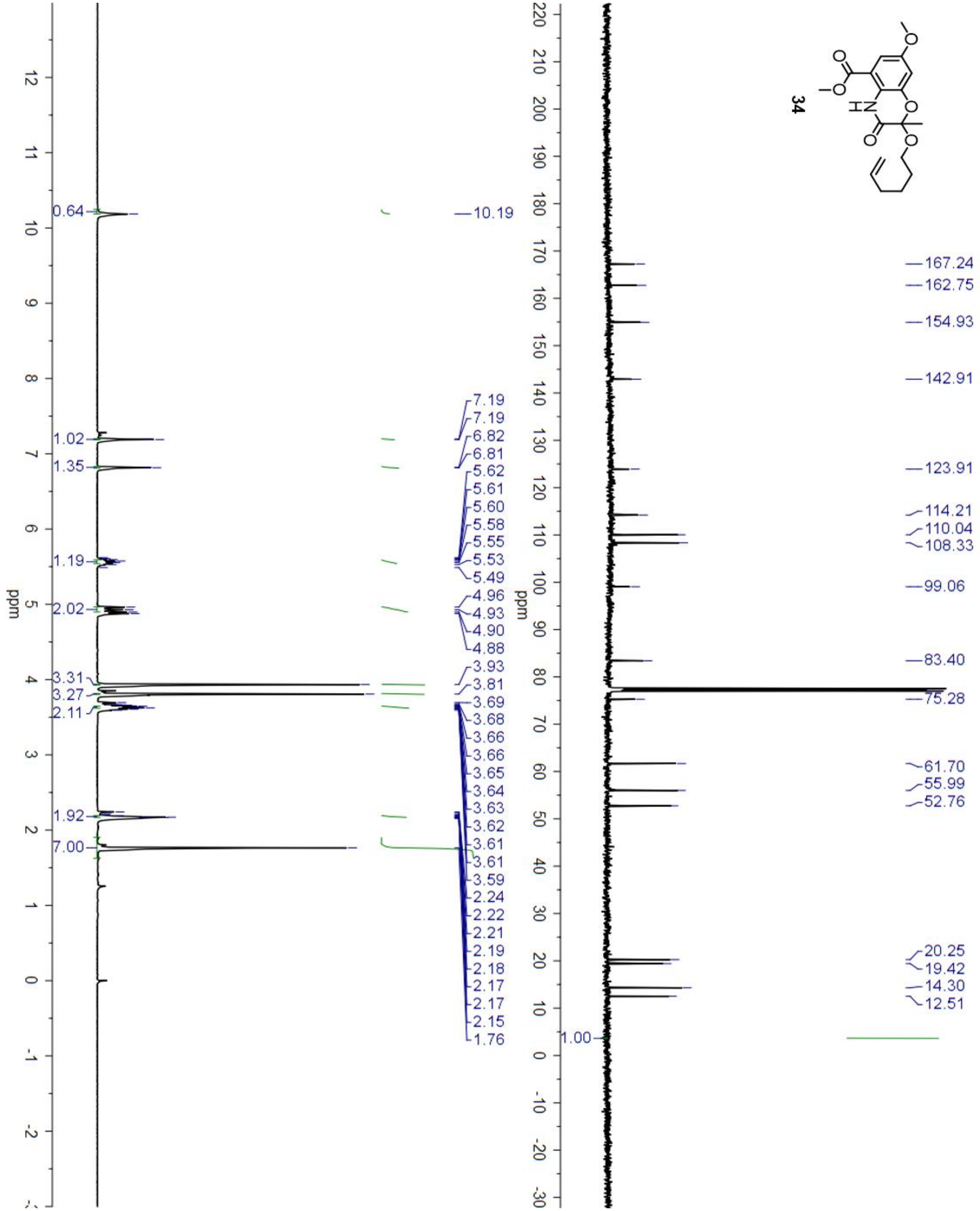

34
167.24
162.75
154.93
142.91
123.91
114.21
110.04
108.33
99.06
83.40
75.28
61.70
55.99
52.76
20.25
19.42
14.30
12.51
1.00
ppm
10.19
7.19
7.19
6.82
6.81
5.62
5.61
5.60
5.58
5.55
5.53
5.49
4.96
4.93
4.90
4.88
3.93
3.81
3.69
3.68
3.66
3.66
3.65
3.64
3.63
3.62
3.61
3.61
3.59
2.24
2.22
2.21
2.19
2.18
2.17
2.17
2.15
1.76
0.64
1.02
1.35
1.19
2.02
3.31
3.27
2.11
1.92
7.00
ppm

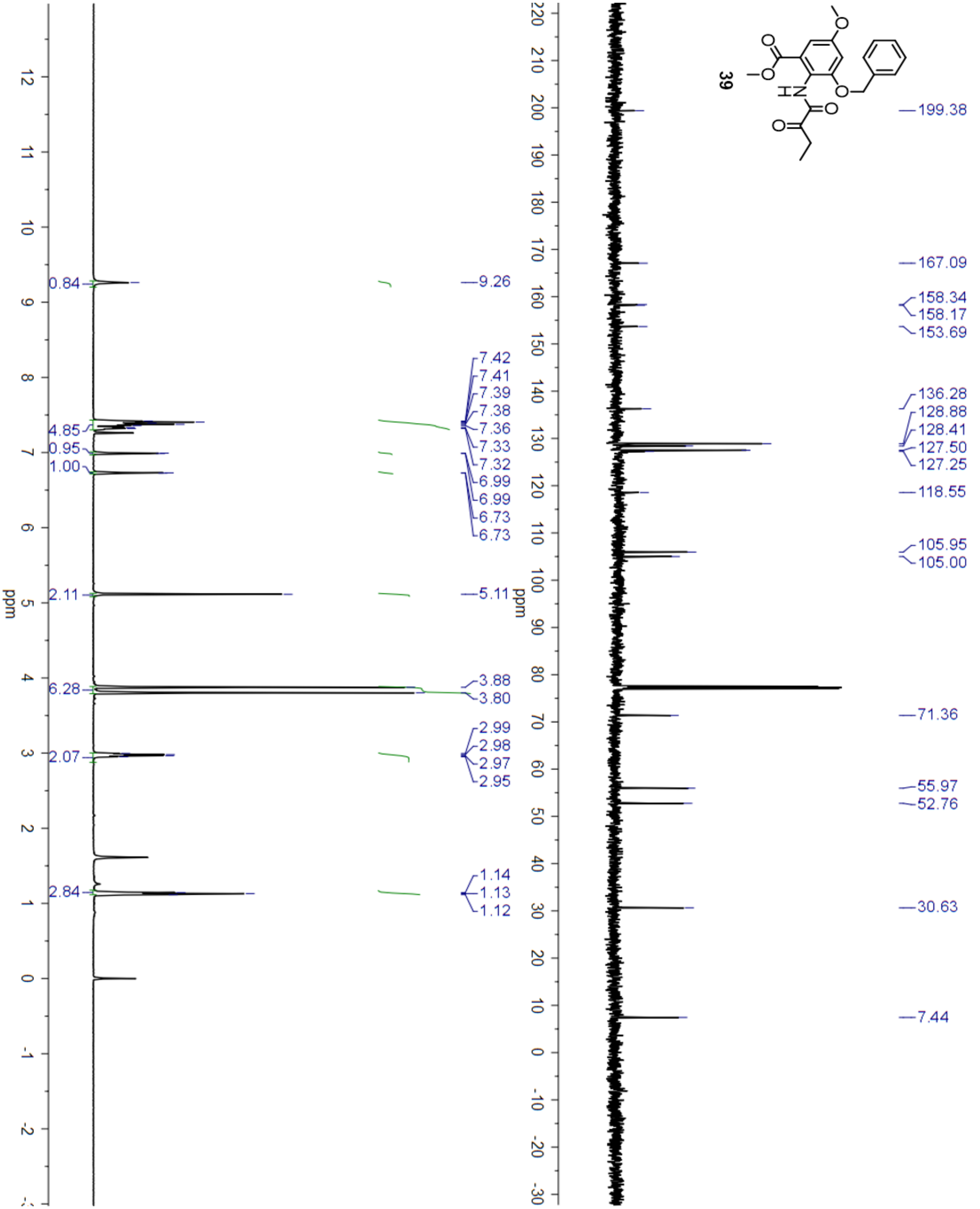
39
199.38
167.09
158.34
158.17
153.69
136.28
128.88
128.41
127.50
127.25
118.55
105.95
105.00
71.36
55.97
52.76
30.63
7.44
9.26
7.42
7.41
7.39
7.38
7.36
7.33
7.32
6.99
6.99
6.73
6.73
5.11
3.88
3.80
2.99
2.98
2.97
2.95
1.14
1.13
1.12
0.84
4.85
0.95
1.00
2.11
6.28
2.07
2.84
ppm
ppm

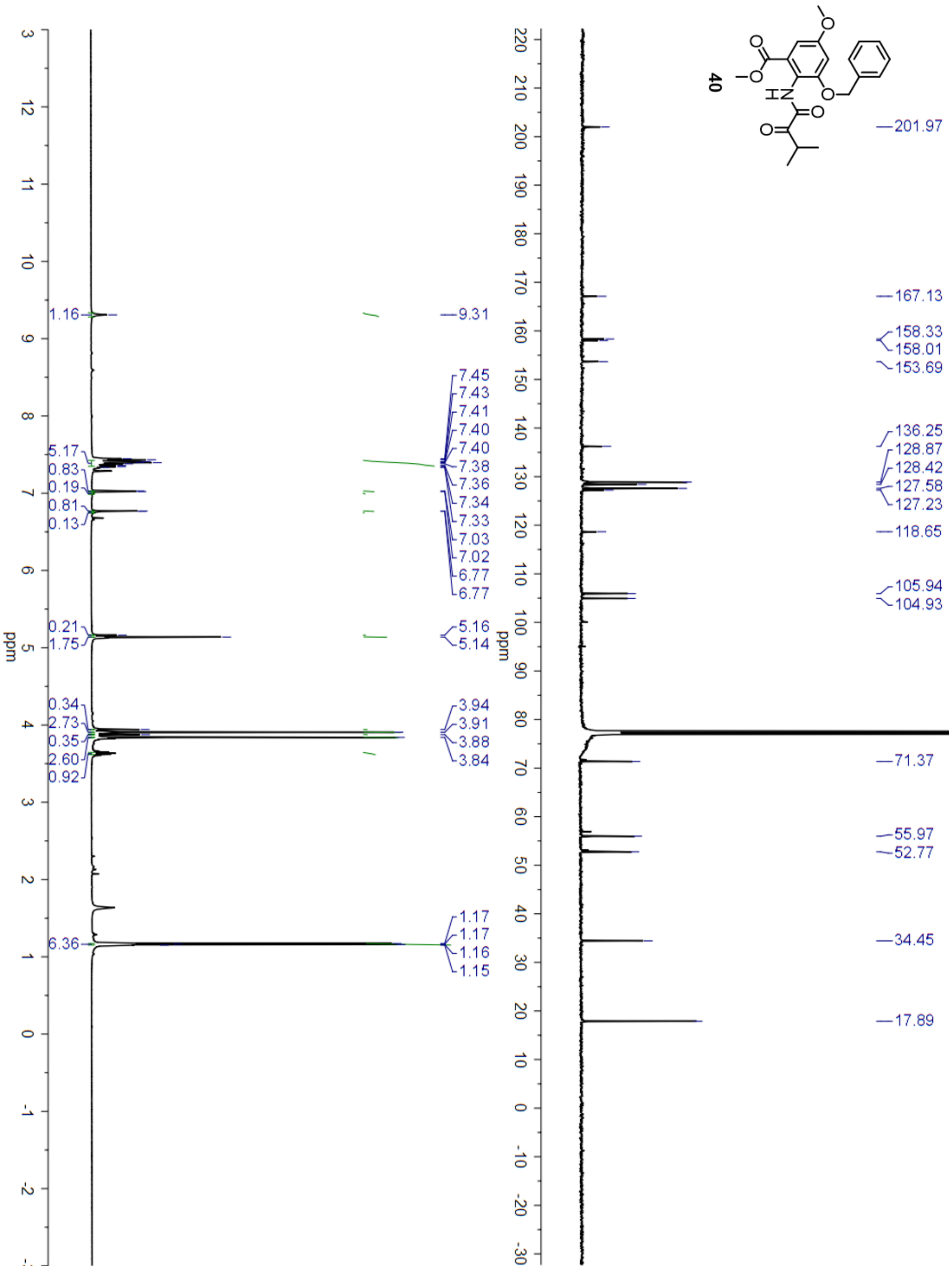
40
201.97
167.13
158.33
158.01
153.69
136.25
128.87
128.42
127.58
127.23
118.65
105.94
104.93
71.37
55.97
52.77
34.45
17.89
9.31
7.45
7.43
7.41
7.40
7.40
7.38
7.36
7.34
7.33
7.03
7.02
6.77
6.77
5.16
5.14
3.94
3.91
3.88
3.84
1.17
1.17
1.16
1.15
1.16
5.17
0.83
0.19
0.81
0.13
0.21
1.75
0.34
2.73
0.35
2.60
0.92
6.36
ppm
ppm

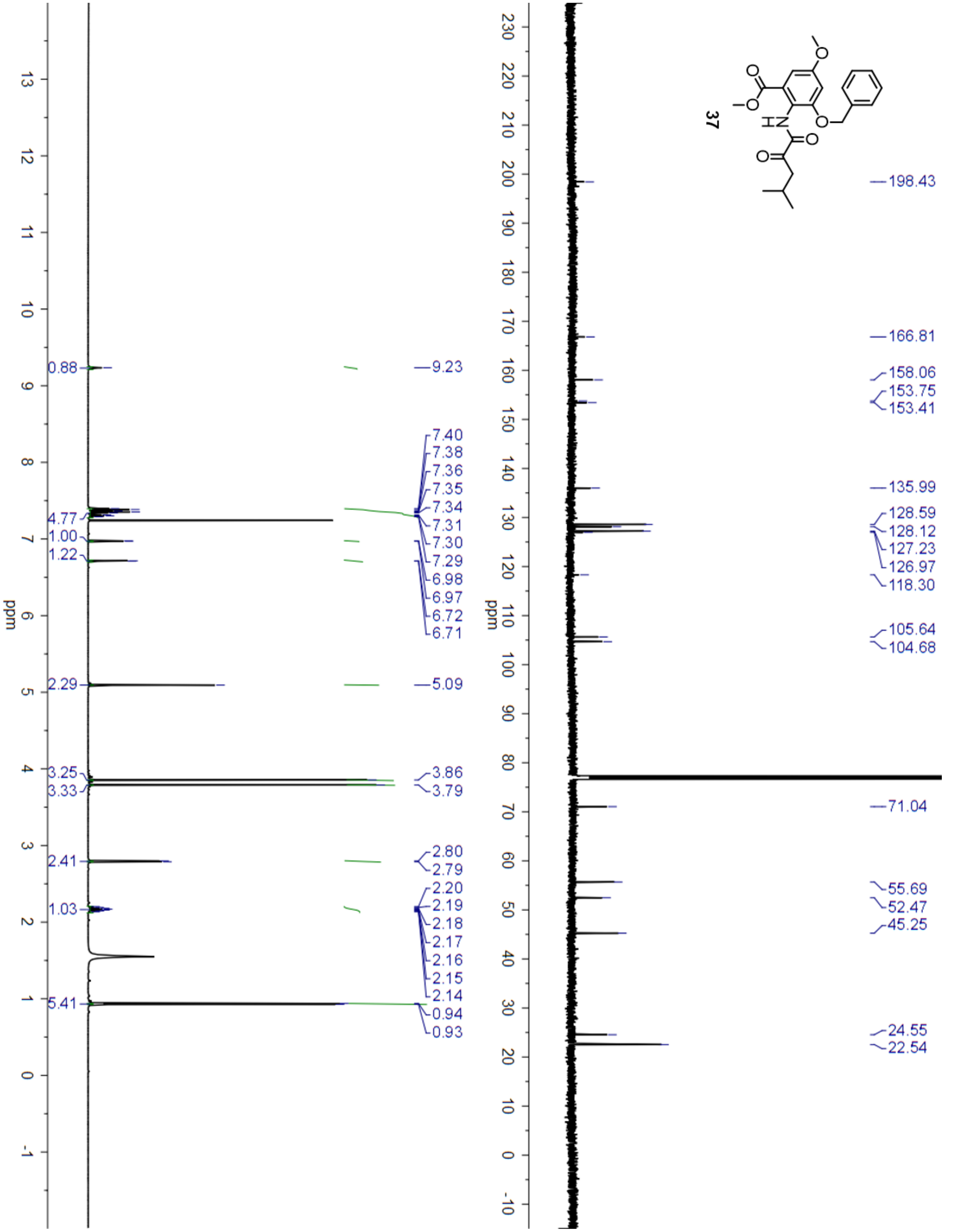
37
198.43
166.81
158.06
153.75
153.41
135.99
128.59
128.12
127.23
126.97
118.30
105.64
104.68
71.04
55.69
52.47
45.25
24.55
22.54
9.23
7.40
7.38
7.36
7.35
7.34
7.31
7.30
7.29
6.98
6.97
6.72
6.71
5.09
3.86
3.79
2.80
2.79
2.20
2.19
2.18
2.17
2.16
2.15
2.14
0.94
0.93
0.88
4.77
1.00
1.22
2.29
3.25
3.33
2.41
1.03
5.41
ppm
ppm

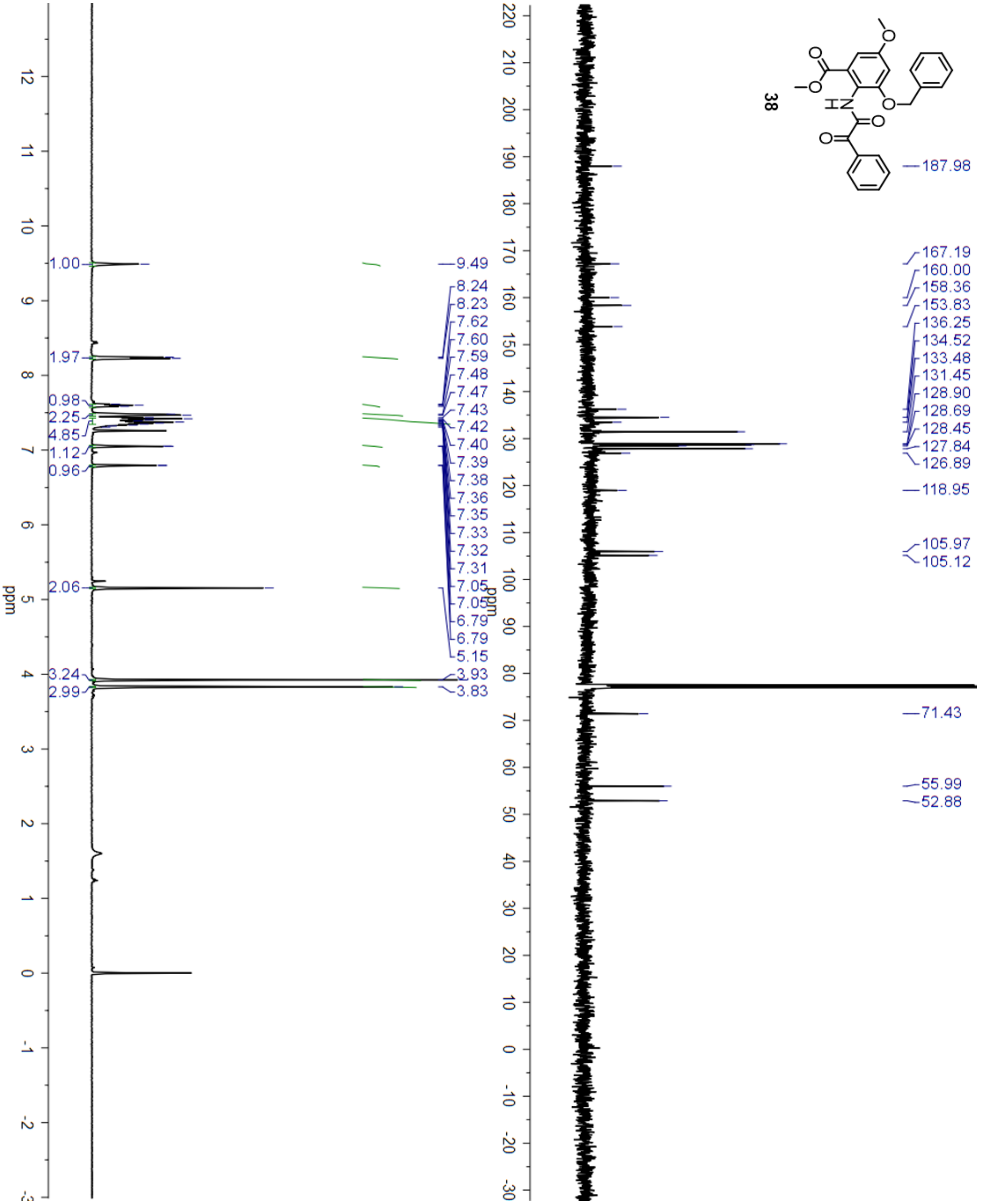
38
187.98
167.19
160.00
158.36
153.83
136.25
134.52
133.48
131.45
128.90
128.69
128.45
127.84
126.89
118.95
105.97
105.12
71.43
55.99
52.88
9.49
8.24
8.23
7.62
7.60
7.59
7.48
7.47
7.43
7.42
7.40
7.39
7.38
7.36
7.35
7.33
7.32
7.31
7.05
7.05
6.79
6.79
5.15
3.93
3.83
1.00
1.97
0.98
2.25
4.85
1.12
0.96
2.06
3.24
2.99
ppm
ppm

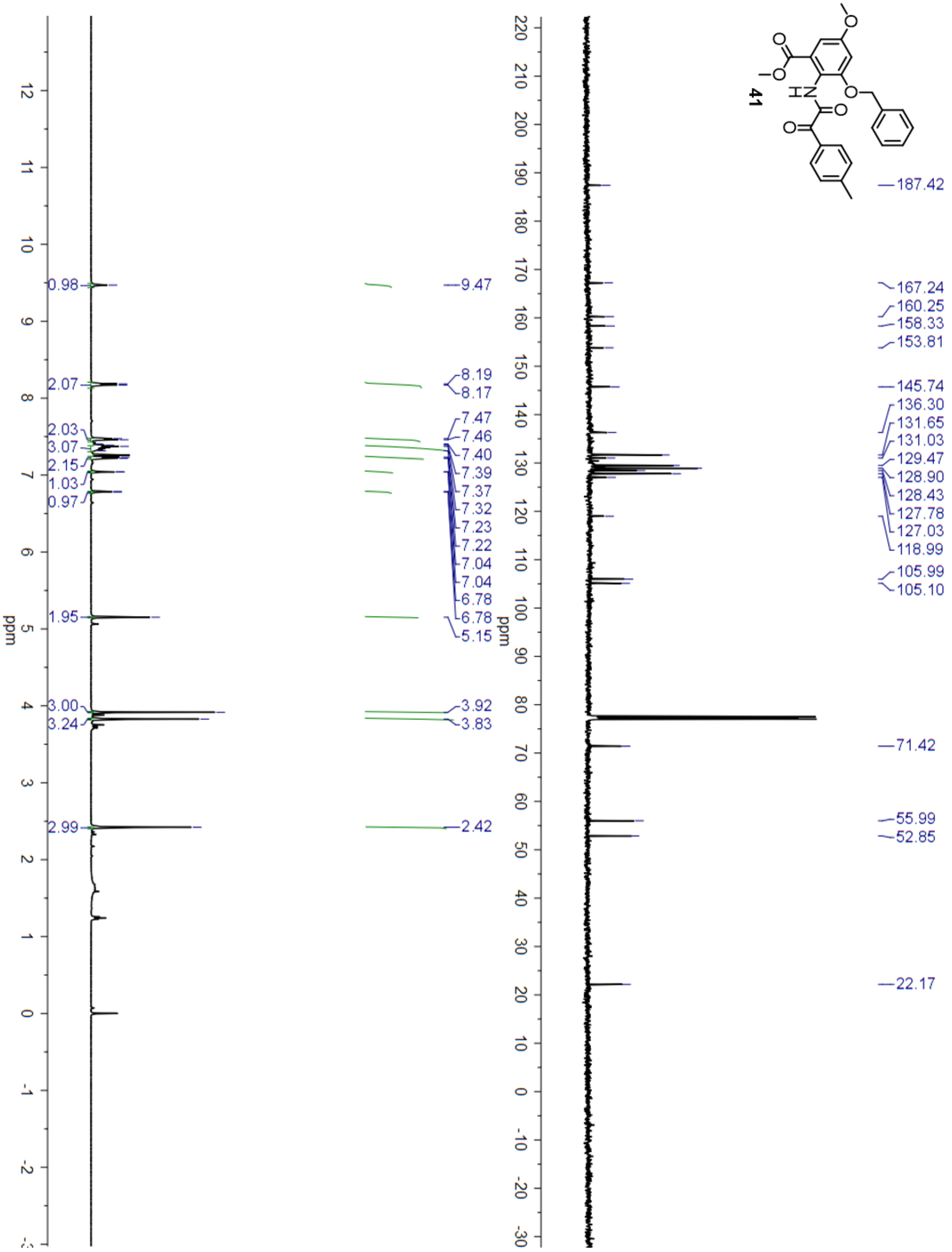
41
9.47
8.19
8.17
7.47
7.46
7.40
7.39
7.37
7.32
7.23
7.22
7.04
7.04
6.78
6.78
5.15
3.92
3.83
2.42
0.98
2.07
2.03
3.07
2.15
1.03
0.97
1.95
3.00
3.24
2.99
ppm
187.42
167.24
160.25
158.33
153.81
145.74
136.30
131.65
131.03
129.47
128.90
128.43
127.78
127.03
118.99
105.99
105.10
71.42
55.99
52.85
22.17
ppm

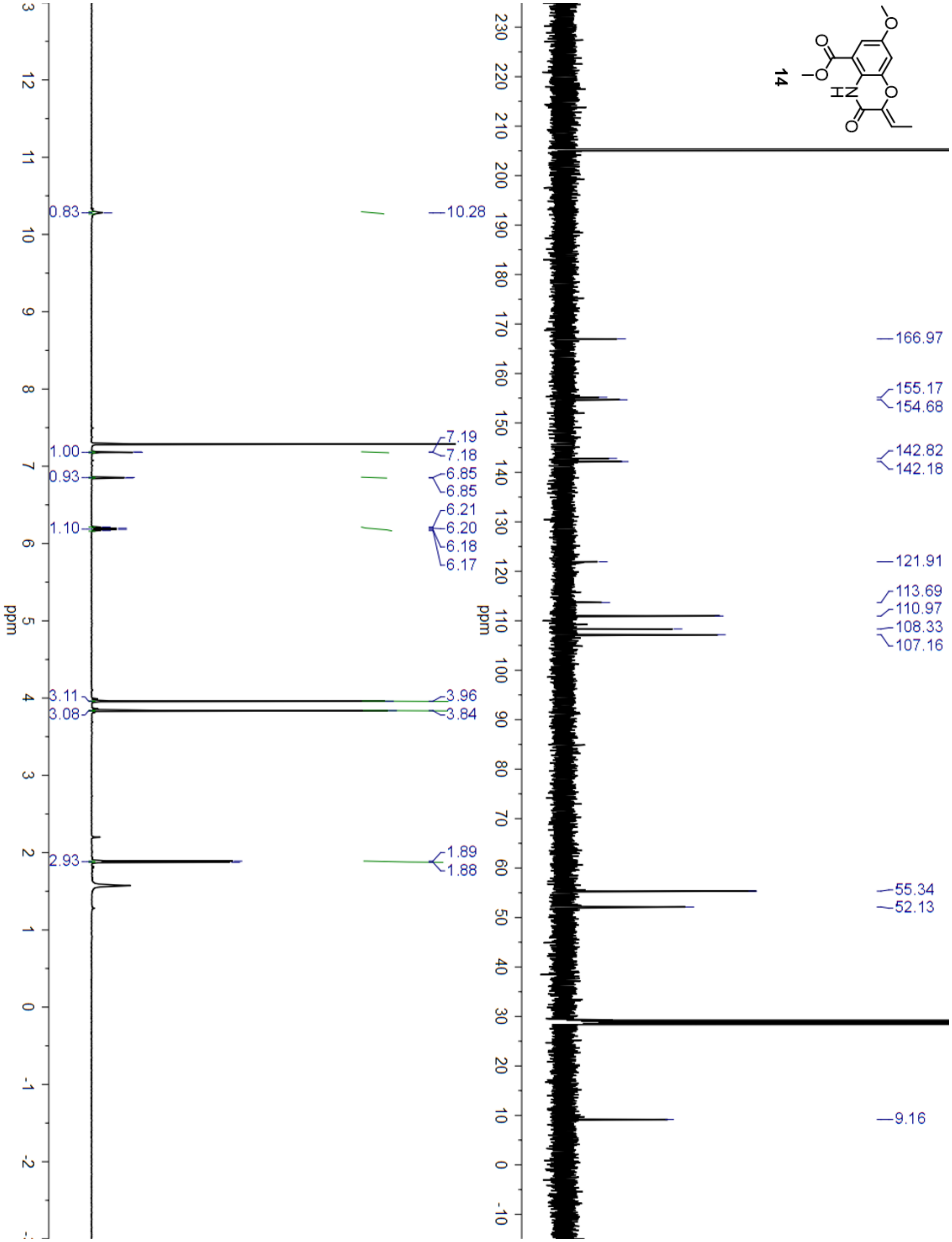
14
HN
0.83
1.00
0.93
1.10
3.11
3.08
2.93
10.28
7.19
7.18
6.85
6.85
6.21
6.20
6.18
6.17
3.96
3.84
1.89
1.88
ppm
166.97
155.17
154.68
142.82
142.18
121.91
113.69
110.97
108.33
107.16
55.34
52.13
9.16
ppm

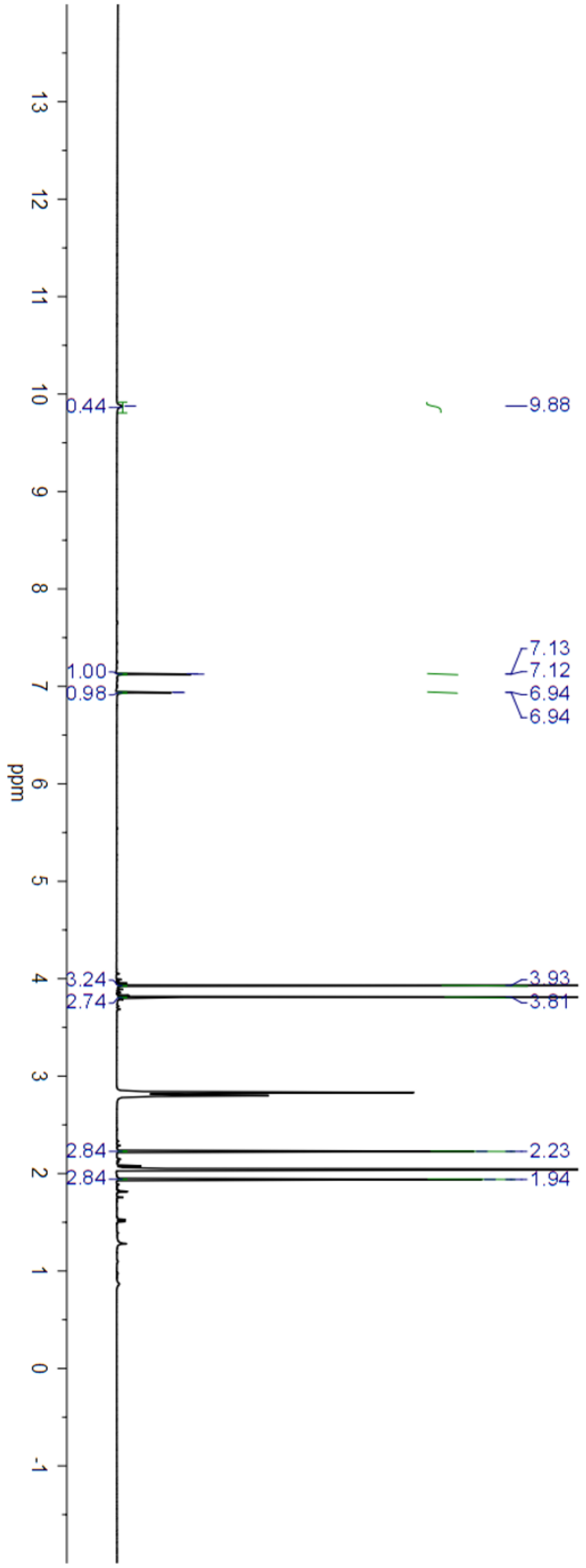

ppm
0.44
1.00
0.98
3.24
2.74
2.84
2.84
9.88
7.13
7.12
6.94
6.94
3.93
3.81
2.23
1.94

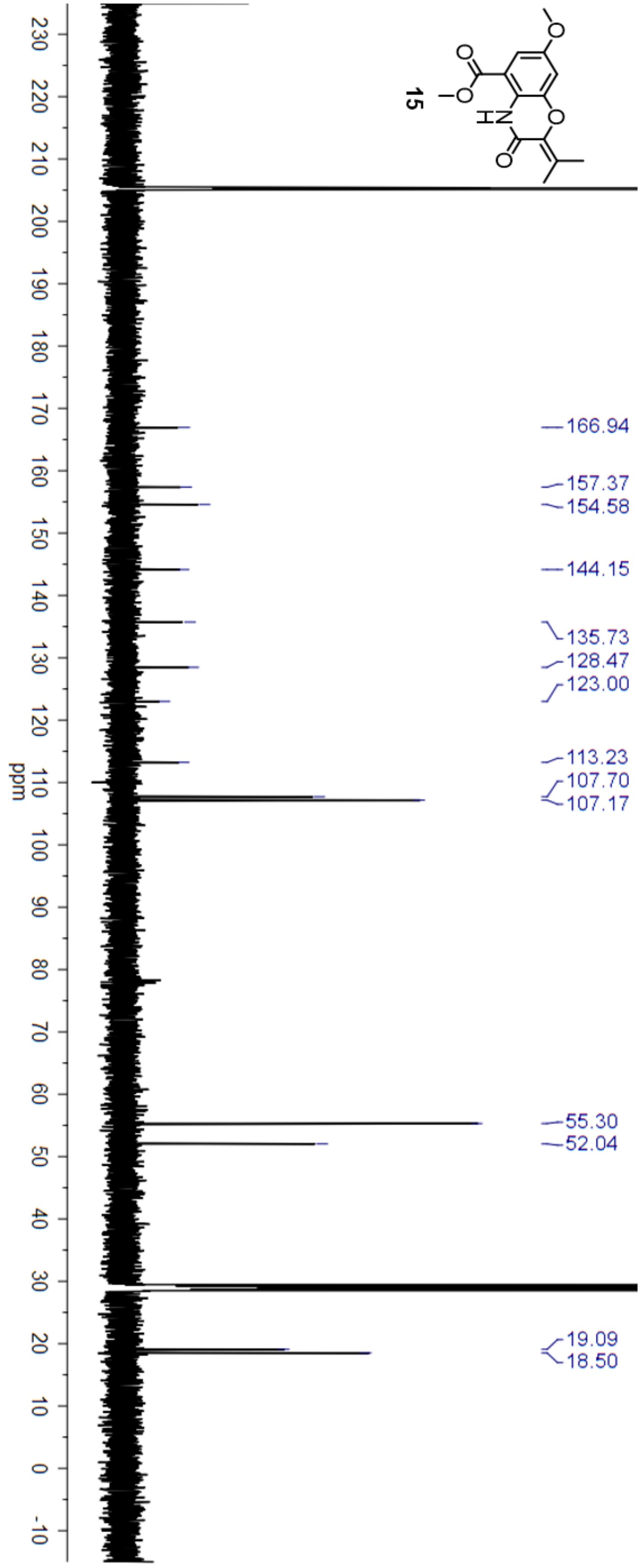

15
ppm
166.94
157.37
154.58
144.15
135.73
128.47
123.00
113.23
107.70
107.17
55.30
52.04
19.09
18.50

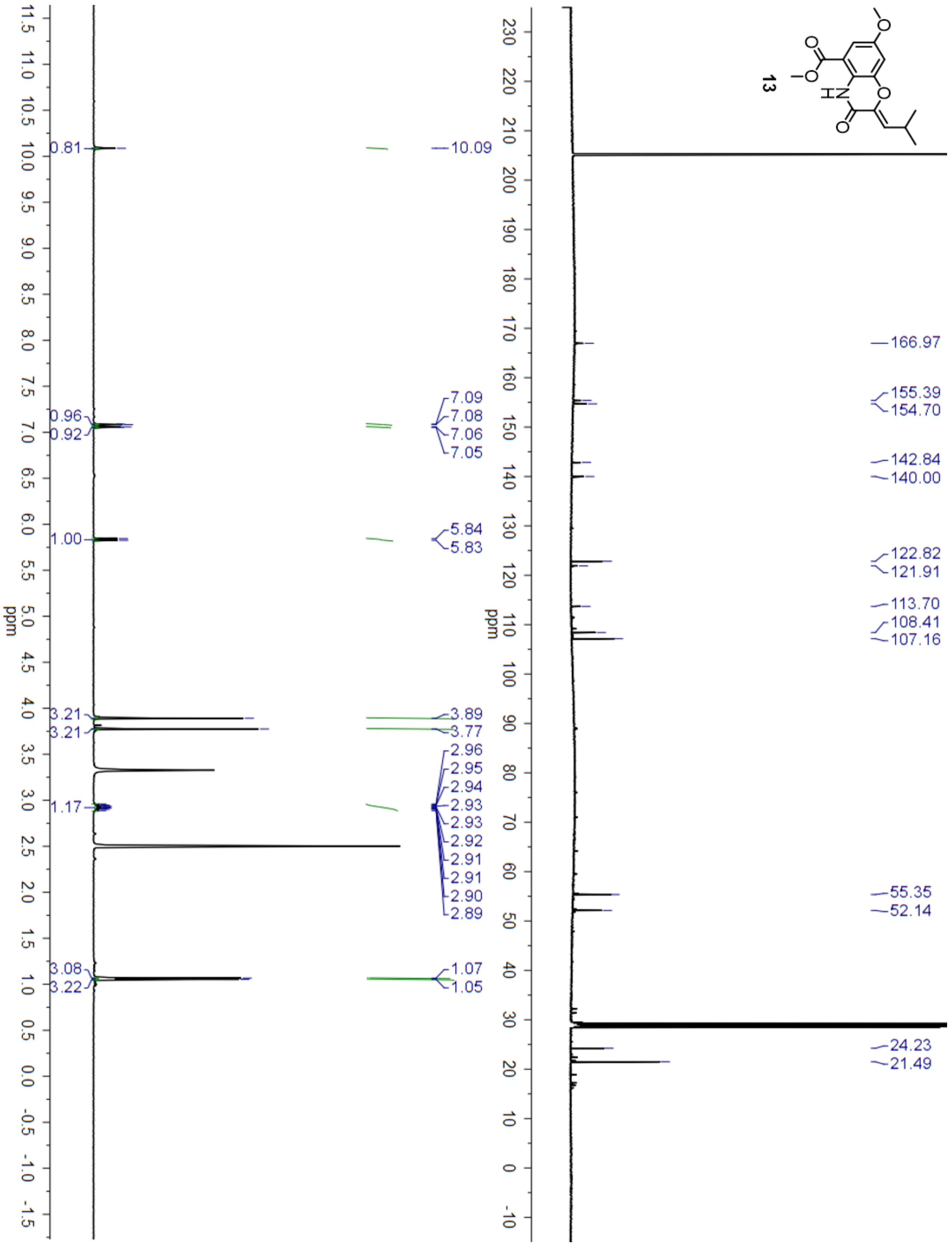
13
10.09
7.09
7.08
7.06
7.05
5.84
5.83
3.89
3.77
2.96
2.95
2.94
2.93
2.93
2.92
2.91
2.91
2.90
2.89
1.07
1.05
0.81
0.96
0.92
1.00
3.21
3.21
1.17
3.08
3.22
ppm
166.97
155.39
154.70
142.84
140.00
122.82
121.91
113.70
108.41
107.16
55.35
52.14
24.23
21.49
ppm

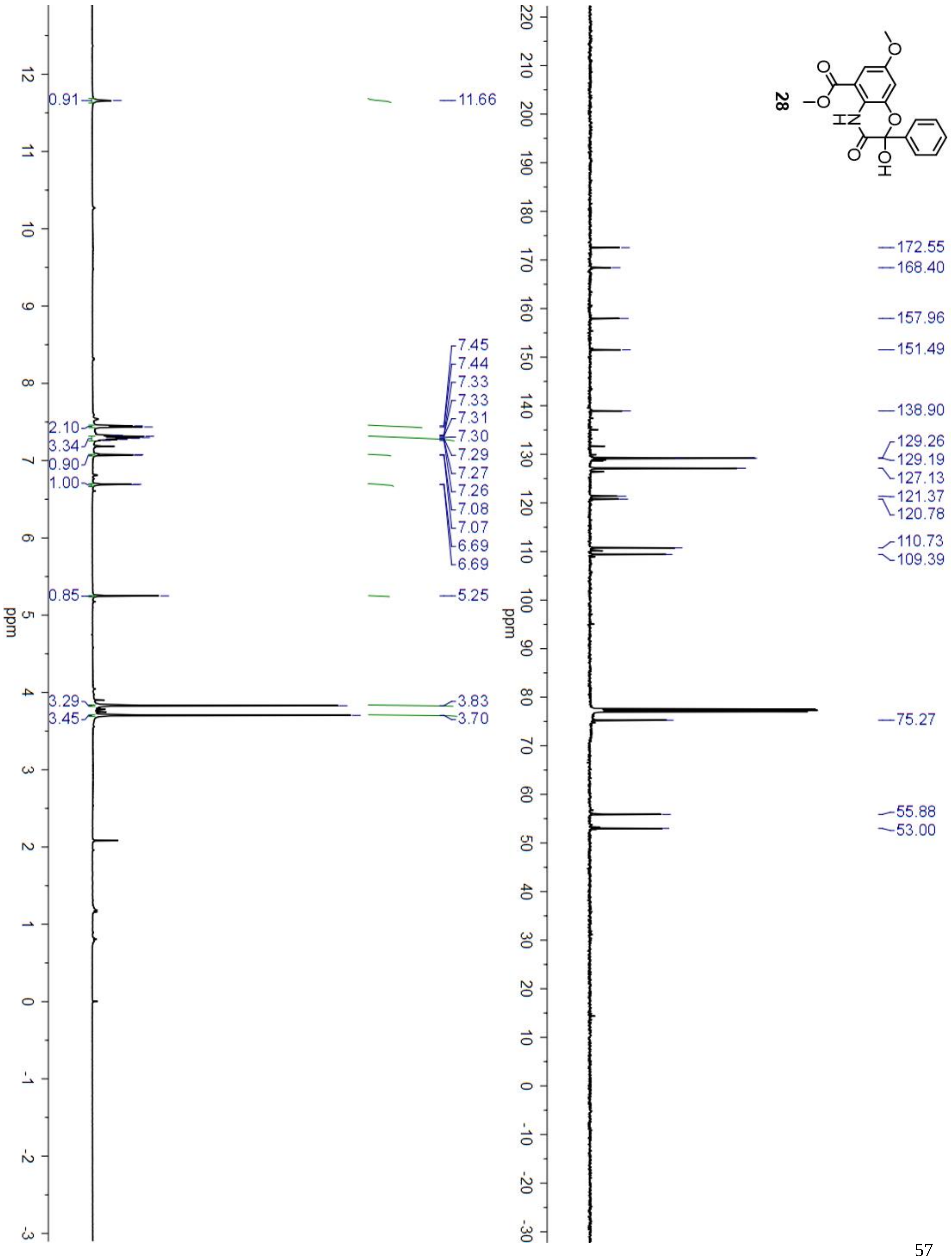
28
11.66
7.45
7.44
7.33
7.33
7.31
7.30
7.29
7.27
7.26
7.08
7.07
6.69
6.69
5.25
3.83
3.70
0.91
2.10
3.34
0.90
1.00
0.85
3.29
3.45
ppm
172.55
168.40
157.96
151.49
138.90
129.26
129.19
127.13
121.37
120.78
110.73
109.39
75.27
55.88
53.00
ppm

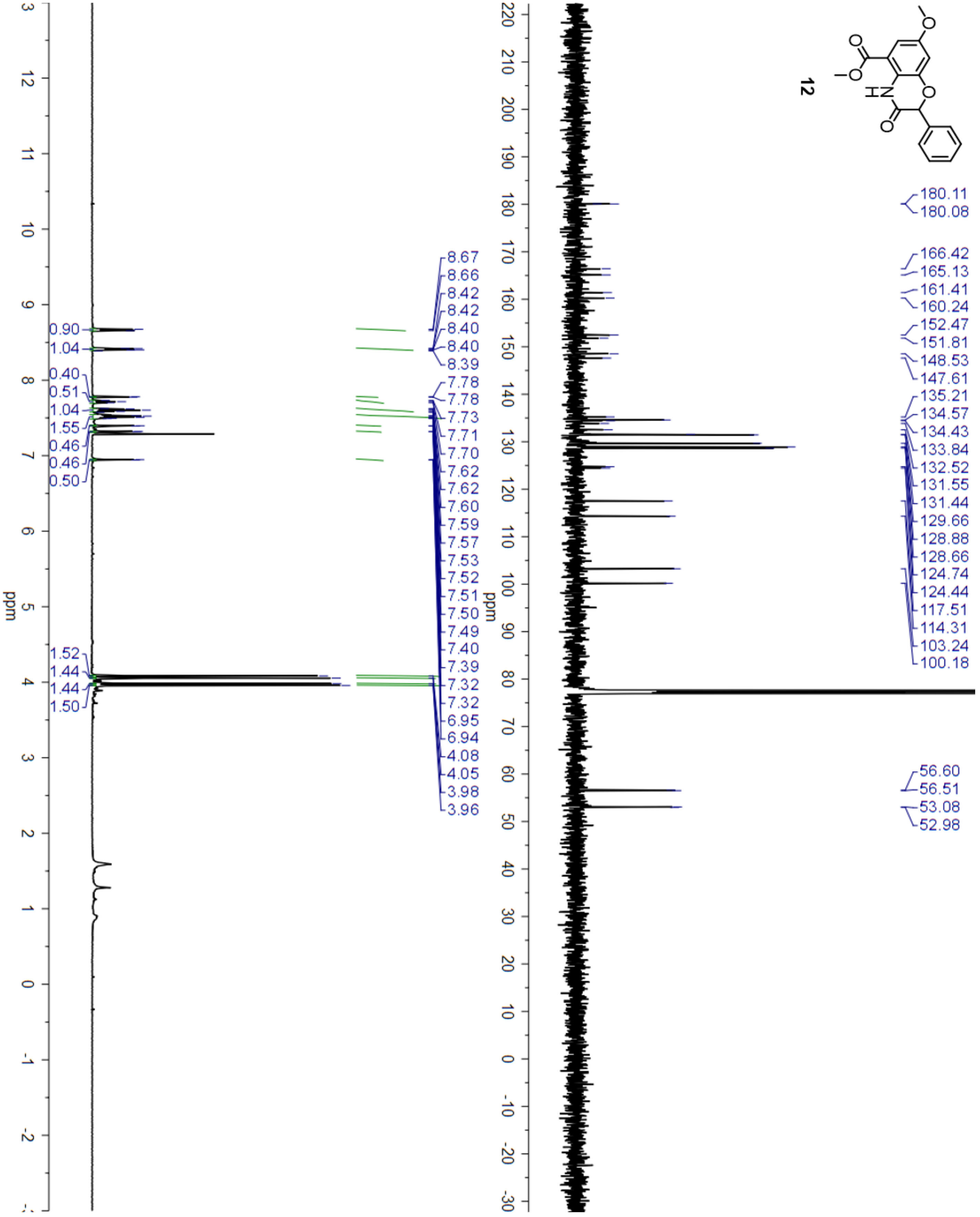
12
180.11
180.08
166.42
165.13
161.41
160.24
152.47
151.81
148.53
147.61
135.21
134.57
134.43
133.84
132.52
131.55
131.44
129.66
128.88
128.66
124.74
124.44
117.51
114.31
103.24
100.18
56.60
56.51
53.08
52.98
ppm
8.67
8.66
8.42
8.42
8.40
8.40
8.39
7.78
7.78
7.73
7.71
7.70
7.62
7.62
7.60
7.59
7.57
7.53
7.52
7.51
7.50
7.49
7.40
7.39
7.32
7.32
6.95
6.94
4.08
4.05
3.98
3.96
0.90
1.04
0.40
0.51
1.04
1.55
0.46
0.46
0.50
1.52
1.44
1.44
1.50
ppm

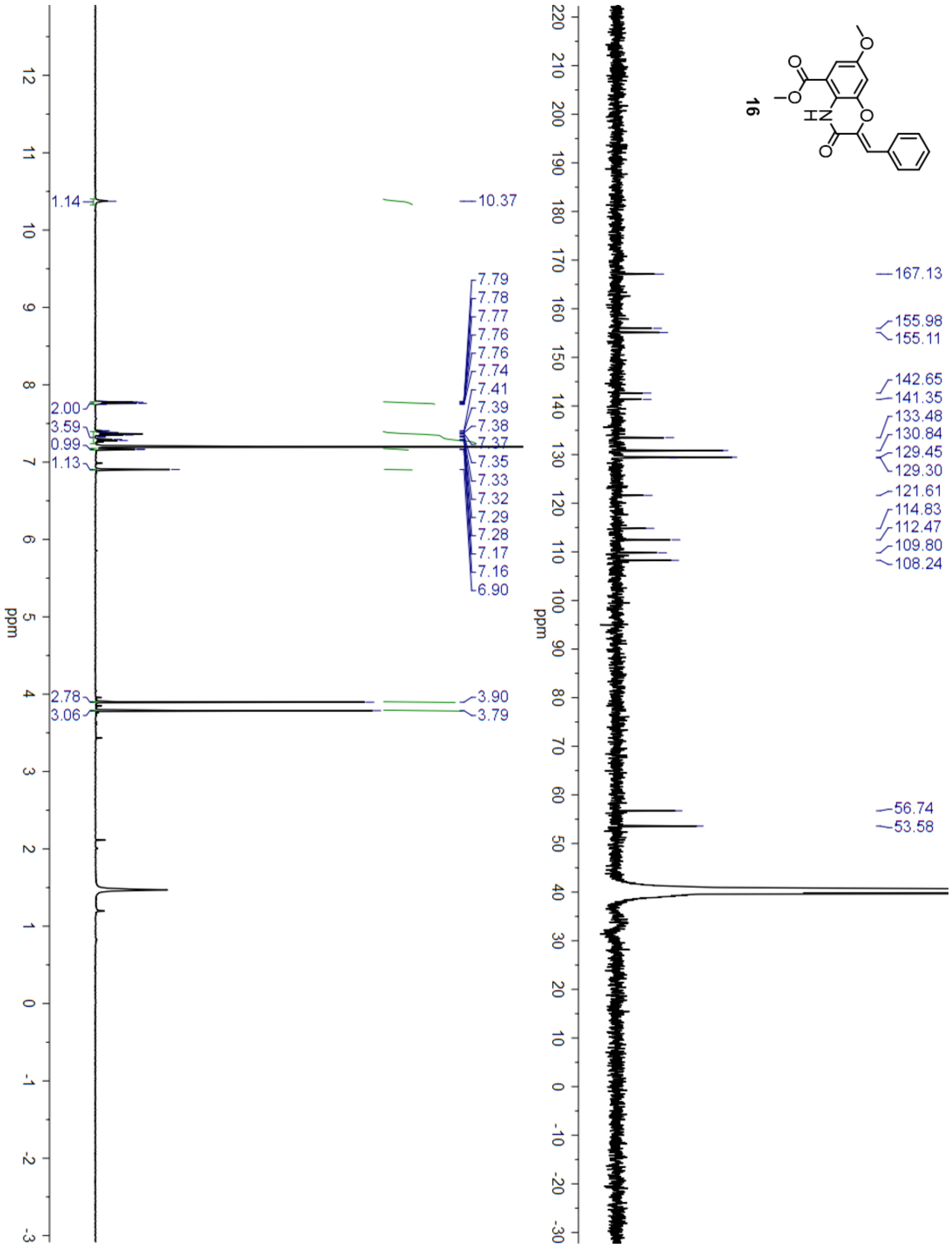
16
167.13
155.98
155.11
142.65
141.35
133.48
130.84
129.45
129.30
121.61
114.83
112.47
109.80
108.24
56.74
53.58
ppm
10.37
7.79
7.78
7.77
7.76
7.76
7.74
7.41
7.39
7.38
7.37
7.35
7.33
7.32
7.29
7.28
7.17
7.16
6.90
3.90
3.79
1.14
2.00
3.59
0.99
1.13
2.78
3.06
ppm

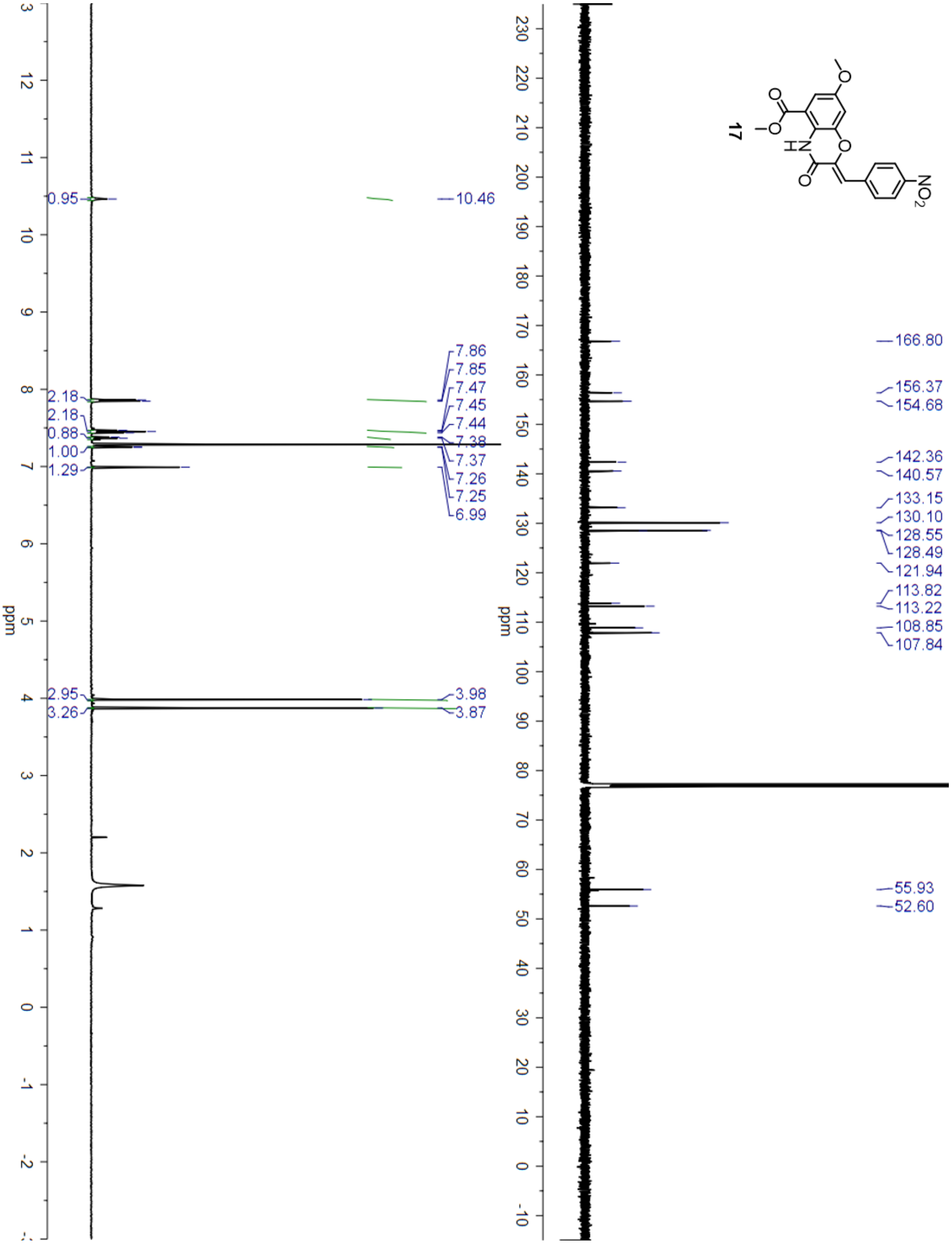
17
NO2
NH
10.46
7.86
7.85
7.47
7.45
7.44
7.38
7.37
7.26
7.25
6.99
3.98
3.87
0.95
2.18
2.18
0.88
1.00
1.29
2.95
3.26
ppm
166.80
156.37
154.68
142.36
140.57
133.15
130.10
128.55
128.49
121.94
113.82
113.22
108.85
107.84
55.93
52.60
ppm

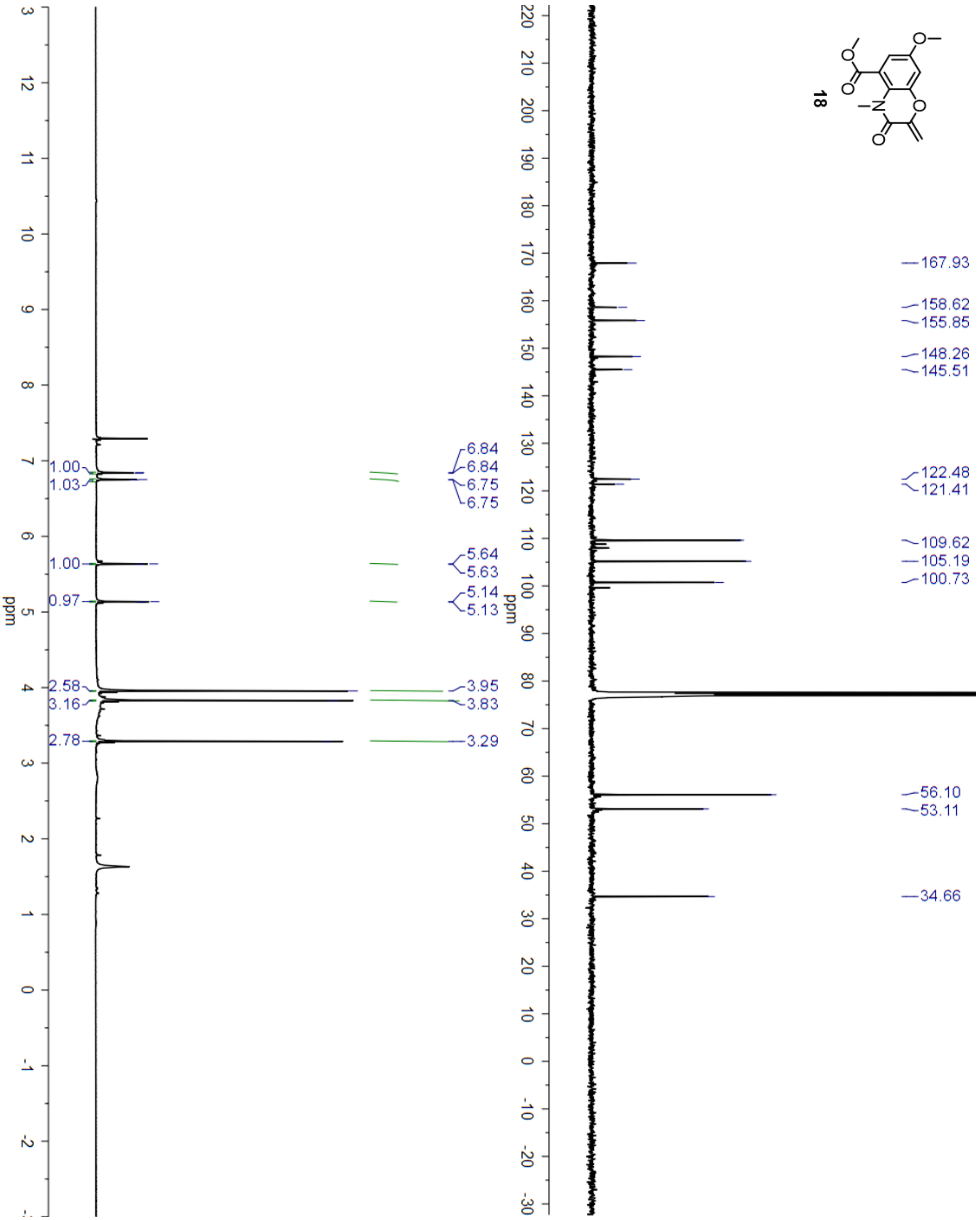
18
167.93
158.62
155.85
148.26
145.51
122.48
121.41
109.62
105.19
100.73
56.10
53.11
34.66
6.84
6.84
6.75
6.75
5.64
5.63
5.14
5.13
3.95
3.83
3.29
1.00
1.03
1.00
0.97
2.58
3.16
2.78
ppm
ppm

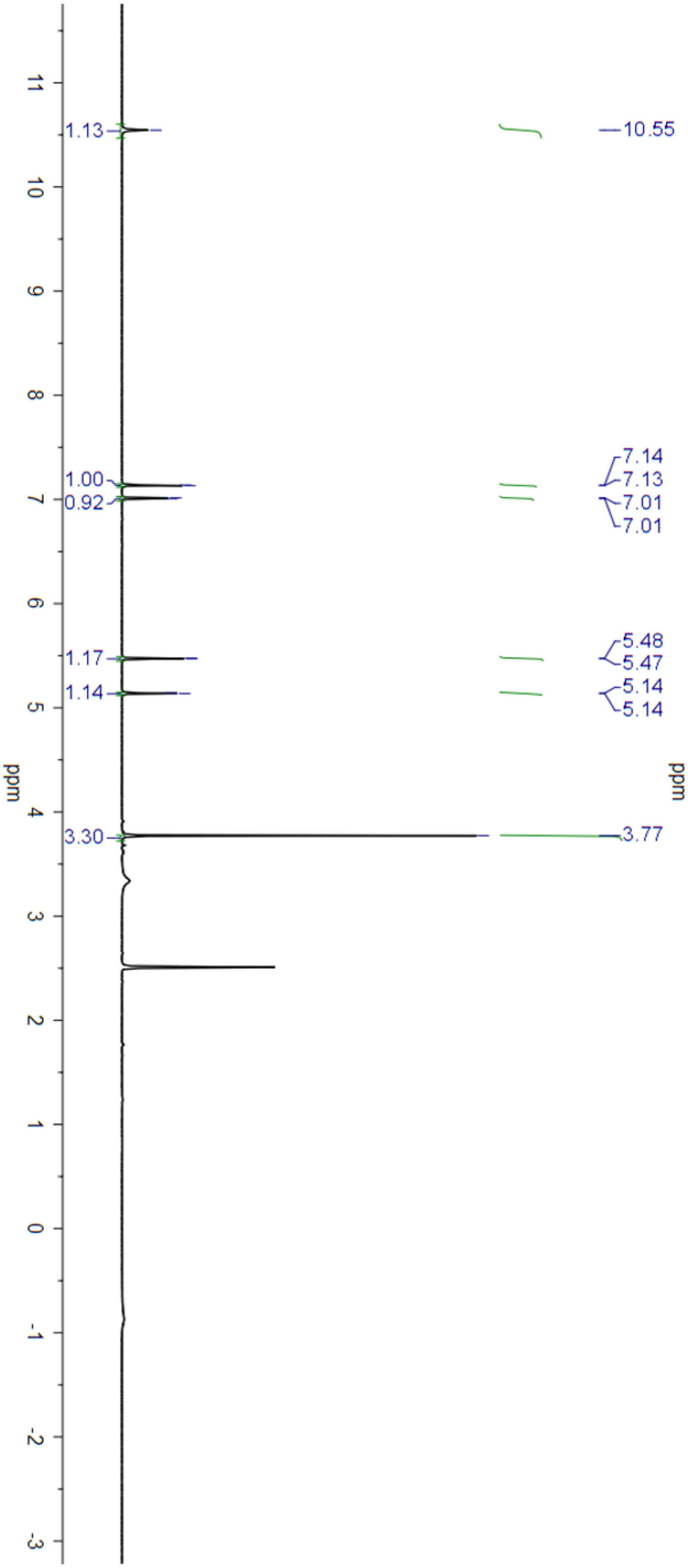
1.13
1.00
0.92
1.17
1.14
3.30
10.55
7.14
7.13
7.01
7.01
5.48
5.47
5.14
5.14
3.77
ppm

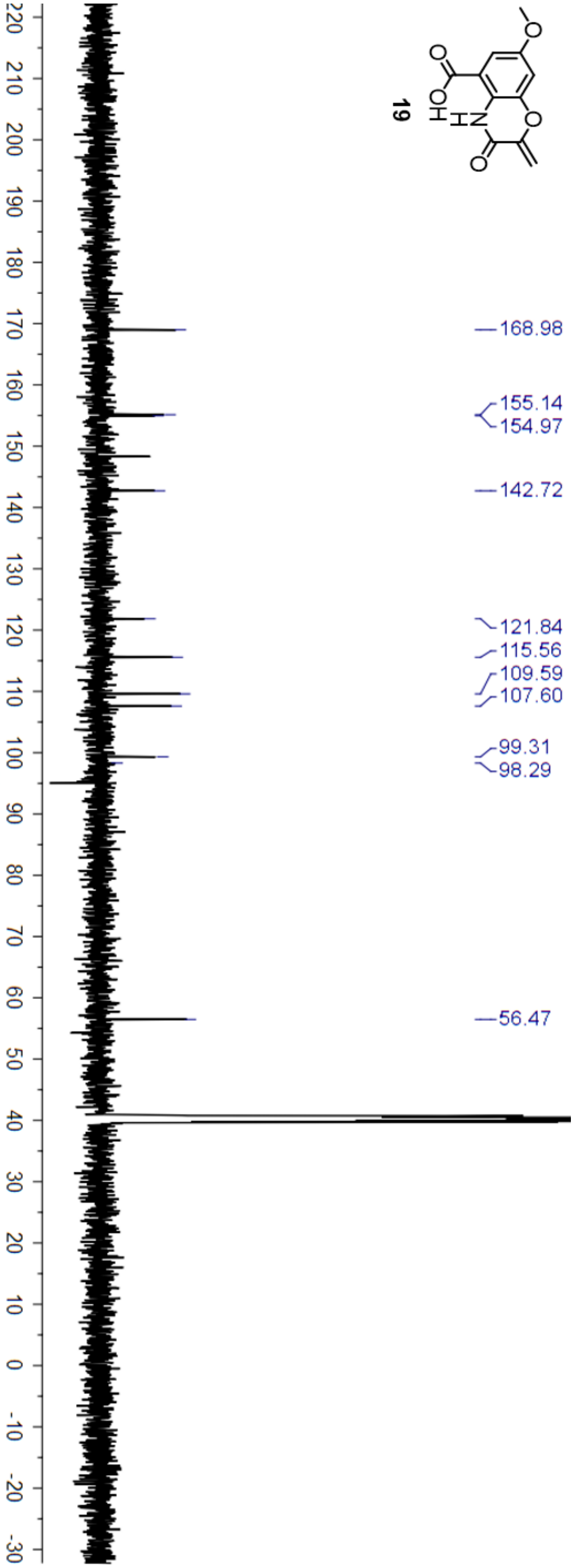
19
168.98
155.14
154.97
142.72
121.84
115.56
109.59
107.60
99.31
98.29
56.47
ppm

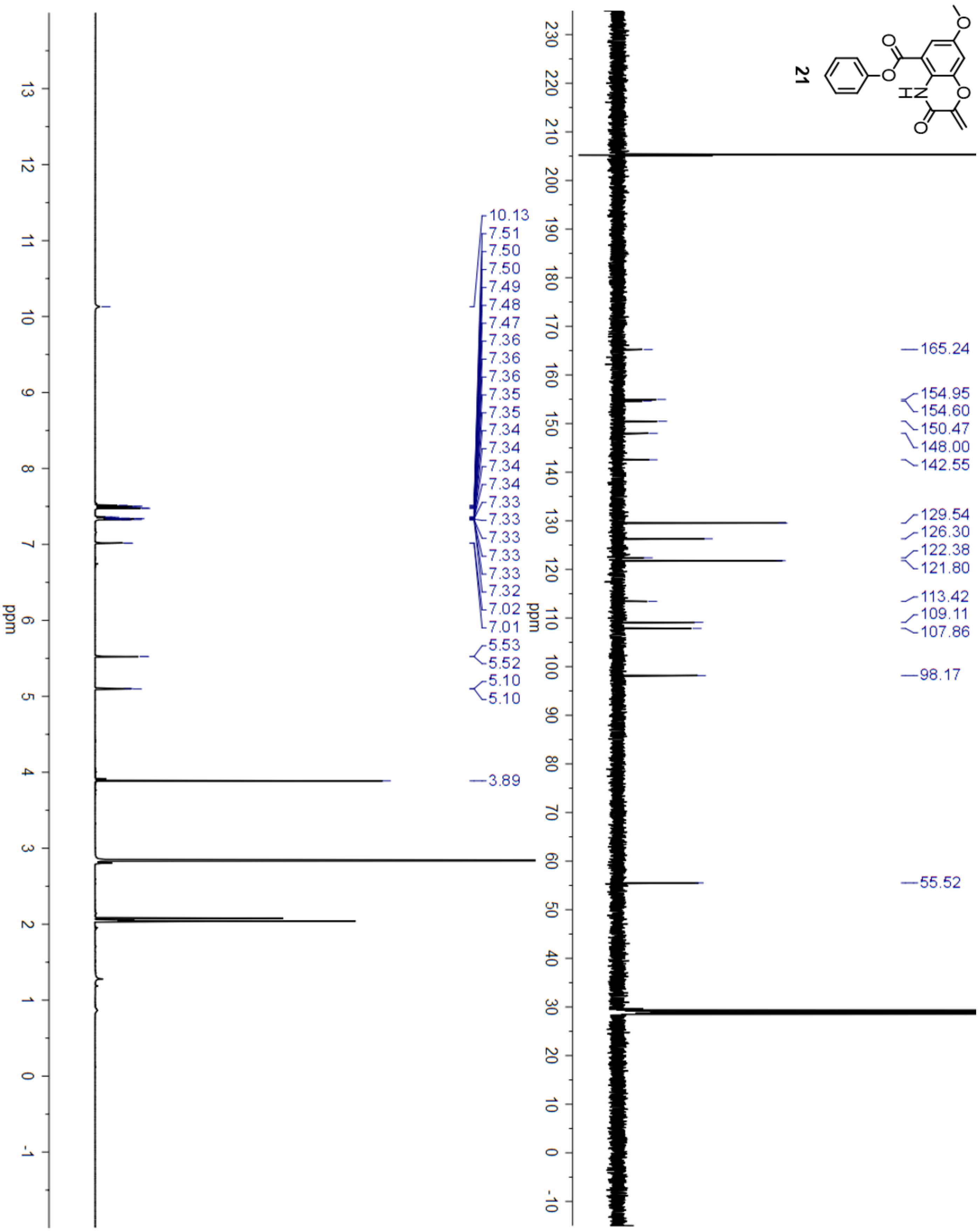
21
10.13
7.51
7.50
7.50
7.49
7.48
7.47
7.36
7.36
7.36
7.35
7.35
7.34
7.34
7.34
7.34
7.33
7.33
7.33
7.33
7.33
7.32
7.02
7.01
5.53
5.52
5.10
5.10
3.89
ppm
165.24
154.95
154.60
150.47
148.00
142.55
129.54
126.30
122.38
121.80
113.42
109.11
107.86
98.17
55.52
ppm

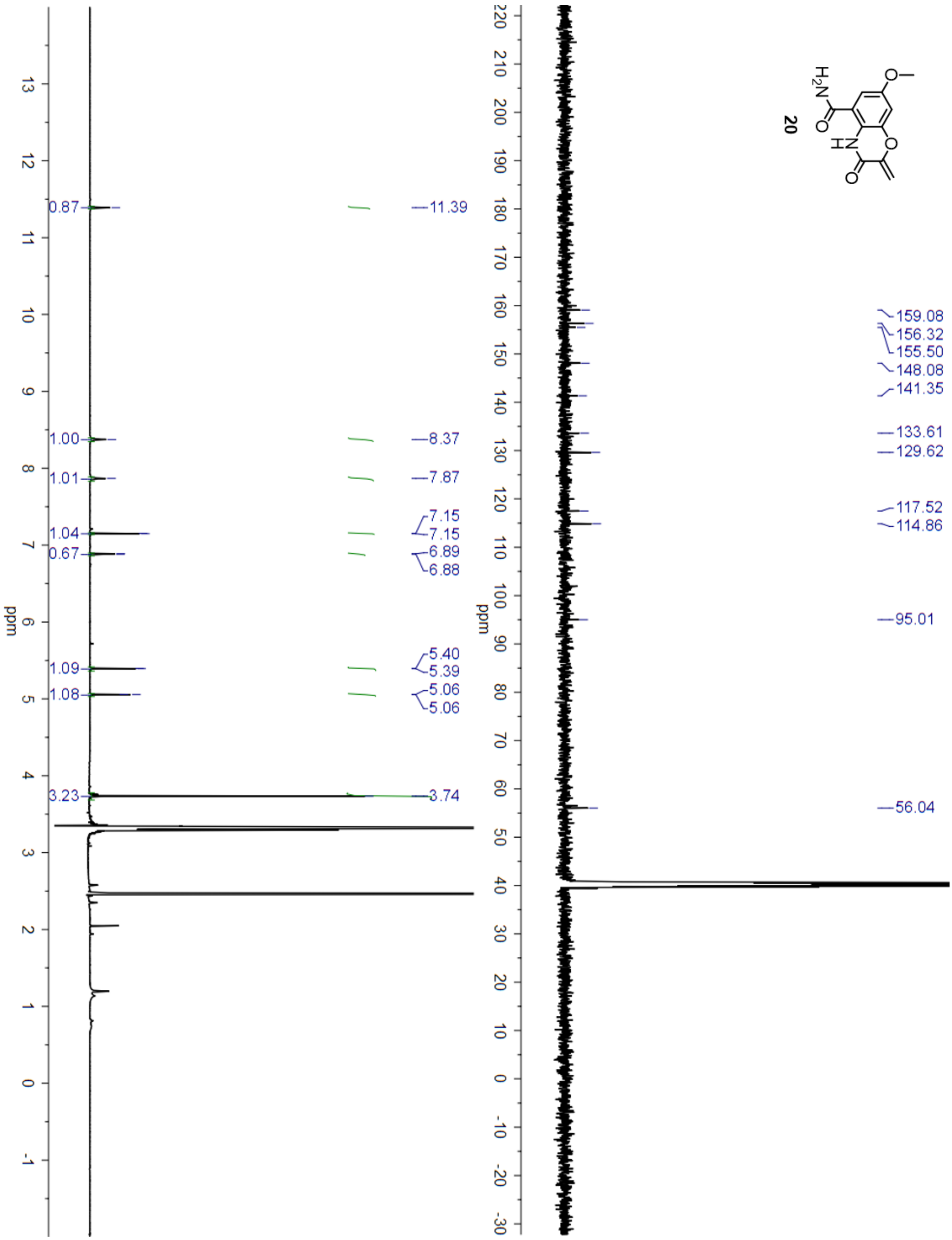
20
H_2N
159.08
156.32
155.50
148.08
141.35
133.61
129.62
117.52
114.86
95.01
56.04
ppm
11.39
8.37
7.87
7.15
7.15
6.89
6.88
5.40
5.39
5.06
5.06
3.74
0.87
1.00
1.01
1.04
0.67
1.09
1.08
3.23

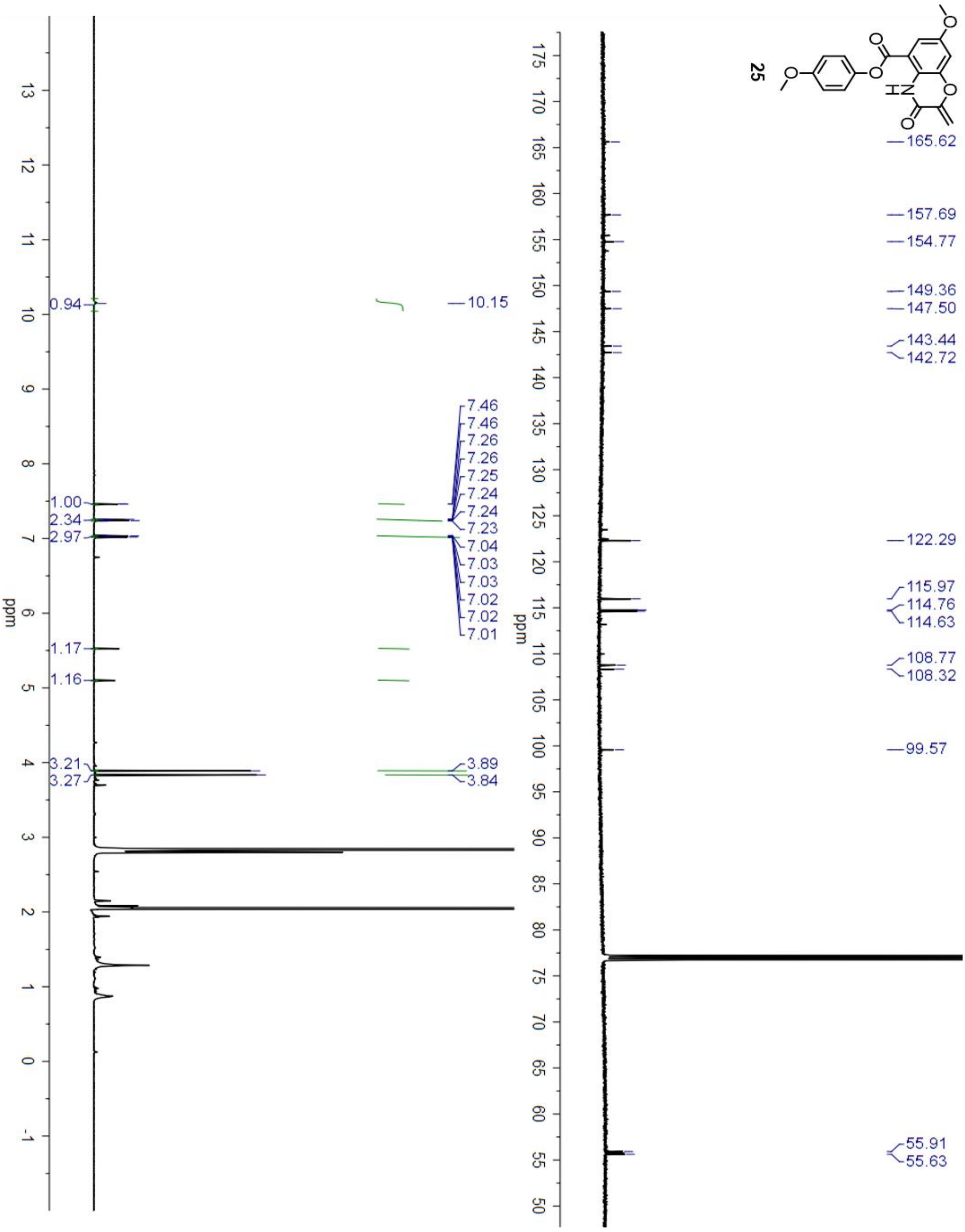
25
165.62
157.69
154.77
149.36
147.50
143.44
142.72
122.29
115.97
114.76
114.63
108.77
108.32
99.57
55.91
55.63
ppm
10.15
7.46
7.46
7.26
7.26
7.25
7.24
7.24
7.23
7.04
7.03
7.03
7.02
7.02
7.01
3.89
3.84
0.94
1.00
2.34
2.97
1.17
1.16
3.21
3.27
ppm

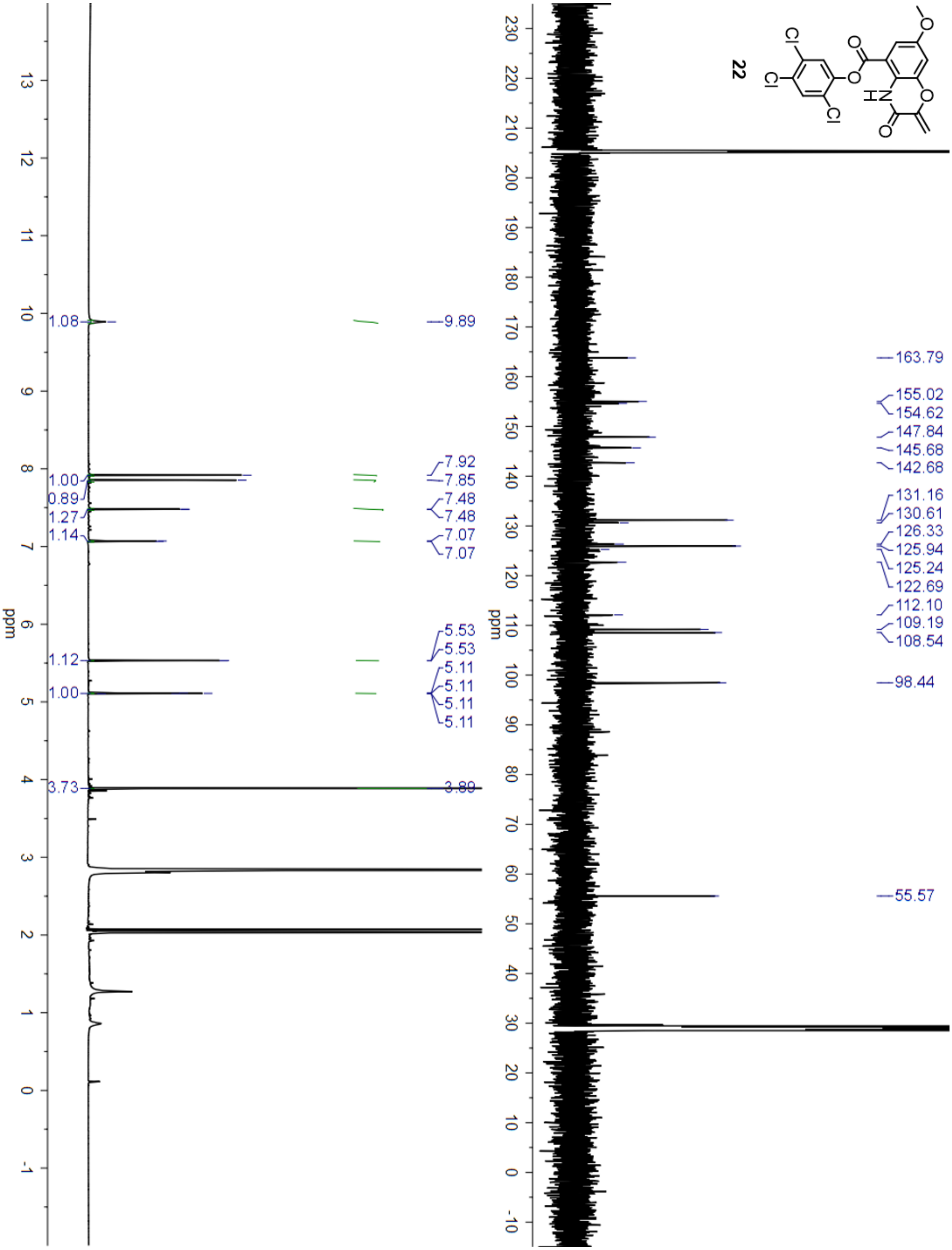
22
9.89
7.92
7.85
7.48
7.48
7.07
7.07
5.53
5.53
5.11
5.11
5.11
5.11
3.89
1.08
1.00
0.89
1.27
1.14
1.12
1.00
3.73
ppm
163.79
155.02
154.62
147.84
145.68
142.68
131.16
130.61
126.33
125.94
125.24
122.69
112.10
109.19
108.54
98.44
55.57
ppm

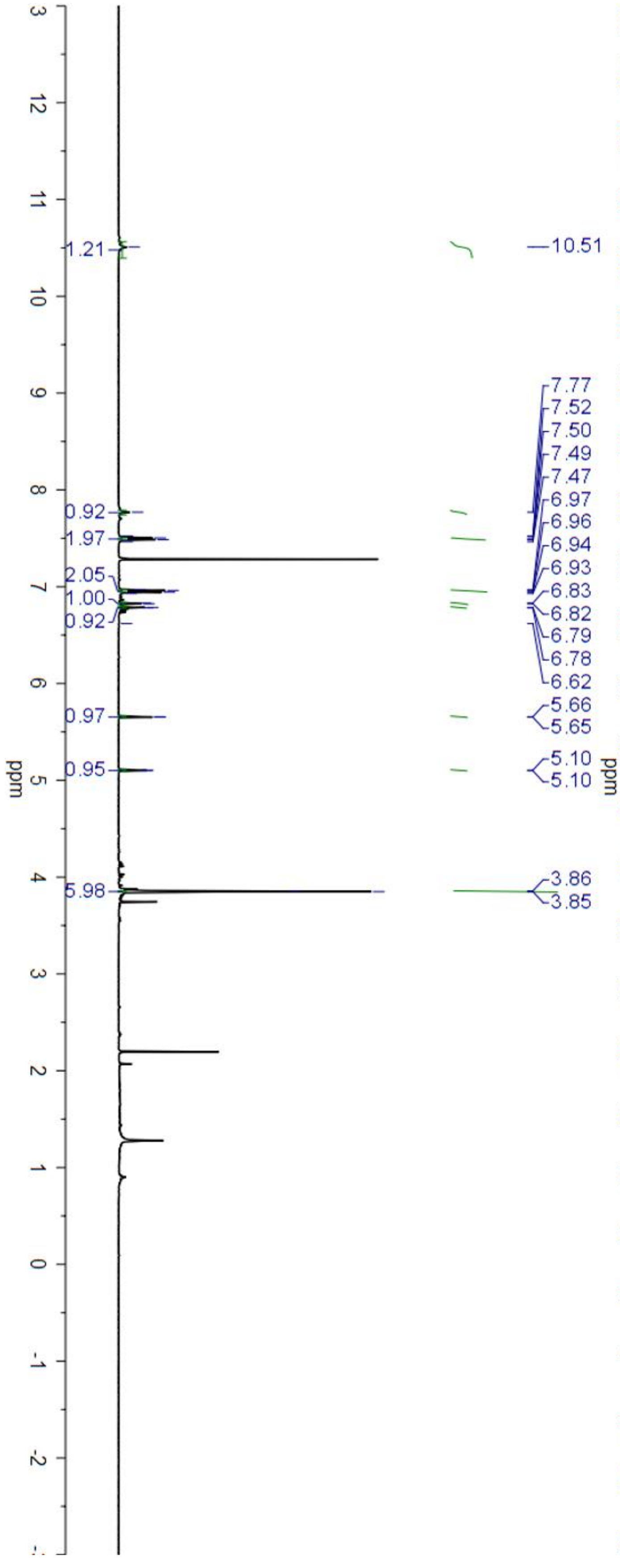

10.51
7.77
7.52
7.50
7.49
7.47
6.97
6.96
6.94
6.93
6.83
6.82
6.79
6.78
6.62
5.66
5.65
5.10
5.10
3.86
3.85
1.21
0.92
1.97
2.05
1.00
0.92
0.97
0.95
5.98
ppm

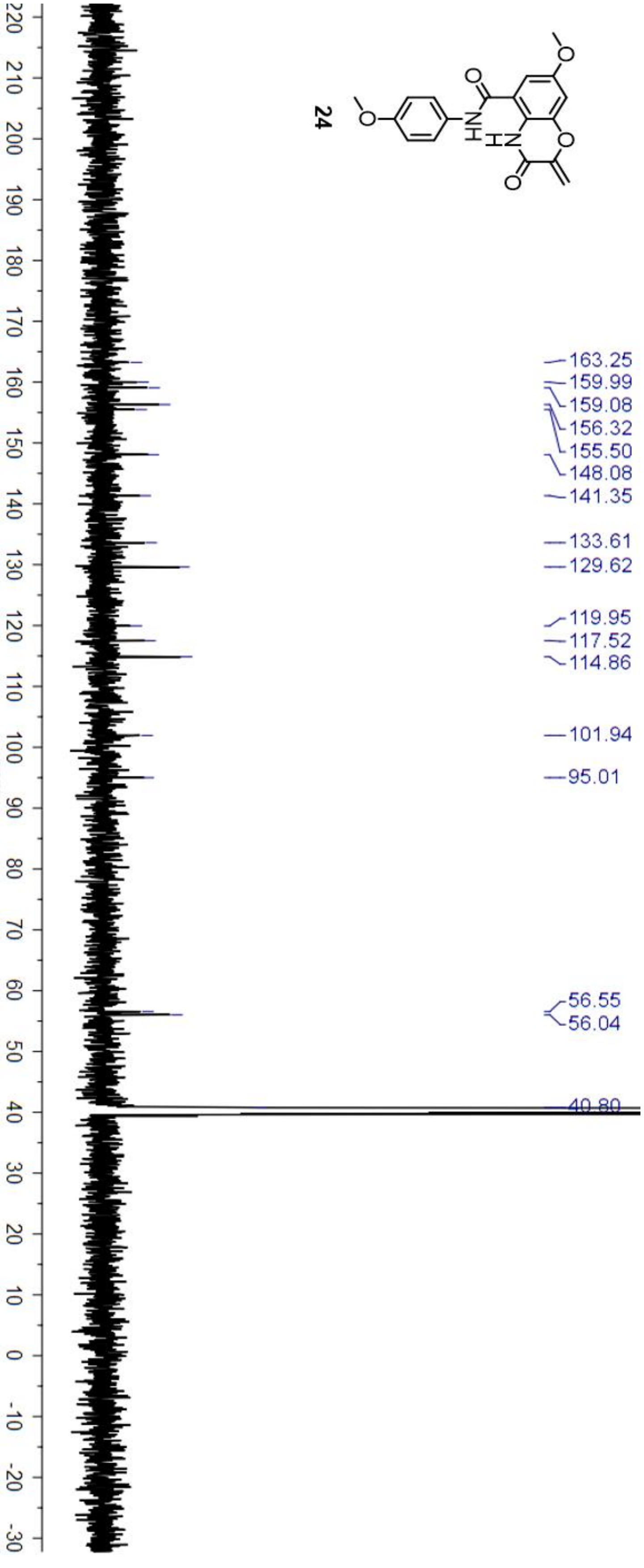

24
163.25
159.99
159.08
156.32
155.50
148.08
141.35
133.61
129.62
119.95
117.52
114.86
101.94
95.01
56.55
56.04
40.80
ppm

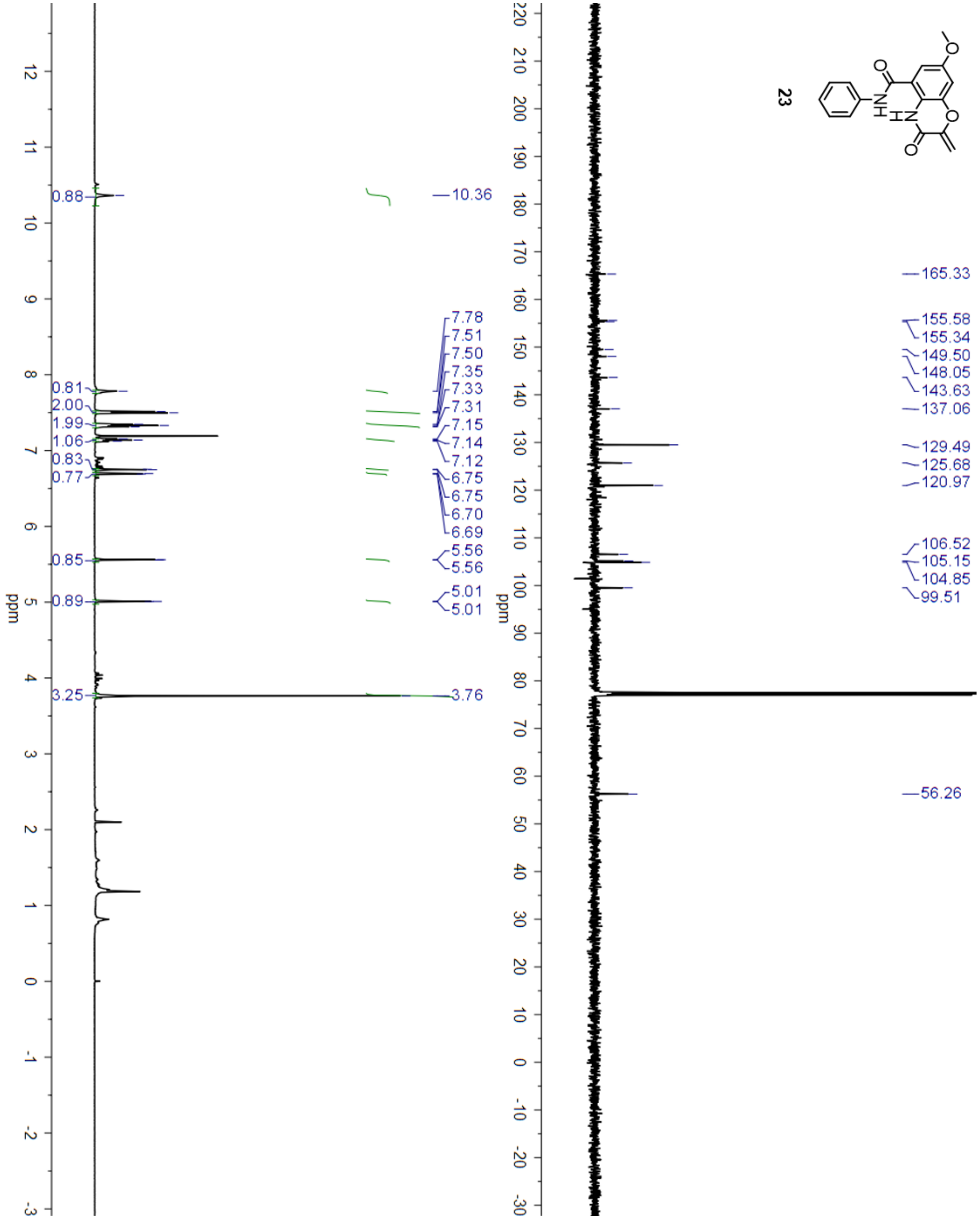
23
10.36
7.78
7.51
7.50
7.35
7.33
7.31
7.15
7.14
7.12
6.75
6.75
6.70
6.69
5.56
5.56
5.01
5.01
3.76
0.88
0.81
2.00
1.99
1.06
0.83
0.77
0.85
0.89
3.25
ppm
165.33
155.58
155.34
149.50
148.05
143.63
137.06
129.49
125.68
120.97
106.52
105.15
104.85
99.51
56.26
ppm

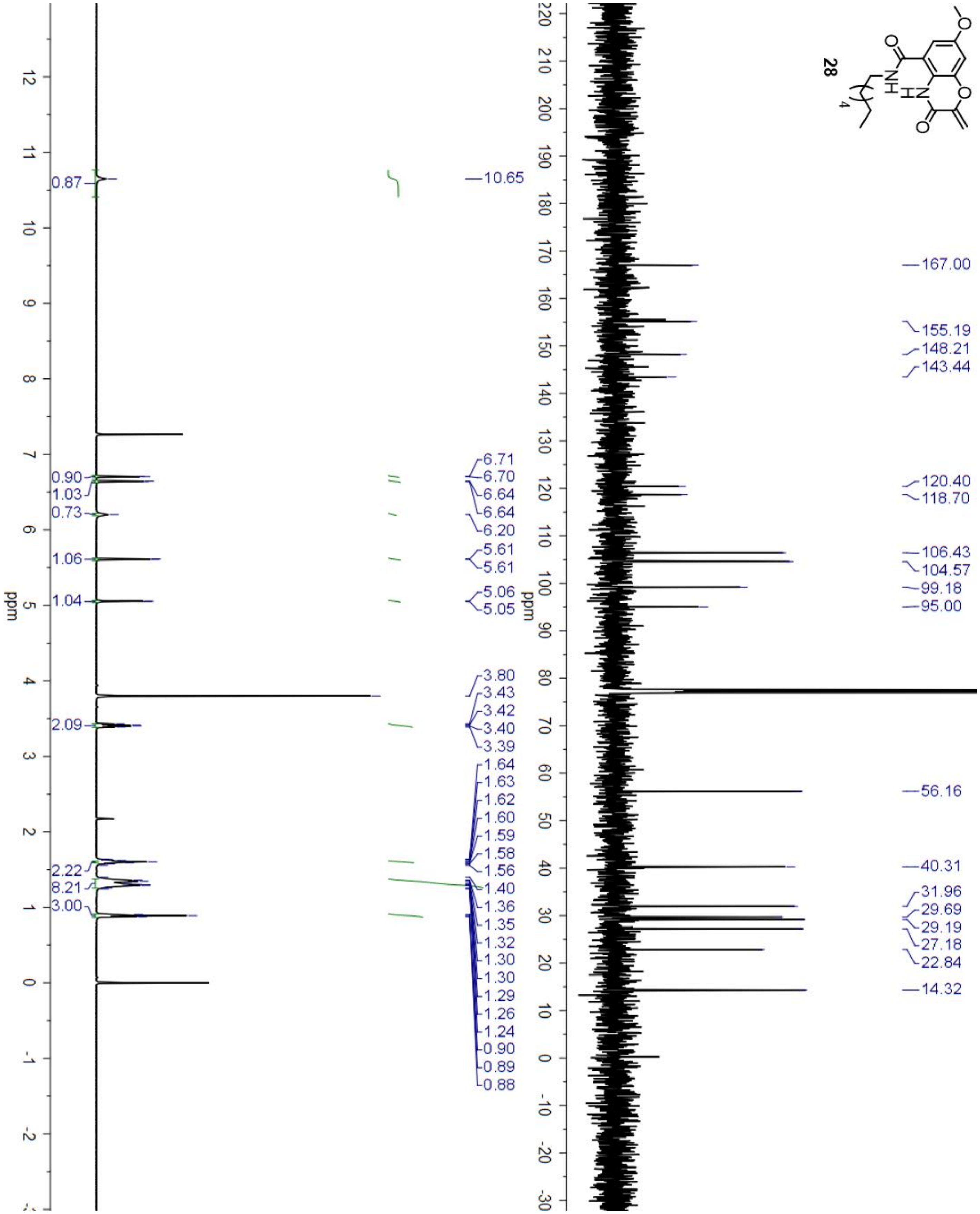
28
10.65
6.71
6.70
6.64
6.64
6.20
5.61
5.61
5.06
5.05
3.80
3.43
3.42
3.40
3.39
1.64
1.63
1.62
1.60
1.59
1.58
1.56
1.40
1.36
1.35
1.32
1.30
1.30
1.29
1.26
1.24
0.90
0.89
0.88
0.87
0.90
1.03
0.73
1.06
1.04
2.09
2.22
8.21
3.00
ppm
167.00
155.19
148.21
143.44
120.40
118.70
106.43
104.57
99.18
95.00
56.16
40.31
31.96
29.69
29.19
27.18
22.84
14.32
ppm

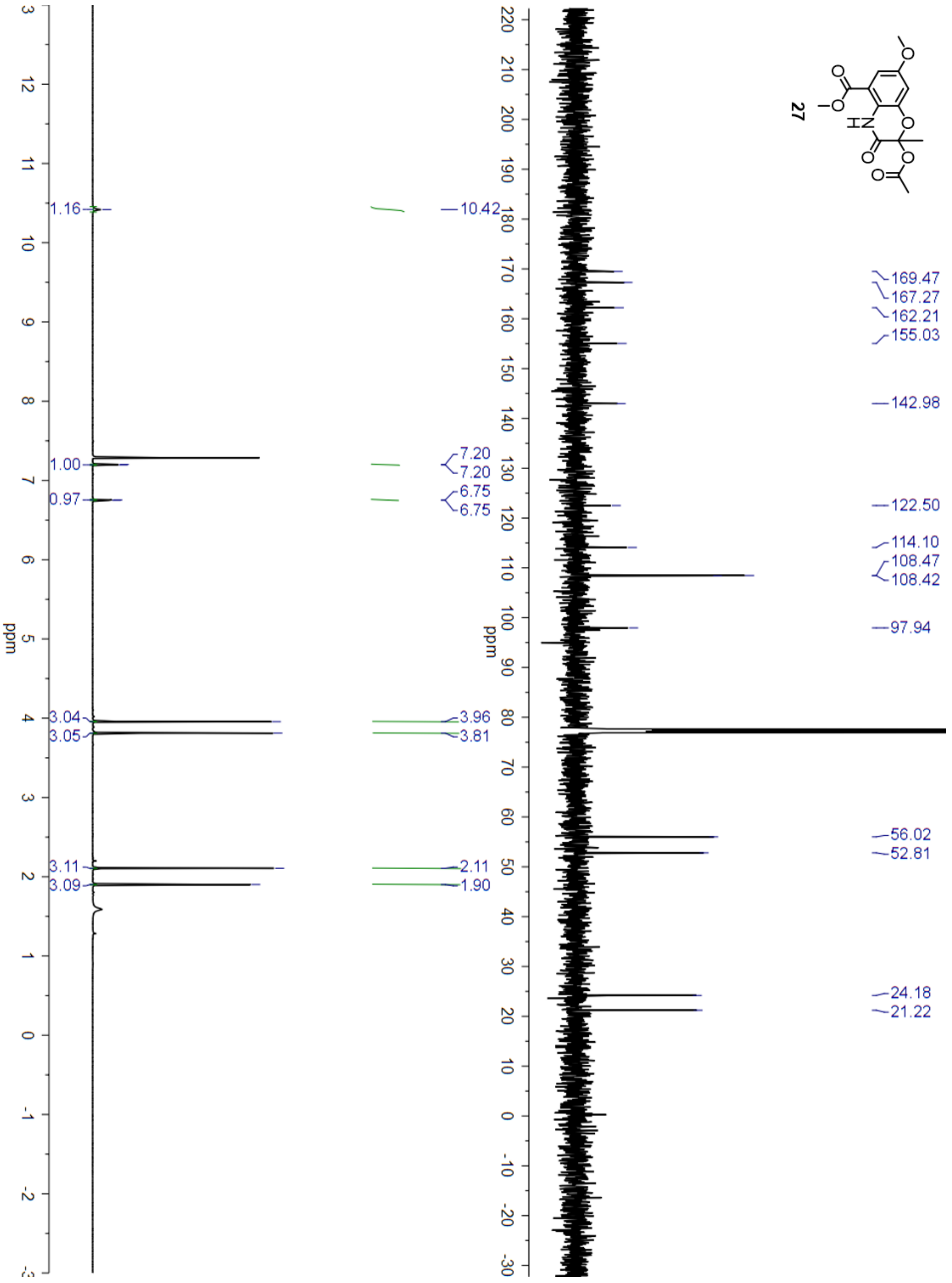
27
ppm
1.16
1.00
0.97
3.04
3.05
3.11
3.09
10.42
7.20
7.20
6.75
6.75
3.96
3.81
2.11
1.90
169.47
167.27
162.21
155.03
142.98
122.50
114.10
108.47
108.42
97.94
56.02
52.81
24.18
21.22

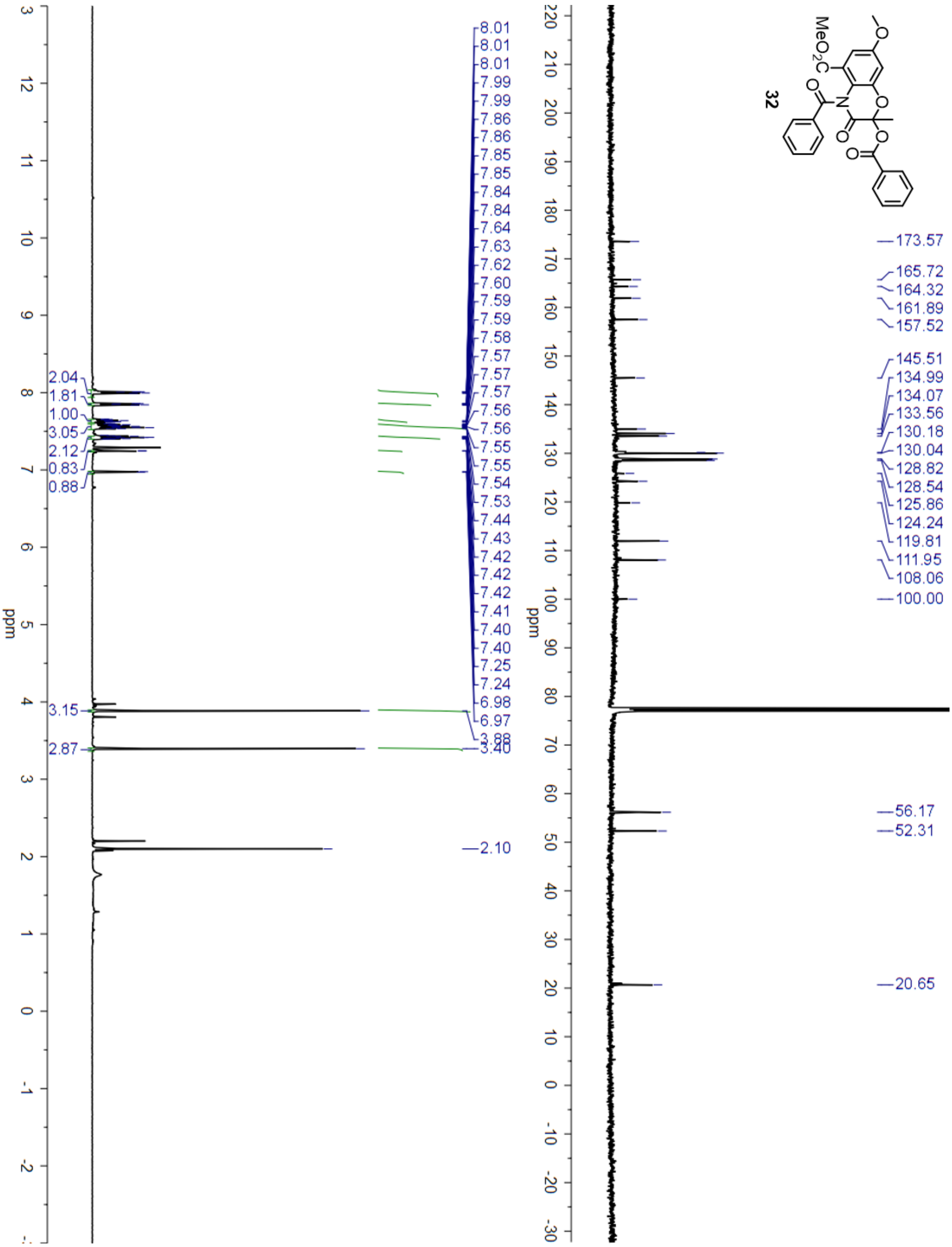
MeO2C
N
O
O
O
O
O
O
32
173.57
165.72
164.32
161.89
157.52
145.51
134.99
134.07
133.56
130.18
130.04
128.82
128.54
125.86
124.24
119.81
111.95
108.06
100.00
56.17
52.31
20.65
ppm
8.01
8.01
8.01
7.99
7.99
7.86
7.86
7.85
7.85
7.84
7.84
7.64
7.63
7.62
7.60
7.59
7.59
7.58
7.57
7.57
7.57
7.56
7.56
7.55
7.55
7.54
7.53
7.44
7.43
7.42
7.42
7.42
7.41
7.40
7.40
7.25
7.24
6.98
6.97
3.88
3.40
2.10
2.04
1.81
1.00
3.05
2.12
0.83
0.88
3.15
2.87
ppm

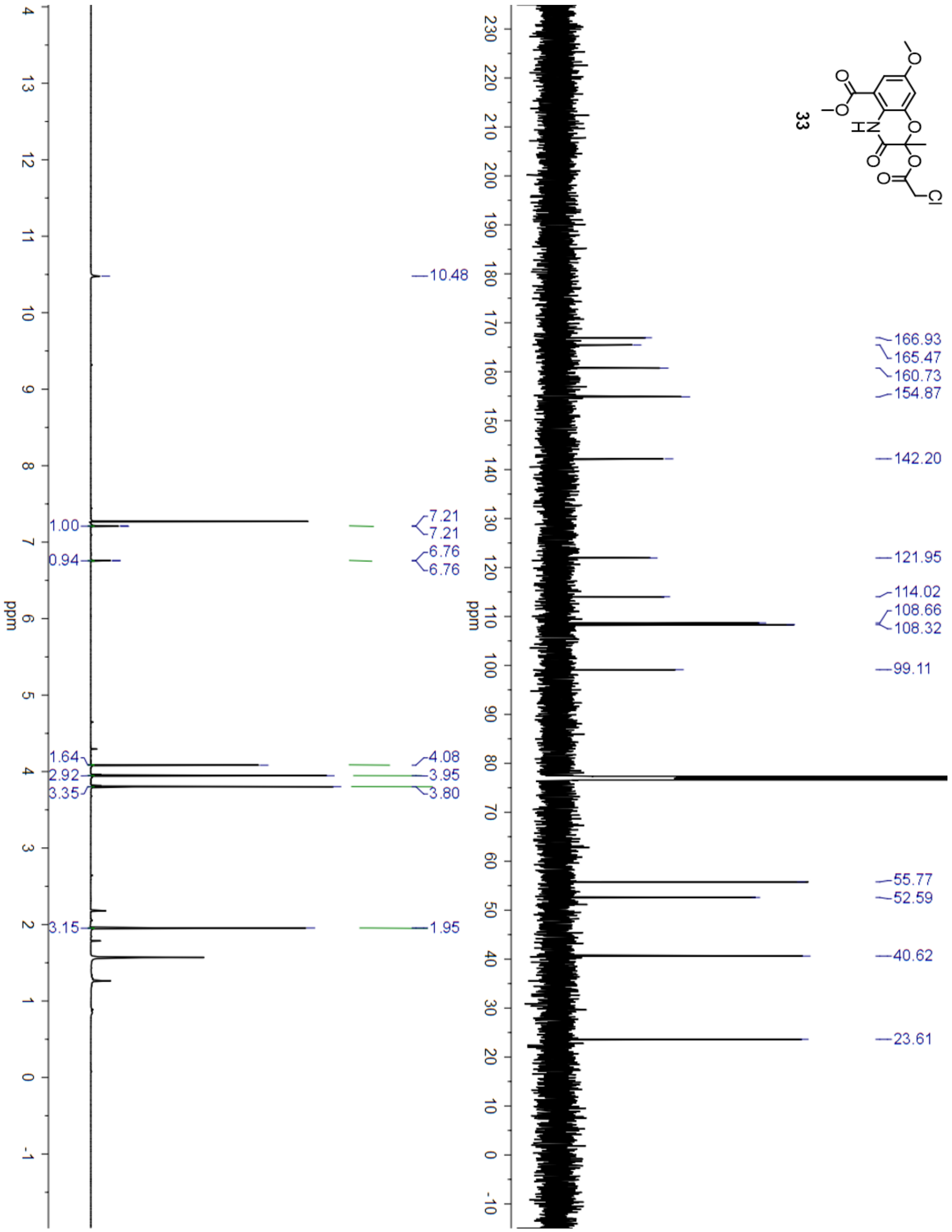
33
ppm
10.48
7.21
7.21
6.76
6.76
4.08
3.95
3.80
1.95
1.00
0.94
1.64
2.92
3.35
3.15
166.93
165.47
160.73
154.87
142.20
121.95
114.02
108.66
108.32
99.11
55.77
52.59
40.62
23.61

nOe spectra of oxazine **13**

Key nOe interactions of irradiated proton (δ_H = 2.91, highlighted in blue) with protons highlighted in red.

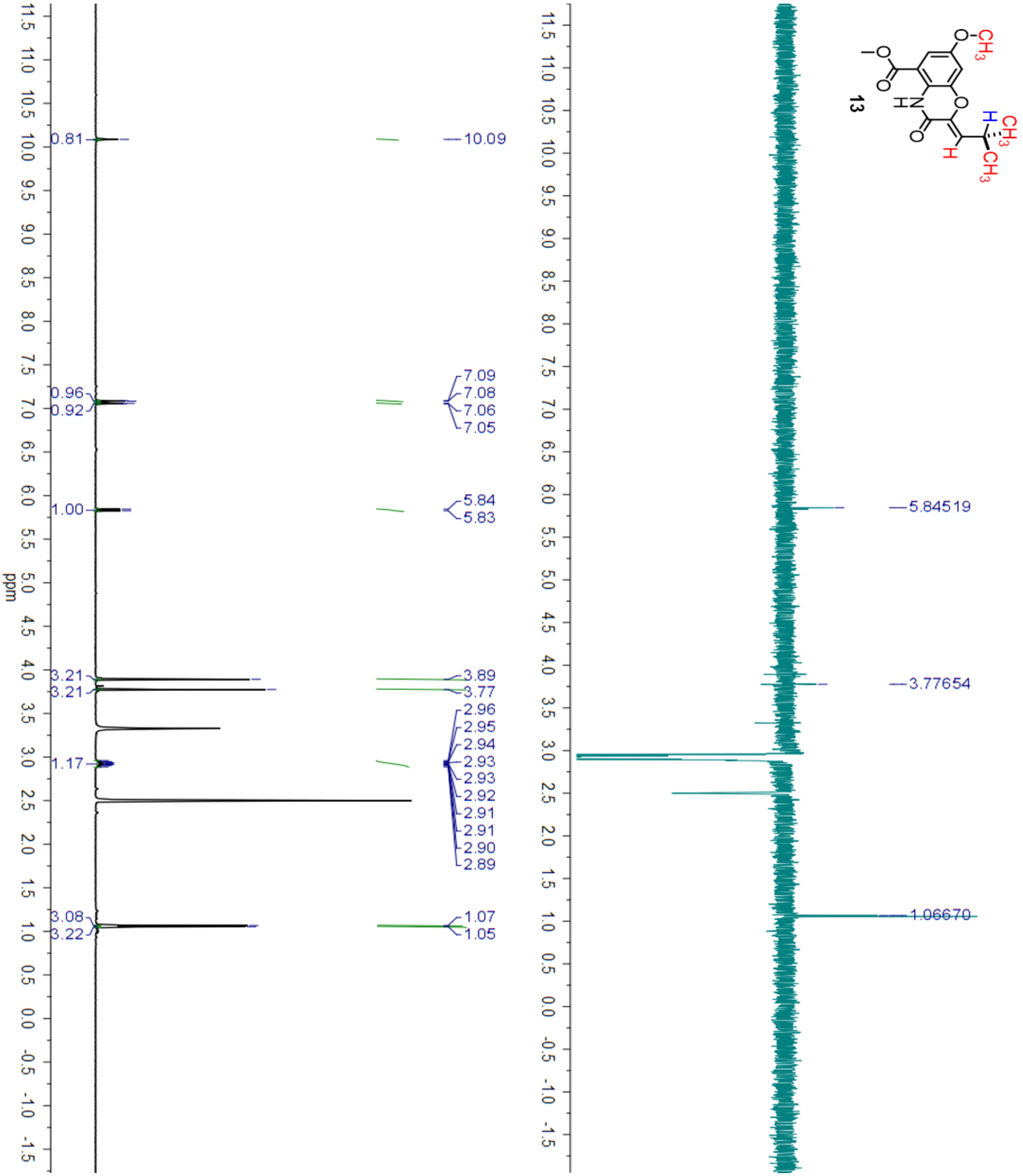

Experimental References

[1] K. C. Peach, W. M. Bray, N. J. Shikuma, N. C. Gassner, R. S. Lokey, F. H. Yildiz, R. G. Linington, *Mol. Biosyst.* **2011**, *7*, 1176–1184.

[2] A. Heydorn, A. T. Nielsen, M. Hentzer, C. Sternberg, M. Givskov, B. K. Ersbøll, S. Molin, *Microbiology* **2000**, *146*, 2395–2407.

[3] W. R. Wong, A. G. Oliver, and R. G. Linington, *Chem. Biol.*, 2012, **19**, 1483–95.

[4] C. J. Schulze, W. M. Bray, M. H. Woerhmann, J. Stuart, R. S. Lokey, and R. G. Linington, *Chem. Biol.*, 2013, **20**, 285–295.

[5] P. E. Zhichkin, X. Jin, H. Zhang, L. H. Peterson, C. Ramirez, T. M. Snyder, H. S. Burton, *Org. Biomol. Chem.* **2010**, *8*, 1287–1289.

[6] F. Heaney, J. Fenlon, P. McArdle, D. Cunningham, *Org. Biomol. Chem.* **2003**, *1*, 1122–1132.

[7] K. C. Peach, A. T. Cheng, A. G. Oliver, F. H. Yildiz, and R. G. Linington, *Chembiochem*, 2013, **14**, 2209–2215.

[8] H. C. J. Ottenheijm, M. W. Tijhuis, *Org. Synth.* **1983**, *61*, 1; (b) A. J.-L. Ayitou, J. Sivaguru, *Chem. Commun.* **2011**, *47*, 2568–2570.
